# Zinc Fingers as Programmable Metalloprotein Scaffolds for Altering Pathogenic Biomolecular Phase Transitions

**DOI:** 10.1002/advs.77886

**Published:** 2026-09-30

**Authors:** Seongmin Park, Yunha Hwang, Jimin Kwak, Yuxi Lin, Hye‐Yeon Park, Eunju Nam, Jiyeon Han, Soyeon Park, Hyunyong Kim, Ju‐Eun Kim, Hyun Goo Kang, Jiyong Park, Young‐Ho Lee, Kyoung‐Shim Kim, Chul‐Ho Lee, Seung Jae Lee, Mi Hee Lim

**Affiliations:** ^1^ Department of Chemistry Korea Advanced Institute of Science and Technology (KAIST) Daejeon Republic of Korea; ^2^ Department of Chemistry Jeonbuk National University Jeonju Republic of Korea; ^3^ Center for Protein Structure and Drug Mechanism Research Korea Basic Science Institute (KBSI) Ochang Republic of Korea; ^4^ Laboratory Animal Resource Center Korea Research Institute of Bioscience and Biotechnology (KRIBB) Daejeon Republic of Korea; ^5^ Department of Physics and Chemistry Daegu Gyeongbuk Institute of Science and Technology (DGIST) Daegu Republic of Korea; ^6^ Department of Applied Chemistry University of Seoul Seoul Republic of Korea; ^7^ Department of Neurology and Research Institute of Clinical Medicine Jeonbuk National University Hospital Jeonju Republic of Korea; ^8^ Center for Catalytic Hydrocarbon Functionalizations Institute for Basic Science (IBS) Daejeon Republic of Korea; ^9^ Bio‐Analytical Science University of Science and Technology (UST) Daejeon Republic of Korea; ^10^ Graduate School of Analytical Science and Technology Chungnam National University Daejeon Republic of Korea; ^11^ Department of Systems Biotechnology Chung‐Ang University (CAU) Gyeonggi Republic of Korea; ^12^ Institute of Molecular Biology and Genetics Jeonbuk National University Jeonju Republic of Korea

**Keywords:** amyloid assembly control, biomolecular phase behavior, protein‐based materials, protein–protein interactions, zinc finger proteins

## Abstract

Tunable control over biomolecular phase transitions provides a powerful blueprint for engineering functional soft materials, yet generalizable protein scaffolds capable of rewiring pathological condensates remain largely unexplored. Here, we demonstrate that zinc finger (ZF) proteins function as programmable interaction units that modulate neurodegenerative amyloid liquid–liquid phase separation, redirect β‐sheet‐rich fibrillation toward nontoxic assemblies, and actively disassemble preformed fibrils. Multiscale investigations spanning molecular, cellular, and organismal levels reveal that tandemly arrayed ZF modules mediate direct, high‐affinity protein–protein interactions, thereby mitigating amyloid pathogenicity. Through modular protein engineering, these protective effects translate in vivo, where a brain‐penetrant ZF variant reduces amyloid deposition, suppresses neuroinflammatory responses, and improves cognitive performance in Alzheimer's disease transgenic mice. Furthermore, sequence–structure–function analyses identify key determinants governing amyloid assembly control, offering mechanistic principles for ZF‐inspired protein engineering. Collectively, this work positions ZF proteins as versatile, chemically programmable platforms for engineering protein‐based materials that regulate biomolecular phase behavior and toxicity, expanding their functional scope beyond traditional genetic regulation toward therapeutic applications.

## Graphical abstract

Modular zinc finger (ZF) metalloproteins function as programmable biomolecular scaffolds that modulate pathogenic amyloid assembly. Through direct protein–protein interactions, ZF arrays alter amyloid phase behavior and redirect β‐sheet fibrillization toward nontoxic aggregates. Modular engineering, in vivo histopathological and behavioral validation, and integrated mechanistic analyses establish design principles for developing ZF‐inspired metalloprotein therapeutics.

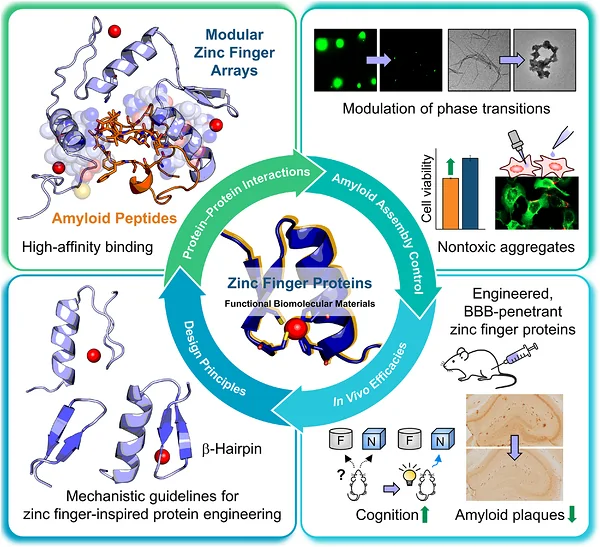

## 1 Introduction

Bioinspired functional platforms offer promising strategies for developing soft materials capable of regulating biomolecular phase behavior and enabling translational biomedical applications [1, 2, 3]. Among biological systems, metalloproteins, constituting approximately one‐third to one‐half of all cellular proteins, represent particularly attractive candidates because of their structural dynamics and diverse chemical reactivities originating from metal‐centered coordination environments [4, 5, 6]. As one of the most prevalent classes of metalloproteins, zinc finger (ZF) proteins utilize zinc as a structural cofactor and play essential roles in orchestrating various biological processes through their modular and multivalent domain organization [7, 8, 9, 10].

By interacting with a wide range of biomolecules, including DNA and RNA, ZF proteins have traditionally been known for their ability to regulate transcriptional and translational processes [10, 11]. Beyond nucleic acid binding, however, the structural versatility of ZF proteins also renders them well suited for engaging multiple protein partners [10, 12, 13, 14, 15]. In classical Cys_2_His_2_‐type ZF modules, the Zn(II)‐centered stabilization of α‐helical and β‐strand motifs provides a balanced combination of rigidity and adaptability that supports the formation of stable protein–protein interfaces [13, 14, 15]. Together with their tandem modular arrangement and metal‐dependent framework, ZF arrays therefore serve as a versatile scaffold for multivalent molecular recognition [7, 13, 14, 15, 16, 17]. Consistent with these characteristics, ZF proteins have recently emerged as important mediators of liquid–liquid phase separation (LLPS) of their binding partners, where interactions with intrinsically disordered regions of biomolecules affect the generation and maturation of condensates [12, 18, 19].

Biomolecular LLPS is increasingly recognized as a critical physicochemical phenomenon underlying amyloid nucleation and aggregation [20, 21, 22]. The formation of concentrated, liquid‐like droplets composed of amyloidogenic peptides or proteins generates localized environments that can facilitate nucleation and the subsequent liquid‐to‐solid phase transition toward amyloid assemblies. These processes contribute to the pathogenesis of neurodegenerative disorders, including Alzheimer's disease (AD), the most common form of dementia [20, 21, 22, 23]. As a representative amyloidogenic peptide associated with AD, the intrinsically disordered amyloid‐β (Aβ; Figure 1a) undergoes LLPS followed by primary nucleation [21, 24]. This initiates characteristic on‐pathway fibrillization, producing oligomers, protofibrils, and mature fibrils that exacerbate pathological cascades by disrupting synaptic plasticity, impairing neuronal signaling, and triggering neuroinflammation [23, 25, 26, 27]. In contrast, redirecting Aβ amyloidogenesis away from canonical fibrillation can yield non‐fibrillar, unstructured aggregates (Figure 1a) that are less toxic, providing a conceptual basis for next‐generation biomaterials designed to reprogram pathological biomolecular assembly [28, 29].

**FIGURE 1:**
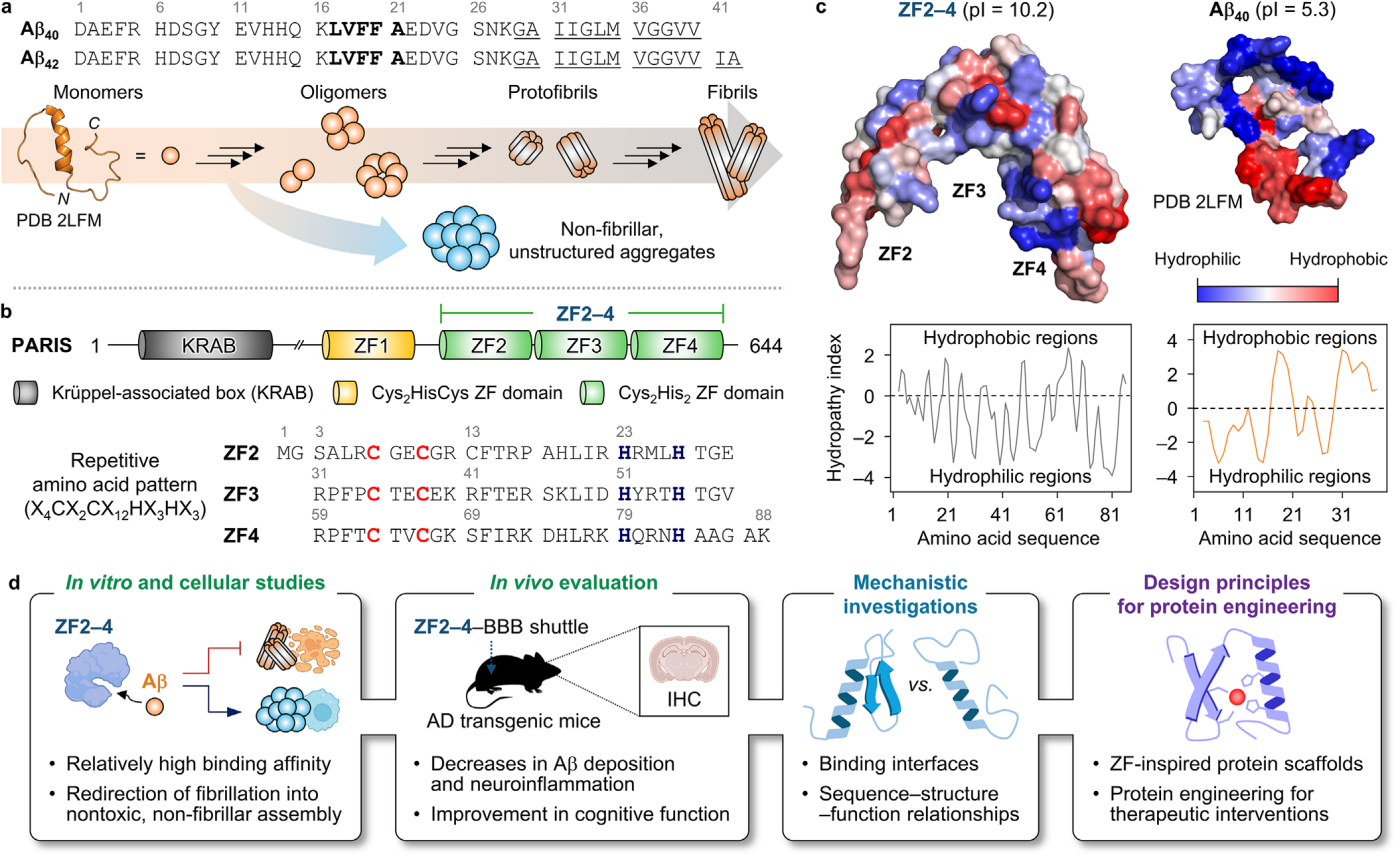
Aβ peptides and ZF2–4 studied in this work. (a) Amino acid sequences of Aβ_40_ and Aβ_42_ as well as their aggregation pathways. The amino acid residues involved in the self‐recognition and *C*‐terminal hydrophobic regions are indicated in bold and underline, respectively. A partially folded Aβ_40_ structure (PDB 2LFM [38]), determined by NMR spectroscopy under aqueous conditions is depicted as a representative monomeric conformation. (b) Domain organization of PARIS with the amino acid sequences of its classical ZF segments (ZF2–4). The cysteine and histidine residues involved in Zn(II) coordination are highlighted in bold red and navy, respectively. (c) Physicochemical properties of ZF2–4 and Aβ_40_. Hydrophilicity and hydrophobicity were calculated using the ExPASy server and mapped on the corresponding structures. (d) Schematic overview of this study summarizing the main findings.

Beyond their well‐established roles in nucleic acid recognition, ZF proteins can also participate in protein–protein interactions and may therefore serve as molecular regulators of biomolecular phase behavior. Whether ZF proteins directly impact neurological pathways by reshaping amyloid assembly through such interactions remains largely unexplored, however. We therefore hypothesized that ZF‐mediated protein–protein interactions with intrinsically disordered amyloids could modulate their phase separation dynamics and, consequently, alter aggregation propensity and associated toxicity. To test this hypothesis, we focused on a tandem array of three Cys_2_His_2_‐type ZF modules (ZF2–4) derived from the endogenous PARIS (Parkin interacting substrate; Figure 1b), a transcriptional mediator that is upregulated under neurodegenerative conditions and implicated in neuronal survival and mitochondrial quality control [30, 31, 32]. Structurally defined features of the ZF2–4 module, including tandemly arranged ZF repeats, a high isoelectric point (pI = 10.2), and an amphiphilic, multivalent molecular surface, suggest the potential for complementary electrostatic and hydrophobic interactions with amphiphilic Aβ peptides (Figure 1c).

Guided by our design rationale (Figure 1d), we investigated the physical interactions between ZF2–4 and Aβ. Integrated computational and biophysical analyses reveal that modular ZF2–4 arrays form relatively stable complexes with Aβ, accompanied by distinct conformational rearrangements. We further examined how these interactions influence Aβ phase separation, aggregation, and toxicity in vitro and in living cells. To establish physiological relevance and advance this system as a biomolecular engineering platform, we engineered and characterized a brain‐penetrant ZF2–4 variant and evaluated its histopathological and behavioral effects in AD transgenic (Tg) mice. Finally, structure‐guided analyses identified key residues and structural motifs within ZF2–4 that govern Aβ binding and aggregation modulation, providing mechanistic principles for ZF‐inspired protein engineering. Overall, this study discovers a previously unrecognized function of ZF proteins in modulating pathogenic biomolecular assembly and introduces new opportunities for developing protein–protein interaction‐based programmable biomaterials for therapeutic interventions.

## 2 Results and Discussion

### 2.1 Preparation of ZF2–4 and Its Direct Interactions with Aβ

As illustrated in Figure 1a, hydrophobic and electrostatic interactions within the *N*‐terminal, self‐recognition, and *C*‐terminal regions of Aβ represent key driving forces governing its assembly and stabilization [23, 25]. Accordingly, the balanced hydrophobic and hydrophilic characteristics of ZF2–4, supported by hydropathy analyses (Figure 1c) [33], suggest its potential to perturb the intra‐ and inter‐molecular interaction networks of Aβ peptides and thereby control their phase behavior through direct molecular engagement. To gain mechanistic insight into the interaction dynamics between ZF2–4 and Aβ, we first examined putative ZF2–4–Aβ_40_ binding modes at the atomistic level using computational approaches. We constructed the heterodimeric interface between these two proteins, prioritizing scenarios involving negative cooperativity [34] rather than higher‐order 1:2 ZF2–4‐to‐Aβ_40_ complexes. Because experimentally resolved structures of ZF2–4 were unavailable, an initial structural model was generated using AI‐assisted prediction methods (AlphaFold2 [35, 36] and AlphaFold3 [37]; Figures S1a and S2). For monomeric Aβ_40_, the solution NMR structure (PDB 2LFM [38]) was employed as the starting conformation (Figure S1b). Considering the intrinsically disordered nature of Aβ_40_ [23], the interaction models were further refined through protein–protein docking and molecular dynamics (MD) simulations [39, 40, 41, 42].

Among the twelve heterodimeric interfaces evaluated (Figures S3–S5), one configuration (Model 11; Figure 2a) maintained a persistent dimeric interface throughout microsecond‐scale MD simulations extending up to 2.5 µs, showing a relatively stable binding mode [43]. In Model 11, the heterodimeric interface spans a contact surface area (CSA) of 2,315 Å^2^ with a calculated binding energy of –11.41 kcal/mol based on molecular mechanics/Poisson‐Boltzmann surface area analysis [42]. Formation of the ZF2–4–Aβ_40_ heterodimer perturbs both inter‐ and intra‐molecular networks within Aβ, including salt bridges, hydrogen bonds, and hydrophobic contacts, thereby providing a mechanistic basis for changing canonical amyloid behavior (Figure 2b–d). In particular, the Asp7 residue of Aβ_40_ forms an ionic interaction (salt bridge) with the Lys78 residue of ZF2–4 (Figure 2b), effectively neutralizing electrostatic charge in the *N*‐terminal region of Aβ_40_ and reducing repulsive interactions that can hinder aggregation [41, 44]. In addition, amino acid residues of ZF2–4 participate in intermolecular hydrogen bonds with key fibril‐forming regions of Aβ_40_, while the aggregation‐prone hydrophobic *C*‐terminal segment of Aβ_40_, particularly residues Val36–Val40, is accommodated within a cleft generated by ZF2–4 and stabilized through extensive hydrophobic and polar contacts (Figure 2b,c) [23, 45]. Binding to ZF2–4 also reorganizes intramolecular hydrogen‐bonding networks within Aβ_40_ (Figure 2d). Collectively, these inter‐ and intra‐molecular interactions stabilize the heterodimeric interface and alter the conformational landscape of Aβ, ultimately influencing its phase behavior (vide infra).

**FIGURE 2:**
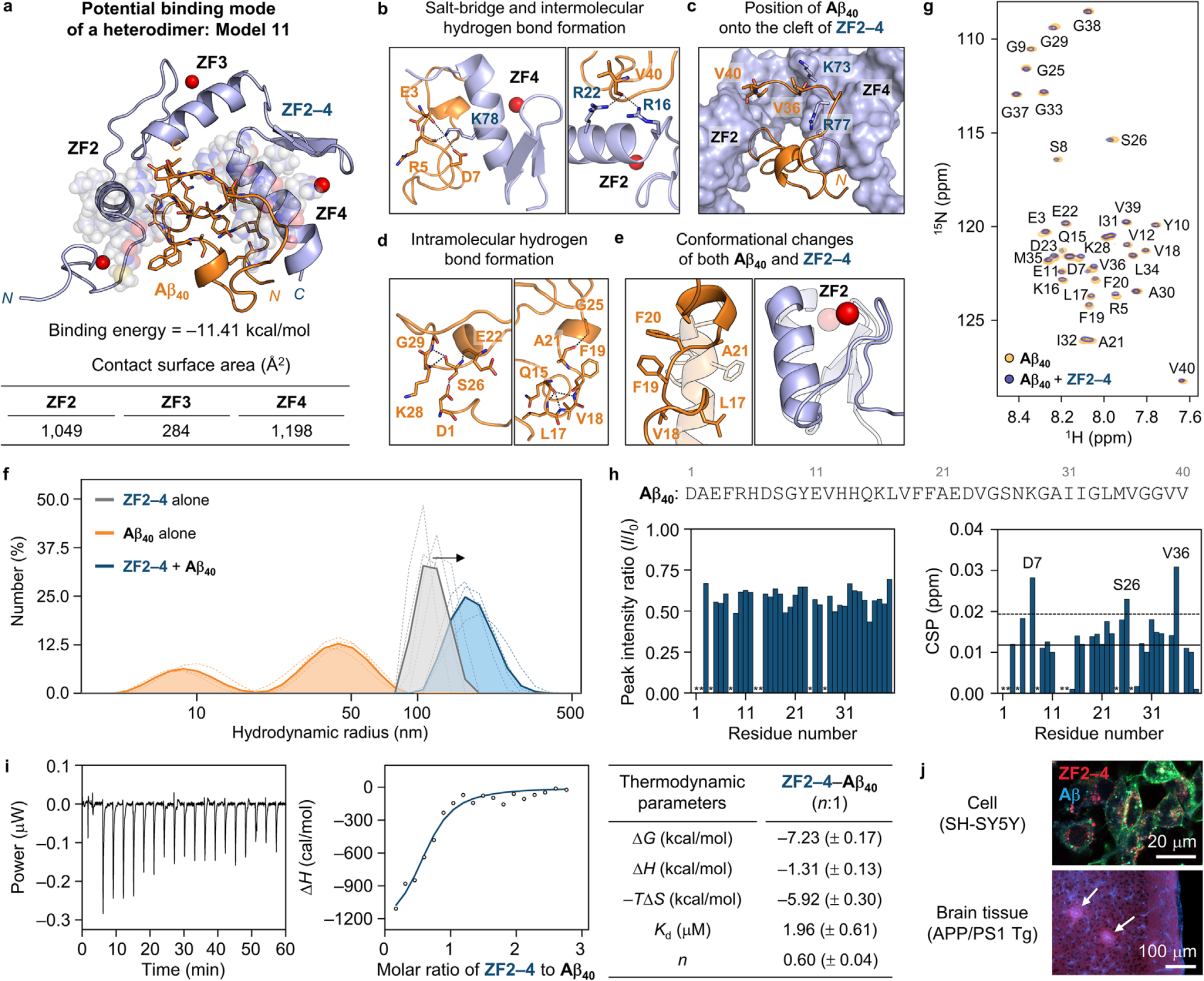
Interactions of ZF2–4 with Aβ_40_. (a) A representative structural model of the ZF2–4–Aβ_40_ heterodimer obtained from MD simulations over 2.5 µs. ZF2–4 and Aβ_40_ are shown in lavender and orange, respectively. (b–e) Detailed structural analysis of Model 11. Possible hydrogen bonds within 3.0 Å are indicated by dashed black lines. The cleft formed by ZF2–4 is displayed in a space‐filling representation. Monomeric structures of ZF2–4 and Aβ_40_ are overlaid with transparency for comparison. (f) Hydrodynamic radii of ZF2–4, Aβ_40_, and their mixture determined by DLS. Conditions: [Aβ_40_] = 100 µM; [ZF2–4] = 100 µM; 20 mM HEPES, pH 7.4, 1 mM 1,4‐dithiothreitol (DTT); 25°C. Data are shown as the averaged solid trace from four independent experiments. (g,h) 2D NMR analyses of ^15^N‐labeled Aβ_40_ upon addition of ZF2–4. The mean CSPs and mean + one standard deviation are indicated by solid and dashed lines, respectively. Individual NMR spectrum of each sample is depicted in Figure S7. Conditions: [^15^N‐labeled Aβ_40_] = 20 µM; [ZF2–4] = 200 µM; 20 mM HEPES, pH 7.4, 1 mM DTT, 10% v/v D_2_O; 10°C. Unresolved peaks are marked with asterisks. (i) Thermodynamic binding properties of ZF2–4 toward Aβ_40_ characterized by ITC. The ITC thermogram was obtained from the titration of ZF2–4 into Aβ_40_, and the corresponding binding isotherm was derived from the integrated heat data. The solid line represents the fitted curve using a one‐set‐of‐sites binding model to determine the thermodynamic parameters. Conditions: [Aβ_40_] = 20 µM; [ZF2–4] = 300 µM; 20 mM HEPES, pH 7.4, 1 mM DTT; 10°C. Error values for the thermodynamic parameters represent the fitting errors. (j) Cellular and ex vivo brain tissue localization of ZF2–4 and Aβ. Aβ species were visualized by immunostaining with an anti‐Aβ antibody (6E10; blue), while ^HF647^ZF2–4 is shown in red. Cell membranes in the cellular experiments are shown in green. Regions showing the spatial co‐localization of ZF2–4 and Aβ are indicated by white arrows. Conditions: (for cellular experiments) [Aβ_40_] = 5 µM; [^HF647^ZF2–4] = 5 µM; (for ex vivo experiments) [^HF647^ZF2–4] = 5 µM. Scale bar = 20 or 100 µm.

Importantly, our MD simulations further revealed conformational changes in Aβ_40_ upon binding to ZF2–4, as illustrated in Figure 2e. In particular, the secondary structure spanning residues His13–Ala21, which includes the self‐recognition region critical for aggregation [23, 25], became substantially disordered relative to its intrinsic α‐helical conformation (Figures 2e and S1b). These results suggest that ZF2–4 alters the aggregation propensity of Aβ, motivating a detailed experimental investigation of ZF2–4 binding and its impact on Aβ_40_ aggregation behavior.

To this end, ZF2–4 and ^15^N‐labeled ZF2–4 were recombinantly expressed in *Escherichia coli* (*E. coli*) and purified as described previously (Figure S6) [32]. To characterize hydrodynamic properties of ZF2–4 and its interactions with Aβ_40_, we performed dynamic light scattering (DLS), two‐dimensional (2D) ^1^H–^15^N heteronuclear single quantum coherence (HSQC) or selective optimized flip‐angle short‐transient heteronuclear multiple‐quantum correlation (SOFAST–HMQC) NMR spectroscopy employing either ^15^N‐labeled ZF2–4 or ^15^N‐labeled Aβ_40_, respectively, isothermal titration calorimetry (ITC), and fluorescence‐quenching measurements with fluorophore‐conjugated Aβ_40_ (^HF488^Aβ_40_) [46].

As anticipated, the hydrodynamic radius increased upon mixing ZF2–4 with Aβ_40_ relative to each component alone, revealing conformational rearrangements associated with complex formation (Figure 2f). In line with this observation, the addition of ZF2–4 to ^15^N‐labeled Aβ_40_ resulted in a global reduction in NMR signal intensity across all amino acid residues, with an average decrease of 41.9 (± 0.9)% in the 2D ^1^H–^15^N SOFAST–HMQC NMR spectra (Figures 2g,h and S7). This signal attenuation reflects the formation of NMR‐invisible assemblies, supporting direct interactions between ZF2–4 and monomeric Aβ in solution and their consequent impact on Aβ aggregation dynamics. In addition, chemical shift perturbations (CSPs) were detected at specific amino acid residues, including Asp7, Ser26, and Val36, consistent with the interaction sites identified in the MD simulations (Figure 2b–d). Complementary 2D ^1^H–^15^N HSQC NMR spectra of purified ^15^N‐labeled ZF2–4 similarly showed reduced peak intensity and CSPs at multiple residues upon incubation with Aβ_40_, providing reciprocal evidence for direct binding between the two proteins (Figure S8).

Furthermore, the isothermal titration of ZF2–4 into monomeric Aβ_40_, after the subtraction of the corresponding heat of dilution for ZF2–4 alone, revealed a spontaneous and exothermic binding process [Δ*G* = −7.23 (± 0.17) kcal/mol; Δ*H* = −1.31 (± 0.13) kcal/mol; *T*Δ*S* = 5.92 (± 0.30) kcal/mol], indicating that the interaction is driven by both favorable enthalpic and entropic contributions (Figure 2i). The dissociation constant (*K*_d_) for the ZF2–4–Aβ_40_ complex was determined to be 1.96 (± 0.61) µM, with an approximate 1:1.7 ZF2–4‐to‐Aβ_40_ binding stoichiometry. Similarly, fluorescence quenching measurements yielded a *K*_d_ value of 0.32 (± 0.01) µM and an approximate binding stoichiometry of 1:1.5 for the ZF2–4–Aβ_40_ interaction (Figure S9). Consistent with the favorable interaction suggested by the MD simulations (–11.41 kcal/mol; Figure 2a), these complementary thermodynamic and fluorometric analyses support a low micromolar binding affinity that is sufficient to directly influence the phase behavior of Aβ_40_ [47, 48].

To further examine whether the ZF2–4–Aβ engagement observed in solution is retained under biologically relevant conditions, we prepared fluorophore‐labeled ZF2–4 (^HF647^ZF2–4) and evaluated its binding to Aβ in cellular and ex vivo systems (Figure 2j). In the cellular system, extracellularly applied ^HF647^ZF2–4 exhibited pronounced co‐localization with immunostained Aβ_40_ at the cell surface, supporting the ability of ZF2–4 to associate with Aβ in a cellular environment. Importantly, the Aβ‐associated localization of ^HF647^ZF2–4 was also observed in the brain tissue. Brain sections obtained from AD amyloid precursor protein/presenilin‐1 (APP/PS1) Tg mice were incubated with ^HF647^ZF2–4 and subsequently immunostained for Aβ deposits. In the cortical regions, ^HF647^ZF2–4 showed spatial co‐localization with Aβ deposits, further confirming that the Aβ‐associating capability of ZF2–4 is retained within the brain tissue. Taken together, these computational and experimental results demonstrate that ZF2–4 binds with low micromolar affinity to the regions of Aβ_40_ critical for fibrillation, inducing conformational rearrangements that alter its biomolecular phase transitions (vide infra).

### 2.2 Effects of ZF2–4 on Aβ LLPS and Aggregation In Vitro

To elucidate how direct ZF2–4–Aβ interactions influence Aβ amyloidogenesis, we first examined their effects on Aβ LLPS. As a precursor to amyloid fibrillation [20, 21, 22], Aβ_40_ formed protein‐dense liquid droplets under physiological conditions (Figure 3a) [20, 21]. In the presence of equimolar ZF2–4, the resulting droplets were substantially smaller, exhibiting a 93.7 (± 13.1)% decrease in size relative to Aβ_40_ alone. This pronounced reduction indicates that ZF2–4–Aβ interactions perturb the intermolecular networks required for Aβ LLPS. These observations suggest that ZF2–4 inhibits condensate maturation and/or promotes a liquid‐to‐solid‐like phase transition toward non‐fibrillar, unstructured assemblies, thereby reshaping the assembly behavior of Aβ [20, 21].

**FIGURE 3:**
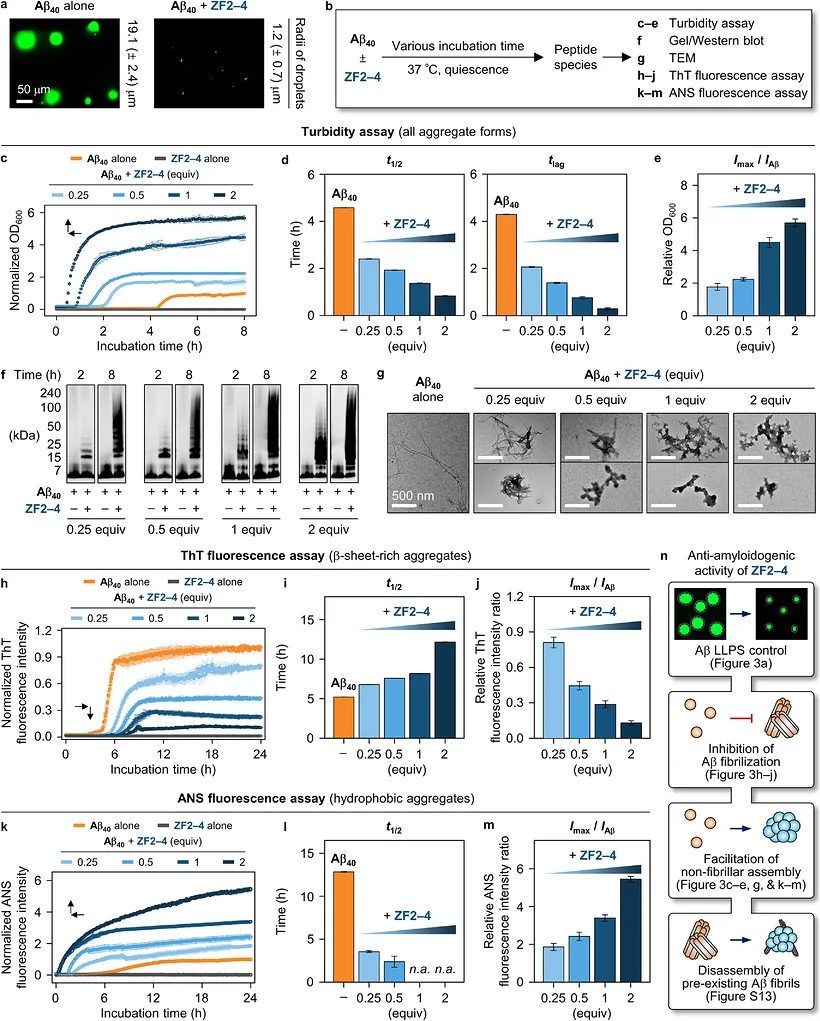
Effects of ZF2–4 on Aβ_40_ amyloidogenesis. (a) LLPS behavior of Aβ_40_ in the absence and presence of ZF2–4 visualized by confocal microscopy. Images were obtained from five randomly selected fields for each condition. The radii of liquid droplets were quantified to determine size distributions. Conditions: [Aβ_40_] = 100 µM (10% v/v ^HF488^Aβ_40_); [ZF2–4] = 100 µM; 20 mM KPi, pH 7.4, 75 mM NaCl, 20% w/v PEG, 0.02% w/v NaN_3_, 1 mM DTT; 37°C; quiescence. Scale bar = 50 µm. (b) Schematic illustration of the aggregation experiments. (c–e) Aggregation kinetics of Aβ_40_ incubated with increasing concentrations of ZF2–4 measured by the turbidity assay. Normalized absorbance intensities were plotted as a function of incubation time and fitted to obtain kinetic parameters (*t*_1/2_ and *t*_lag_). Conditions: [Aβ_40_] = 100 µM; [ZF2–4] = 25, 50, 100, and 200 µM; 20 mM HEPES, pH 7.4, 150 mM NaCl, 1 mM DTT; 37°C; quiescence; λ_abs_ = 600 nm. Data are presented as mean ± s.e.m. of three independent experiments. (f) Size distribution of the resultant Aβ_40_ species analyzed by tricine–PAGE followed by Western blotting using an anti‐Aβ antibody (6E10). Unprocessed gel images are presented in Figure S11. Conditions: [Aβ_40_] = 100 µM; [ZF2–4] = 25, 50, 100, and 200 µM; 20 mM HEPES, pH 7.4, 150 mM NaCl, 1 mM DTT; 37°C; quiescence. (g) Morphology of the resultant peptide aggregates formed after 8 h incubation visualized by TEM. Conditions: [Aβ_40_] = 100 µM; [ZF2–4] = 25, 50, 100, and 200 µM; 20 mM HEPES, pH 7.4, 150 mM NaCl, 1 mM DTT; 37°C; quiescence. Scale bar = 500 nm. (h–j) Formation kinetics of β‐sheet‐rich aggregates of Aβ_40_ in the absence and presence of ZF2–4 monitored by the ThT fluorescence assay. (k–m) Aggregation kinetics of Aβ_40_ with and without ZF2–4 detected by the ANS fluorescence assay. In both assays, normalized fluorescence intensities were plotted as a function of incubation time and fitted to determine kinetic parameters. Conditions: [Aβ_40_] = 20 µM; [ZF2–4] = 5, 10, 20, and 40 µM; [ThT] = 20 µM; [ANS] = 100 µM; 20 mM HEPES, pH 7.4, 150 mM NaCl, 1 mM DTT; 37°C; quiescence; (for the ThT fluorescence assay) λ_ex_ = 440 nm; λ_em_ = 490 nm; (for the ANS fluorescence assay) λ_ex_ = 350 nm; λ_em_ = 480 nm. *n.a*., not available. Data are presented as mean ± s.e.m. of three independent experiments. (n) Summary of ZF2–4‐mediated anti‐amyloidogenic activity in vitro.

Building on these LLPS results, we next monitored Aβ_40_ aggregation kinetics in the absence and presence of ZF2–4 using the turbidity assay, which reports the formation of total aggregates (Figure 3b) [46]. As shown in Figure 3c, Aβ_40_ alone displayed typical sigmoidal aggregation behavior, reaching a plateau after approximately 5 h following a distinct lag phase and elongation period. In contrast, equimolar ZF2–4 markedly accelerated aggregate formation, as indicated by reduced lag time (*t*_lag_) and half‐time (*t*_1/2_) values (Figure 3d). The corresponding absorbance intensity also exceeded that of Aβ_40_ alone (Figure 3e and Table S1), confirming enhanced aggregate production. Furthermore, both sub‐ and supra‐equimolar concentrations of ZF2–4 increased aggregate formation in a concentration‐dependent manner (Figure 3c–e), demonstrating that ZF2–4 actively modulates Aβ_40_ aggregation pathways. It should be noted that ZF2–4 alone did not undergo self‐assembly under these experimental conditions.

To further examine the molecular weight distribution of the aggregates, we analyzed Aβ_40_ species (4–240 kDa) after incubation with and without ZF2–4 using gel electrophoresis and Western blotting (gel/Western blot) with an anti‐Aβ antibody (6E10). As depicted in Figure 3f, Aβ_40_ alone showed a relatively simple banding pattern, whereas incubation with 0.25 equiv of ZF2–4 for 8 h generated a distinct band at ca. 15 kDa together with pronounced smearing extending from ca. 15 kDa to 240 kDa, indicative of heterogeneous aggregate formation. This aggregation pattern persisted at higher ZF2–4 concentrations. The band at ca. 15 kDa was tentatively assigned to a 1:1 ZF2–4‐to‐Aβ_40_ complex and was further supported by tricine–PAGE analysis using purified fluorophore‐labeled ZF2–4 (^HF555^ZF2–4; Figure S10). In these experiments, a band at ca. 15 kDa was detected in both tricine–PAGE (^HF555^ZF2–4) and Western blotting (6E10) analyses, accompanied by a gradual decrease in the intensity of the ca. 10 kDa band corresponding to free ZF2–4, consistent with complex formation. The morphologies of the resultant aggregates were further examined by transmission electron microscopy (TEM) (Figure 3g). In the presence of sub‐equimolar ZF2–4, Aβ_40_ assemblies appeared as a heterogeneous mixture of fibrils and non‐fibrillar, unstructured assemblies, whereas Aβ_40_ alone predominantly formed linear fibrils. At higher ZF2–4 concentrations, large non‐fibrillar aggregates were observed without detectable fibrils even after 8 h of incubation.

Additionally, to probe the production of non‐fibrillar structures, we analyzed changes in the secondary structure of Aβ_40_ using circular dichroism (CD) spectroscopy (Figure S12). An Aβ_40_‐to‐ZF2–4 molar ratio of 1:0.25 was selected to minimize spectral contributions from ZF2–4. Under control conditions, Aβ_40_ transitioned from a random coil to a β‐strand‐rich structure within 4 h, as evidenced by a shift of the local minimum from 202 nm to 225 nm. In contrast, in the presence of ZF2–4, Aβ_40_ retained a predominantly random coil conformation even after 6 h, indicating delayed β‐sheet generation.

We next quantified β‐sheet‐rich Aβ_40_ aggregate formation using the thioflavin‐T (ThT) fluorescence assay [46]. In the absence of ZF2–4, Aβ_40_ showed typical nucleation‐dependent fibrilization with the *t*_lag_ and *t*_1/2_ values of 4.53 (± 0.02) h and 5.21 (± 0.01) h, respectively (Figure 3h). Different from Aβ_40_ alone, ZF2–4 induced a pronounced, concentration‐dependent prolongation of aggregation kinetics (Figure 3i), while the maximum ThT fluorescence intensity was reduced in a dose‐dependent manner (Figure 3j). These results suggest the suppression of β‐sheet‐rich fibrillization.

To analyze aggregate structures, as depicted in Figure 3k, we employed the 8‐anilino‐1‐naphthalenesulfonate (ANS) fluorescence assay, which increases in intensity upon binding to hydrophobic environments [49]. Because unstructured aggregates expose larger hydrophobic surfaces than mature fibrils, ANS fluorescence profiles can distinguish fibrillar from non‐fibrillar aggregation pathways [49]. As expected, Aβ_40_ alone exhibited sigmoidal kinetics with the *t*_lag_ and *t*_1/2_ values of 9.20 (± 0.03) h and 12.83 (± 0.03) h, respectively. In contrast, Aβ_40_ aggregation in the presence of ZF2–4 showed relatively faster kinetics (Figure 3l), consistent with the formation of non‐fibrillar, unstructured aggregates. Moreover, the maximum ANS fluorescence intensity increased in a ZF2–4 concentration‐dependent manner (Figure 3m), reflecting greater exposure of hydrophobic surfaces. Collectively, these findings reveal that ZF2–4 redirects Aβ_40_ aggregation away from canonical fibrillization pathways toward non‐fibrillar assembly routes.

Moving forward, in addition to Aβ_40_, we investigated whether ZF2–4 similarly modulates the aggregation propensity of Aβ_42_, another major Aβ isoform with distinct aggregation properties [23, 25, 50]. As presented in Figure S13a–c, sub‐equimolar concentrations of ZF2–4 delayed and attenuated Aβ_42_ fibrillization compared with Aβ_42_ alone, as analyzed by the ThT fluorescence assay. TEM images revealed that the resultant assemblies consisted of mixed populations of fibrils and non‐fibrillar structures or predominantly unstructured aggregates lacking discernible fibrils (Figure S13d). Increasing the amount of ZF2–4 did not enhance ThT fluorescence even after 24 h of incubation but instead yielded large non‐fibrillar, unstructured aggregates. These observations indicate that ZF2–4 similarly changes the aggregation pathways of both Aβ_40_ and Aβ_42_.

Finally, after establishing that ZF2–4 redirects freshly prepared Aβ toward non‐fibrillar, unstructured aggregation pathways, we assessed whether ZF2–4 could also disassemble preformed Aβ_40_ aggregates or modulate their subsequent growth (Figure S14). Upon treatment with ZF2–4, highly branched, mature Aβ_40_ fibrils were converted into shorter and thinner fibrillar fragments together with non‐fibrillar, less structured assemblies. Taken together, as summarized in Figure 3n, these findings demonstrate that ZF2–4 controls both the formation of nascent Aβ aggregates and the disassembly of pre‐existing amyloid fibrils, with the extent of modulation depending on the ZF2–4‐to‐Aβ stoichiometry, consistent with the binding behavior of ZF2–4 toward Aβ (vide supra). Because non‐fibrillar Aβ assemblies are reported to exhibit lower cytotoxicity than mature fibrils [51, 52, 53], these aggregation shifts provide a mechanistic basis for ZF2–4‐mediated attenuation of Aβ‐associated toxicity (vide infra).

### 2.3 Impact of ZF2–4 on Aβ‐Induced Toxicity in Living Cells

Given that ZF2–4 redirects Aβ LLPS and aggregation toward non‐fibrillar assemblies, which are reported to be less cytotoxic [51, 52, 53], we investigated whether this modulation attenuates Aβ_40_‐induced toxicity in living cells. Two complementary experimental approaches were employed to investigate the influence of ZF2–4 on the toxicity associated with Aβ under distinct conditions: (Method I) extracellular exposure and (Method II) intracellular delivery, as described in Figure 4a. Under extracellular conditions, the interactions between Aβ species and cell membranes can trigger cytotoxicity through mechanisms, such as receptor blockade and ion‐channel formation [54, 55]. To test this effect, Aβ_40_ samples pre‐incubated with varying ZF2–4‐to‐Aβ_40_ ratios for 8 h were applied to human neuroblastoma SH‐SY5Y cells for 48 h. Cell viability and membrane interactions were assessed using the MTT assay [MTT = 3‐(4,5‐dimethylthiazol‐2‐yl)‐2,5‐diphenyltetrazolium bromide] and immunocytochemistry (ICC), respectively. Relative to Aβ_40_ alone, cytotoxicity was significantly mitigated by 15.4 (± 5.0) – 22.1 (± 2.9)% in the presence of ZF2–4 in a concentration‐dependent manner (Figure 4b). ZF2–4 itself did not exhibit cytotoxicity under our experimental conditions.

**FIGURE 4:**
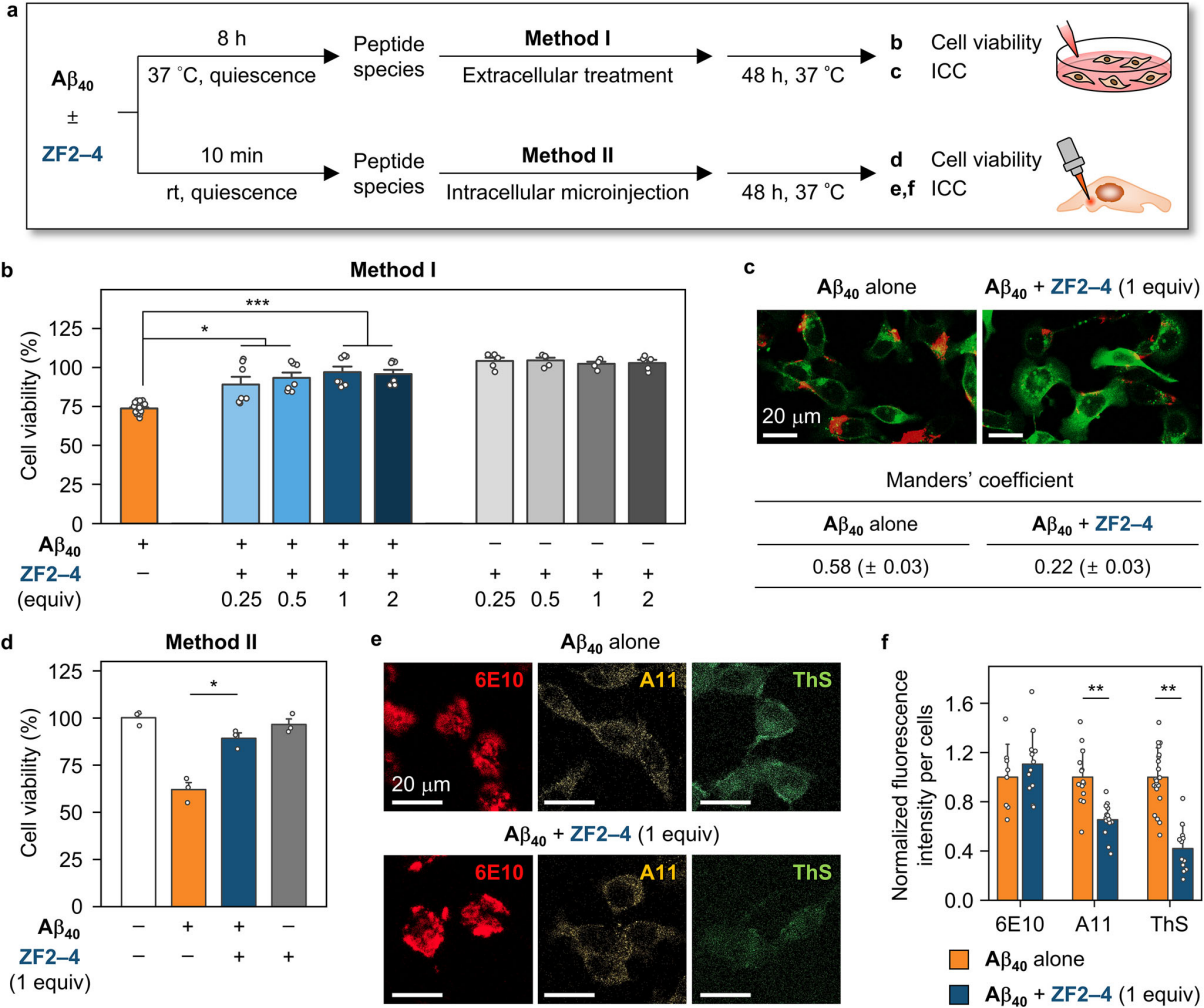
Influence of ZF2–4 on the toxicity induced by Aβ_40_ in living cells. (a) Schematic illustration of the live‐cell experimental design. (b) Viability of SH‐SY5Y cells extracellularly treated with Aβ_40_, ZF2–4, or their mixtures measured by the MTT assay. Cell viability (%) was calculated relative to that of cells added with an equivalent amount of the buffered solution. Conditions: [Aβ_40_] = 10 µM; [ZF2–4] = 2.5, 5, 10, and 20 µM; 20 mM HEPES, pH 7.4, 150 mM NaCl; 37°C; quiescence; SH‐SY5Y cells that were non‐differentiated; incubation for 48 h. *P < 0.1, ***P < 0.001; *n* = 3; Student's *t*‐test. Data are presented as mean ± s.e.m. (c) Live‐cell localization of extracellularly added Aβ_40_ in the absence and presence of ZF2–4 analyzed by ICC studies. Conditions: [Aβ_40_] = 10 µM; [ZF2–4] = 10 µM; 20 mM HEPES, pH 7.4, 150 mM NaCl; 37°C; quiescence; incubation for 48 h. Scale bar = 20 µm. Data are presented as mean ± s.e.m. of three independent experiments. (d) Survival of cells microinjected with Aβ_40_, ZF2–4, or their mixture. Cell survival was quantified as described in the Experimental Methods. More than 250 cells were microinjected for each sample to ensure that at least 100 successfully microinjected cells were available for quantitative analyses. Conditions: [Aβ_40_] = 10 µM; [ZF2–4] = 10 µM; [Texas Red‐labeled dextran] = 0.5 mg/mL; 20 mM HEPES, pH 7.4, 150 mM NaCl; incubation for 48 h. *P < 0.1; *n* = 3–5; Student's *t*‐test. Data are presented as mean ± s.e.m. (e,f) Confocal fluorescence images of cells intracellularly injected with Aβ_40_ with and without ZF2–4. Aβ_40_ species were detected by immunostaining with primary antibodies 6E10 (anti‐Aβ antibody; red) and A11 (anti‐oligomer antibody; orange) with fluorescent‐conjugated secondary antibodies or ThS staining for Aβ fibrils (green). Fluorescence intensities were quantified from nine or more randomly selected fields per condition. Conditions: [Aβ_40_] = 10 µM; [ZF2–4] = 10 µM; 20 mM HEPES, pH 7.4, 150 mM NaCl; incubation for 48 h. Scale bar = 20 µm. **P < 0.01; *n* = 34–58; Student's *t*‐test. Data are presented as mean ± s.e.m.

ICC analyses further supported these findings by visualizing the interactions between Aβ_40_ species and cellular membranes in the absence and presence of ZF2–4 (Figure 4c). Cells treated with Aβ_40_ species produced with and without ZF2–4 were stained using a fluorescent membrane dye and a fluorophore‐conjugated anti‐Aβ antibody (6E10). Co‐localization was quantified by the Manders' coefficient, which measures the fraction of one signal overlapping with another [54]. Notably, Aβ_40_ species formed in the presence of ZF2–4 displayed a 62.1 (± 7.3)% decrease in membrane co‐localization relative to Aβ_40_ alone. These observations confirm that ZF2–4 diminishes the interactions between Aβ_40_ species and cell membranes, demonstrating its capability to redirect Aβ aggregation toward less cytotoxic assemblies.

We next performed microinjection experiments to determine the impact of ZF2–4 on intracellular Aβ_40_‐induced cytotoxicity, as depicted in Figure 4d. Solutions containing either ZF2–4, Aβ_40_, or their combination were mixed with Texas Red‐labeled dextran as an injection marker and directly microinjected into live cells. Samples were not pre‐incubated prior to injection to avoid the formation of large aggregates that could clog the microinjection capillary. Intracellular delivery of Aβ_40_ in the presence of ZF2–4 resulted in a 27.2 (± 4.7)% increase in cell survival compared with cells injected with Aβ_40_ alone.

Moreover, to verify whether ZF2–4 also modulates Aβ_40_ aggregation within the intracellular environment, aggregate formation was analyzed by immunostaining using an anti‐oligomer antibody (A11) and thioflavin‐S (ThS), which can detect Aβ oligomers and fibrils, respectively (Figure 4e) [46, 56]. Similar to our in vitro aggregation studies (vide supra), cells injected with Aβ_40_ in the presence of ZF2–4 exhibited significant reductions in oligomers and fibrils by 34.6 (± 7.0)% and 58.0 (± 7.4)%, respectively (Figure 4f). Because A11‐positive oligomers and ThS‐detectable protofibrils are strongly related to cytotoxicity [55, 56, 57, 58], these results indicate that ZF2–4 promotes the generation of less toxic Aβ_40_ assemblies within the intracellular milieus. Overall, these cellular studies manifest that ZF2–4 attenuates Aβ‐associated cytotoxicity by reducing the interactions between Aβ species and cell membranes and suppressing the formation of intracellular oligomers and fibrils. These findings motivated further evaluation of ZF2–4's protective activity in vivo (vide infra).

### 2.4 Modular Engineering, Characterization, and In Vivo Evaluation of a Brain‐Penetrant ZF2–4 Variant (ZF2–4–P9)

The in vitro and cellular studies illustrated above revealed a previously unrecognized protein–protein interaction‐mediated function of modular ZF2–4 in controlling Aβ phase transitions and associated toxicity. We therefore sought to enhance its bioavailability and evaluate whether its activity could translate to the organismal level, with particular attention to overcoming the blood‐brain barrier (BBB), a major challenge for protein‐based therapeutics. To address the limited BBB permeability of native ZF2–4, we designed a brain‐penetrant variant, ZF2–4–P9, by conjugating a BBB shuttle peptide (CLWRPAADC; P9) [59] to the *C*‐terminus of ZF2–4. The resulting construct was recombinantly expressed in *E. coli*, purified to homogeneity, and tested for BBB permeability using a permeability coefficient (–log*P*_e_) determined by a parallel artificial membrane permeability assay adapted for the BBB (PAMPA–BBB) (Figure S15a–c). As intended, ZF2–4–P9 exhibited increased brain permeability, with a –log*P*_e_ value below the established threshold of 5.4, whereas native ZF2–4 remained above this cutoff (Figure S15c) [60]. Furthermore, the in vivo brain uptake and biodistribution of ZF2–4–P9 in major organs were evaluated in C57BL/6J mice following intraperitoneal (*i.p*.) administration at 3 mg/kg. As summarized in Table S2, ZF2–4–P9 was detected within 10 min post‐administration in the brain [6.2 (± 1.2)%], liver [1.4 (± 0.2)%], kidney [7.6 (± 2.9)%], and plasma [1.5 (± 0.2)%]. Consistent with the PAMPA–BBB results, these in vivo biodistribution data demonstrate that systemically administered ZF2–4–P9 reaches brain tissue and further support its brain‐penetrating capability. Notably, the organ‐to‐plasma ratio of ZF2–4–P9 was greater than 1.0 in both the brain and kidney, whereas its level in the liver was comparable to that in plasma. Importantly, ZF2–4–P9 showed no detectable toxicity in SH‐SY5Y cells at concentrations up to 100 µm (Figure S15d), supporting its suitability for subsequent in vivo evaluation.

To investigate the in vivo efficacy of ZF2–4–P9, we employed an AD APP/PS1 Tg mouse model carrying double mutations in the APP [Swedish mutation (K670N/M671L)] and PS1 (PSEN1ΔE9) genes [61]. These mutations accelerate Aβ overproduction, leading to enhanced fibrillar deposition and progressive cognitive impairment. ZF2–4–P9 was administered intraperitoneally at a dose of 3 mg/kg once daily to five‐month‐old APP/PS1 Tg mice for 38 consecutive days (Figure 5a). Throughout the treatment period, body weight and locomotor activity remained stable in ZF2–4–P9‐treated APP/PS1 Tg mice as well as in vehicle (Veh)‐treated wild‐type (WT) and APP/PS1 Tg control groups (Figure S16), indicating good tolerability and systematic stability of the administrated protein.

**FIGURE 5:**
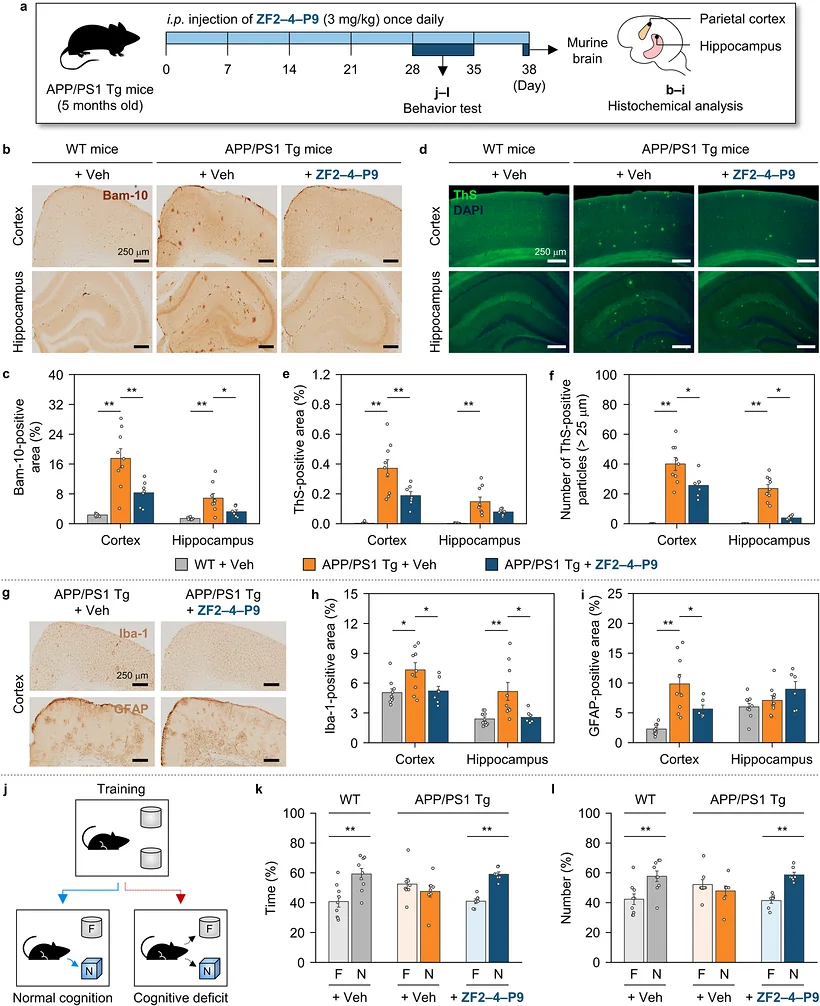
Effects of ZF2–4–P9 on Aβ aggregate deposition, neuroinflammation, and cognitive impairment in APP/PS1 Tg mice. (a) Schematic overview of the in vivo experimental design. (b–f) Analyses of Aβ aggregate deposition in the parietal cortices and hippocampi of Veh‐treated WT, Veh‐treated APP/PS1 Tg, and ZF2–4–P9‐treated APP/PS1 Tg mice. Representative images obtained by immunostaining with anti‐Aβ (Bam‐10) or staining with ThS, together with DAPI staining, are presented. Note that DAPI stains both cellular nuclei and fibrillar Aβ [63]. Scale bar = 250 µm. Animal number: *n* = 10 for Veh‐treated WT mice; *n* = 9 for Veh‐treated APP/PS1 Tg mice; *n* = 7 for ZF2–4–P9‐treated APP/PS1 Tg mice. *P < 0.05, **P < 0.01; one‐way ANOVA followed by Tukey–Kramer's multiple‐comparison test. Data are presented as mean ± s.e.m. (g–i) Quantification of microglial and astrocytic activation in the parietal cortices and hippocampi. Representative images visualized by immunostaining with anti‐Iba‐1 (microglia) and anti‐GFAP (astrocytes) antibodies are shown in (g) and Figure S18. Scale bar = 250 µm. Animal number: *n* = 10 for Veh‐treated WT mice; *n* = 9 for Veh‐treated APP/PS1 Tg mice; *n* = 7 for ZF2–4–P9‐treated APP/PS1 Tg mice. *P < 0.05, **P < 0.01; one‐way ANOVA followed by Tukey–Kramer's multiple‐comparison test. Data are presented as mean ± s.e.m. (j–l) Cognitive performance assessed by the novel object recognition test. The exploration time (%) and number of exploration events (%) at familiar (F) and novel (N) objects are indicated. Animal number: *n* = 10 for Veh‐treated WT mice; *n* = 9 for Veh‐treated APP/PS1 Tg mice; *n* = 7 for ZF2–4–P9‐treated APP/PS1 Tg mice. **P < 0.01; Student's *t*‐test. Data are presented as mean ± s.e.m.

Following the 38‐day treatment regimen, we examined the effects of ZF2–4–P9 on Aβ aggregate deposition, neuroinflammation, and cognitive dysfunction (Figure 5a). Administration of ZF2–4–P9 resulted in a pronounced reduction in Aβ plaque burden, as evidenced by a 52.7 (± 16.3) – 52.9 (± 19.6)% decrease in anti‐Aβ (Bam‐10) [62]‐immunoreactive deposits in both the parietal cortices and hippocampi compared with Veh‐treated APP/PS1 Tg controls (Figure 5b,c). Consistent with these findings, ThS staining, which selectively labels β‐sheet‐rich fibrillar aggregates [54, 59], revealed a 2.0 (± 0.2)‐fold reduction in cortical Aβ fibril accumulation following ZF2–4–P9 treatment (Figure 5d,e). The abundance of large fibrillar aggregates was also markedly diminished across hippocampal subfields (Figure 5d,f). These results demonstrate that the modulatory activity of ZF2–4 observed in vitro, namely, the disruption of preformed fibrils and suppression of further aggregation, is effectively recapitulated in vivo. This fibril‐inhibiting capability likely contributes to the attenuation of downstream pathological processes, underscoring the therapeutic potential of ZF2–4–P9 in mitigating Aβ‐associated pathology. To further evaluate Aβ species biochemically, brain homogenates were subjected to Western blot analysis using an anti‐Aβ antibody (6E10). Compared with Veh‐treated APP/PS1 Tg mice, ZF2–4–P9‐treated mice exhibited reduced Aβ‐immunoreactive signal intensities, particularly at ca. 4, 8, and 24 kDa, consistent with monomeric, dimeric, and hexameric Aβ species, respectively (Figure S17). These biochemical findings complement the histological observations and indicate that ZF2–4–P9 treatment is associated with changes in both fibrillar Aβ deposits and lower‐molecular‐weight, gel‐resolvable Aβ species in vivo.

Moreover, to assess the impact of ZF2–4–P9 on neuroinflammatory responses, we investigated microglial and astrocytic activation, two key mediators of neuroinflammation, by immunohistochemical staining using anti‐Iba‐1 and anti‐GFAP antibodies [61]. In ZF2–4–P9‐treated APP/PS1 Tg mice, microglial activation was reduced by 29.0 (± 11.7)% in the cortex and 50.1 (± 18.1)% in the hippocampus relative to Veh‐treated Tg controls (Figures 5g,h and S18). Although astrocytic activation in the hippocampus did not differ substantially between WT and Tg control groups, cortical astrocytic activation was decreased following ZF2–4–P9 treatment (Figures 5g,i and S18). Notably, plaque‐associated microglial localization remained comparable between Veh‐treated and ZF2–4–P9‐treated APP/PS1 Tg mice, with no significant changes observed in the expression of representative inflammatory cytokine genes, including *Il‐1*β, *iNos*, *Tnf‐α*, *Il‐6*, *Il‐10*, and *Tgf‐*β (Figures S19 and S20). These observations suggest that the attenuation of neuroinflammatory responses is more likely a consequence of the decreased Aβ plaque burden rather than an independent immunomodulatory effect of ZF2–4–P9.

Therefore, these histological findings confirm that ZF2–4–P9 effectively suppresses Aβ‐associated glial activation, thereby mitigating chronic neuroinflammation in vivo. We next examined whether these pathological improvements were accompanied by functional benefits at the behavioral level. In the novel object recognition test, ZF2–4–P9‐treated APP/PS1 Tg mice exhibited significantly increased exploration time and visit frequency toward the novel object compared with Veh‐treated APP/PS1 Tg controls (Figure 5j–l). These behavioral indices were comparable to those observed in WT mice, indicating an improvement in recognition memory following ZF2–4–P9 administration. While this improvement in recognition memory was reproducibly observed across independent experiments, statistically significant improvements were not detected in the Y‐maze or Morris water maze under the present experimental conditions. Thus, the further molecular optimization of the current ZF scaffold will likely be required to achieve broader therapeutic efficacy across multiple cognitive domains.

Taken together, through modular engineering of a BBB‐permeable ZF2–4 variant and validation of its in vivo efficacy, we demonstrate that this ZF‐based construct alleviates hallmark pathological, inflammatory, and behavioral phenotypes in AD Tg mice. Blood chemistry analyses further indicated no detectable toxicity in hepatic, renal, biliary, cardiac, or muscular functions across all experimental groups, including ZF2–4–P9‐treated mice (Figure S21). These findings support the biocompatibility of ZF2–4–P9 and highlight the translational potential of modular ZF proteins as programmable protein‐based therapeutic platforms.

### 2.5 Mechanistic Investigations: (i) Structural Analysis of ZF2–4 Fragments and Their Reactivity toward Aβ Aggregation and Cytotoxicity

Following systematic, multiscale validation spanning molecular, cellular, and organismal levels, we next sought to establish mechanistic principles underlying ZF2–4‐driven amyloid assembly control, thereby providing guidelines for ZF‐inspired protein engineering. MD simulations of the ZF2–4–Aβ_40_ heterodimer revealed domain‐specific contributions to binding (Figure 2a). The CSAs between the ZF2 and ZF4 domains and Aβ_40_ were 1,049 and 1,198 Å^2^, respectively, whereas the ZF3–Aβ_40_ interface was substantially smaller (284 Å^2^). These results indicate that intermolecular contacts between ZF2–4 and Aβ_40_ are predominantly mediated by the ZF2 and ZF4 modules, implying them as key structural determinants governing Aβ_40_ binding and the subsequent modulatory activity of ZF2–4 toward Aβ_40_ amyloidogenesis.

Importantly, ZF2–4 retained intact Zn(II)‐coordinated ZF motifs upon binding to Aβ_40_, consistent with the monomeric structural model. In contrast, a β‐hairpin motif within the ZF2 domain, spanning amino acid residues Leu5–Cys7 and Gly11–Phe14, was disrupted upon heterodimer formation (Figure 2e and Video S1; Figures S1 and S2). The loss of this β‐hairpin, together with concomitant extensive intermolecular contacts between ZF2 and Aβ_40_, likely contributes to the modulatory reactivity of ZF2–4 toward Aβ biomolecular assembly (vide supra).

To further clarify the structural determinant responsible for these interactions, we examined the AlphaFold‐predicted structure of ZF2–4 (Figure S2) to identify domain‐specific structural features. The predicted ZF2–4 architecture comprises a combination of random coils, α‐helices, and β‐hairpins localized primarily within the ZF2 and ZF4 modules. In the case of the ZF3 domain, discernible β‐strand elements were absent, suggesting structural heterogeneity among the ZF modules and motivating a systematic investigation of how these structural differences influence functional reactivity.

To this end, we prepared individual ZF2–4 fragments (ZFx′; Figure 6a), namely ZF2′, ZF3′, and ZF4′, each containing a single ZF module derived from ZF2–4. These fragments enabled us to dissect the structural determinant governing Aβ recognition and the subsequent modulation of amyloidogenesis. At the primary sequence level, ZF2′, ZF3′, and ZF4′ all retain the conserved Cys_2_His_2_ ZF motif (C–X_2_–C–X_12_–H–X_3_–H) and share similar physicochemical characteristics, including amphiphilic molecular surfaces and basic pIs that favor interactions with Aβ under physiological conditions (Figure S22). In contrast, CD spectroscopy revealed marked differences in their secondary structures (Figure 6b). Whereas ZF2′ and ZF4′ displayed pronounced β‐strand signatures, ZF3′ exhibited a weaker signal at the characteristic local minimum near 225 nm, indicating a lower β‐strand content. Quantitative analysis estimated β‐strand populations of 32.9 (± 4.4)% for ZF2′, 7.8 (± 5.1)% for ZF3′, and 19.8 (± 4.2)% for ZF4′. Similar to these spectroscopic observations, AlphaFold‐predicted structures showed that ZF2′ and ZF4′ adopt anti‐parallel β‐hairpin architectures comprising two β‐strands connected by a short loop, while ZF3′ lacks a β‐hairpin motif altogether (Figure 6c).

**FIGURE 6:**
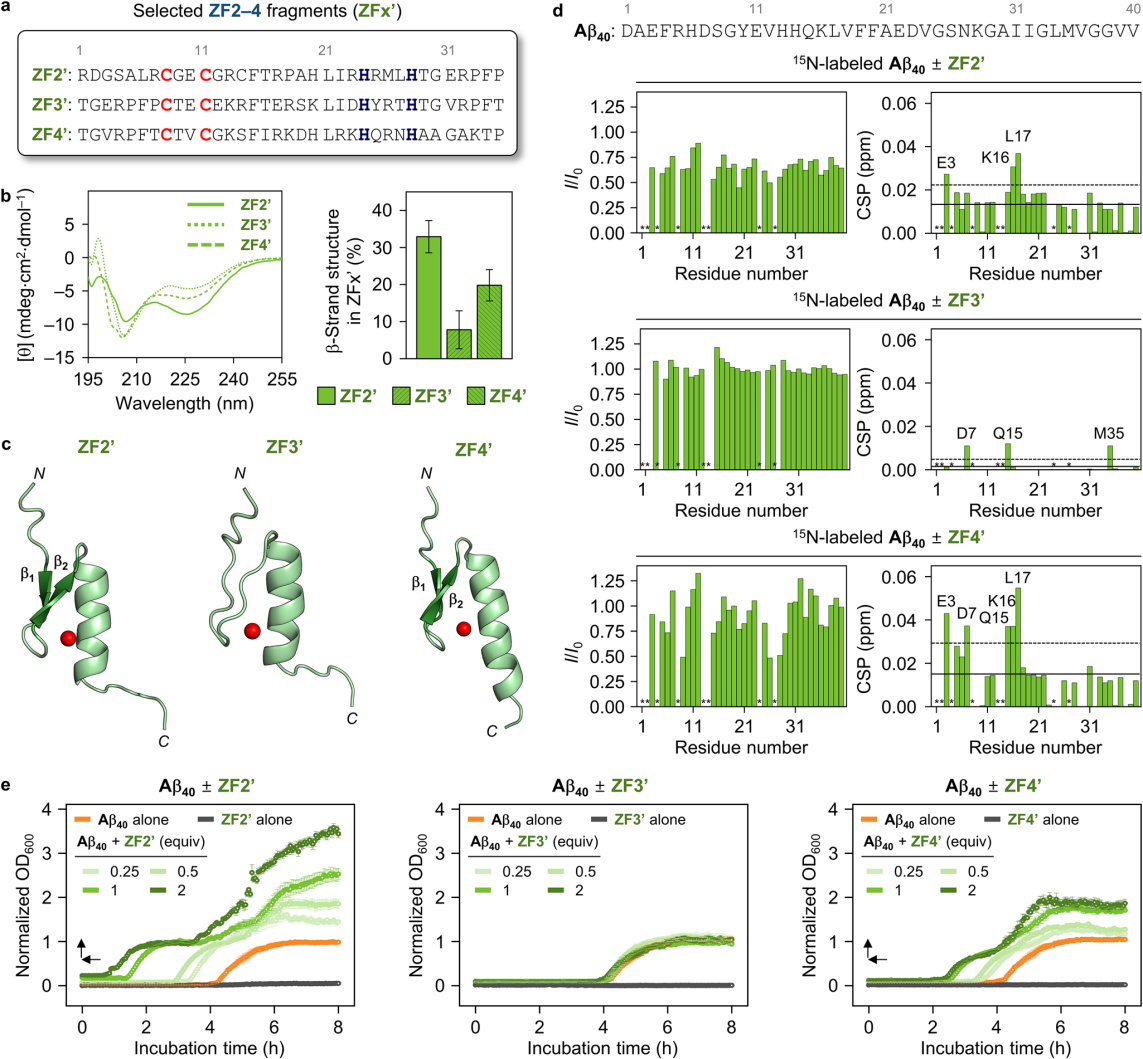
Structures of ZF2–4 fragments and their reactivity toward Aβ amyloidogenesis. (a) Domain organization of ZF2–4 and its individual fragments containing a single ZF module (ZF2′, ZF3′, or ZF4′). The cysteine and histidine residues involved in Zn(II) coordination are highlighted in bold red and navy, respectively. (b) Secondary structures of ZF2–4 fragments [ZFx′; Zn(II)‐bound form] analyzed by CD spectroscopy. The β‐strand contents of ZF2′, ZF3′, and ZF4′ were quantified from the CD spectra using BeStSel [64]. Conditions: [apo‐ZFx′] = 100 µM; [Zn(II)] = 90 µM; 20 mM HEPES, pH 7.4, 150 mM NaF, 1 mM DTT. It should be noted that 0.9 equiv of Zn(II) was added to apo‐ZFx′ and incubated for 3 min to minimize free Zn(II) in solution. (c) Structural models of ZF2′, ZF3′, and ZF4′ predicted by AlphaFold2 [35, 36], followed by docking of Zn(II). It should be noted that AlphaFold2 predicts ZF structures based on databases containing Zn(II)‐bound forms. (d) Peak intensity ratios and CSPs of ^15^N‐labeled Aβ_40_ upon incubation with ZFx′. The mean CSPs and mean + one standard deviation are indicated by the solid and dashed lines, respectively. The corresponding 2D NMR spectra are shown in Figure S23. Conditions: [^15^N‐labeled Aβ_40_] = 20 µM; [ZFx′] = 200 µM; 20 mM HEPES, pH 7.4, 1 mM DTT, 10% v/v D_2_O; 10°C. Unresolved peaks are marked with asterisks. (e) Aggregation kinetics of Aβ_40_ incubated with different concentrations of ZF2′, ZF3′, or ZF4′ [Zn(II)‐bound form] monitored by the turbidity assay. Conditions: [Aβ_40_] = 100 µM; [apo‐ZFx′] = 25, 50, 100, and 200 µM; [Zn(II)] = 22.5, 45, 90, and 180 µM; 20 mM HEPES, pH 7.4, 150 mM NaCl, 1 mM DTT; 37°C; quiescence; λ_abs_ = 600 nm. It should be noted that 0.9 equiv of Zn(II) was added to apo‐ZFx′ and incubated for 3 min to minimize free Zn(II) in solution. Data are presented as mean ± s.e.m. of three independent experiments.

Building on our structural analyses, we next investigated whether conformational differences among ZFx′ fragments are associated with distinct Aβ interaction and aggregation‐modulating reactivities. 2D ^1^H–^15^N HMQC NMR experiments employing uniformly ^15^N‐labeled Aβ_40_ revealed different interaction profiles for the individual ZF domains (Figures 6d and S23). In contrast to ZF2′ and ZF4′, ZF3′ induced negligible CSPs and changes in peak intensity ratios, consistent with its minimal contribution to Aβ_40_ binding predicted by the CSA analyses (Figure 2a; vide supra). Both ZF2′ and ZF4′ produced residue‐specific perturbations spanning the *N*‐terminal and central regions of monomeric Aβ_40_. To structurally interpret these observations, additional molecular docking simulations positioned the perturbed Aβ_40_ residues in proximity to Arg7, Arg13, and Arg32 of ZF2′ and Lys13, Ser14, Ile16, and Arg17 of ZF4′ through intermolecular hydrogen‐bonding interactions, all of which reside within the characteristic α–β–β folds of the corresponding ZF domains (Figure S24). Notably, both ZF2′ and ZF4′ directly engaged Gln15, Lys16, and/or Leu17, amino acid residues located within or immediately adjacent to the fibril‐forming self‐recognition sites of Aβ_40_ (Figure 1a) [23, 25, 26, 27]. Collectively, these structural and biophysical analyses suggest that the β‐hairpin‐containing ZF domains preferentially recognize amino acid residues of Aβ critical for intermolecular self‐recognition, thereby providing a structural basis for their ability to alter Aβ aggregation behavior.

Functionally, turbidity assays showed that ZF2′ and ZF4′ induced a biphasic growth profile during Aβ_40_ aggregation, whereas ZF3′ displayed negligible reactivity (Figure 6e). In the presence of equimolar ZF2′ or ZF4′, the absorbance intensity of Aβ_40_ increased by 2.8 (± 0.1)‐ and 1.7 (± 0.1)‐fold, respectively, relative to Aβ_40_ alone, accompanied by accelerated aggregation kinetics (Table S1). Increasing the concentrations of ZF2′ or ZF4′ further enhanced aggregation dynamics, as reflected by higher maximum absorbance values and faster kinetic parameters.

Morphological analyses showed that Aβ_40_ assemblies generated with sub‐equimolar amounts of ZF2′ or ZF4′ consisted of mixed populations of fragmented fibrils and less structured assemblies (Figure S25), which have been reported to exhibit reduced cytotoxicity [51, 52, 53]. At higher concentrations of ZF2′ or ZF4′, Aβ_40_ predominantly produced large, non‐fibrillar unstructured aggregates even after 8 h of incubation, whereas ZF3′ produced no discernible changes in Aβ_40_ aggregate morphology (Figure S25). Consistent with these aggregation profiles, treatments with ZF2′ and ZF4′ improved cell viability by 22.0 (± 3.3) – 32.3 (± 2.2)% and 13.8 (± 2.8) – 21.3 (± 3.4)%, respectively, in a concentration‐dependent manner relative to Aβ_40_ alone, whereas ZF3′ failed to alleviate Aβ_40_‐induced cytotoxicity (Figure S26). These overall findings confirm that the modulatory activity of ZF2–4 toward Aβ_40_ is primarily driven by the ZF2′ and ZF4′ modules. The presence of β‐hairpin‐containing ZF domains therefore emerges as a critical structural feature for controlling Aβ amyloidogenesis and cytotoxicity.

### 2.6 Mechanistic Investigations: (ii) Sequence‐Based Reactivity Analyses Toward Aβ Amyloidogenesis

To delineate the role of β‐strand motifs in controlling Aβ amyloidogenesis, we systematically engineered two series of ZF2′ variants (Mx, 44 variants in total; Figure S27) designed to either preserve or disrupt the β‐hairpin structure of the parent ZF2′ scaffold. These variants were generated through targeted substitutions within the β_1_ and β_2_ regions to change β‐strand formation. Structural screening based on AlphaFold2 predictions [35, 36], revealed clear sequence–structure relationships. The Mx variants consistently adopted a β‐hairpin conformation when the β_2_ region initiated with a Gly residue, regardless of the β_1_ sequence. Conversely, variants containing the Glu12 residue in the β_2_ position generally lacked β‐strand elements, with the exceptions of M32, M38, and M41, which share the conserved Phe6‐Thr7‐Cys8 motif. Based on these structural trends (Figures 7a,b and S27), we selected representative ZF2′ variants with the relatively abundant β‐strand character, such as M28 and M32, and compared their reactivity toward Aβ_40_ with that of their corresponding parent variants (M13 and M30), which lack β‐strand conformations. This design enabled a direct assessment of how sequence‐encoded β‐hairpin motifs govern the ability of ZF‐derived fragments to modulate Aβ amyloidogenesis.

**FIGURE 7:**
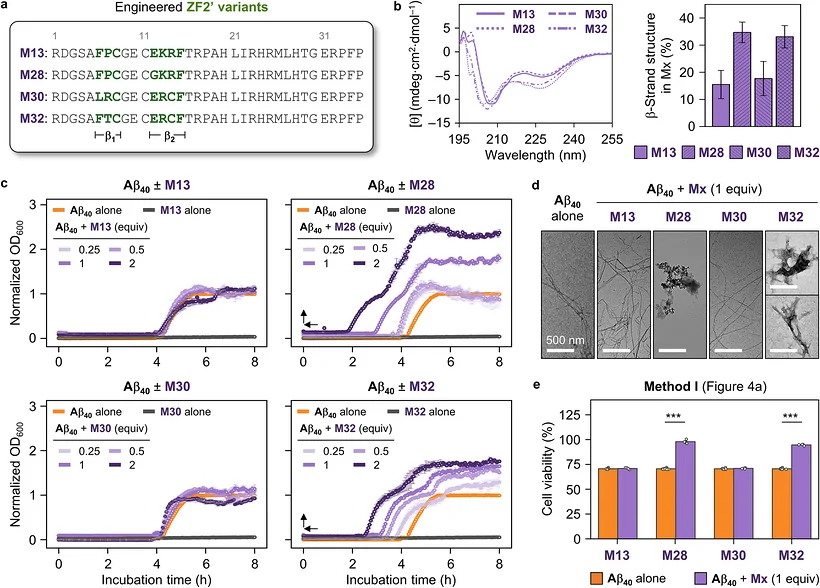
Impact of ZF2′ variants on Aβ_40_ aggregation and cytotoxicity. (a) Amino acid sequences of selected ZF2′ variants (M13, M28, M30, and M32). Amino acid residues located within the β‐hairpin regions are highlighted in bold and green. (b) Secondary structures of M13, M28, M30, and M32 [Zn(II)‐bound form] analyzed by CD spectroscopy. β‐strand contents were estimated from the CD spectra using BeStSel [64]. Conditions: [apo‐Mx] = 100 µM; [Zn(II)] = 90 µM; 20 mM HEPES, pH 7.4, 150 mM NaF, 1 mM DTT. (c) Aggregation kinetics of Aβ_40_ incubated with and without selected ZF2′ variants [Mx; Zn(II)‐bound form] monitored by the turbidity assay. Conditions: [Aβ_40_] = 100 µM; [apo‐Mx] = 25, 50, 100, and 200 µM; [Zn(II)] = 22.5, 45, 90, and 180 µM; 20 mM HEPES, pH 7.4, 150 mM NaCl, 1 mM DTT; 37°C; quiescence; λ_abs_ = 600 nm. Data are presented as mean ± s.e.m. of three independent experiments. (d) Morphologies of the resultant Aβ_40_ aggregates formed in the absence and presence of Mx visualized by TEM after 8 h incubation. Conditions: [Aβ_40_] = 100 µM; [apo‐Mx] = 100 µM; [Zn(II)] = 90 µM; 20 mM HEPES, pH 7.4, 150 mM NaCl, 1 mM DTT; 37°C; quiescence. Scale bar = 500 nm. (e) Cell viability following treatment with Aβ_40_ incubated with and without Mx determined by the MTT assay. Cell survival was calculated relative to that with an equivalent amount of the buffered solution. Conditions: [Aβ_40_] = 10 µM; [apo‐Mx] = 10 µM; [Zn(II)] = 9 µM; 20 mM HEPES, pH 7.4, 150 mM NaCl; 8 h pre‐ and 48 h post‐incubation; 37°C; quiescence; SH‐SY5Y cells that were non‐differentiated. ***P < 0.001; *n* = 3; Student's *t*‐test. Data are presented as mean ± s.e.m.

As presented in Figure 7c–e, Figures S28 and S29, the β‐hairpin‐containing variants M28 and M32 effectively redirected Aβ_40_ aggregation toward non‐fibrillar pathways, leading to the formation of less cytotoxic aggregates. In contrast, this modulatory behavior was not observed for M13 and M30, which lack β‐strand motifs, despite sharing the same ZF2′ framework. Notably, M28, M32, and ZF2′ originate from a common structural scaffold but differ in their amino acid compositions within the β_1_ and β_2_ regions, resulting in distinct aggregation dynamics. These differences are reflected by distinguishable kinetic parameters (Table S1) and differential abilities to attenuate Aβ_40_‐associated cytotoxicity (Figures S26 and S29).

Together, these findings indicate that not only the presence but also the specific sequence composition of the β‐hairpin motif plays a critical role in redirecting the amyloidogenic pathway of Aβ toward nontoxic aggregation. Our sequence–structure–function analyses therefore identify the structural determinants underlying ZF‐mediated modulation of Aβ assembly and toxicity. In this context, despite sharing the same repetitive amino acid pattern and broadly similar physicochemical properties with ZF2′ and ZF4′, the negligible Aβ interaction and aggregation‐modulating activity of ZF3′ is most consistent with its markedly reduced β‐strand character. Conversely, the ZF2 and ZF4 domains, which adopt β‐hairpin architectures, emerge as the primary structural determinants governing the anti‐amyloidogenic activity of ZF2–4 against Aβ. These findings establish rational design principles for engineering ZF‐inspired biomaterials with programmable anti‐amyloid functions.

## 3 Conclusions

ZF proteins are widely recognized for their engineerable capacity to regulate transcription and translation via nucleic acid recognition. In contrast, their potential to operate through direct protein–protein interactions has remained largely unexplored, despite the structural versatility provided by Zn(II)‐centered coordination chemistry. In this work, we repurpose modular ZF proteins, traditionally associated with genetic regulation, as chemically programmable biomolecular materials capable of regulating pathological protein phase transitions and assembly.

We demonstrate that the bioinspired ZF2–4 protein, which possesses distinctive structural and physicochemical features, including tandemly arranged ZF modules, a high isoelectric point, and an amphiphilic multivalent surface, forms hetero‐complexes with Aβ through direct protein–protein interactions. Through these interactions, ZF2–4 alters Aβ LLPS and aggregation behavior, redirecting β‐sheet‐rich fibrillization toward nontoxic, non‐fibrillar unstructured assemblies while actively disassembling preformed fibrils into less toxic species. To establish translational potential, we engineered a BBB‐penetrant variant (ZF2–4–P9), which reduced Aβ deposition, attenuated neuroinflammation, and restored recognition memory upon administration to AD APP/PS1 Tg mice. Across multiple experimental contexts, including in vitro aggregation studies, cellular toxicity measurements, and histopathological and behavioral analyses in AD Tg mice, ZF2–4 therefore emerges as a bioinspired platform for modulating pathogenic biomolecular phase transitions and toxicity through programmable protein–protein interactions. Mechanistic sequence–structure–function analyses further revealed that, in contrast to ZF3, the ZF2 and ZF4 domains serve as the primary structural determinants of Aβ binding and aggregation modulation particularly through their β‐hairpin motifs. These findings establish sequence‐encoded β‐hairpin motifs within ZF modules as modular structural elements that govern Aβ recognition and provide rational design principles for engineering ZF‐inspired anti‐amyloid biomaterials.

Collectively, this study provides the first demonstration that ZF proteins can directly and functionally interact with amyloidogenic peptides to regulate their phase behavior and assembly. By identifying modular ZF repeats as programmable metalloprotein scaffolds for controlling pathological protein aggregation, our work expands the functional scope of ZF proteins beyond genetic regulation and introduces a new strategy for manipulating biomolecular phase transitions. More broadly, these findings establish a structural and rational design framework for engineering ZF‐inspired metalloprotein materials and therapeutics, opening new opportunities at the interface of bioinorganic chemistry, biomolecular materials, and translational approaches to neurodegenerative diseases.

## 4 Methods

All chemical reagents were purchased from commercial suppliers and used as received unless otherwise stated. ZF2–4, ^15^N‐labeled ZF2–4, and ZF2–4–P9 were recombinantly expressed in *E. coli* and purified. Aβ_40_ and Aβ_42_ were obtained from Peptide Institute, Inc. (Osaka, Japan) that were purified by high‐performance liquid chromatography using YMC Pack ODS‐A (YMC CO., LTD., Kyoto, Japan) and Agilent ZORBAX 300SB‐C18 columns (Agilent, Santa Clara, CA, USA). ^HF488^Aβ_40_ and uniformly ^15^N‐labeled Aβ_40_ were purchased from AnaSpec (Fremont, CA, USA) and rPeptide Inc. (Bogart, GA, USA), respectively. ZF2–4 fragments and ZF2′ variants (ZF2′, ZF3′, ZF4′, M13, M28, M30, and M32) were obtained from Anygen (Gwangju, Republic of Korea). HEPES [2‐(4‐(2‐hydroxyethyl)piperazin‐1‐yl)ethanesulfonic acid] and MOPS [3‐(*N*‐morpholino)propanesulfonic acid] were purchased from Sigma‐Aldrich (St. Louis, MO, USA). The buffered solution was prepared in doubly distilled water [ddH_2_O; a Milli‐Q Direct 16 system (18.2 MΩ·cm; Merck KGaA, Darmstadt, Germany)]. Trace metal contamination was removed from all solutions used for experiments by treating with Chelex (Sigma‐Aldrich) overnight. The concentrations of peptides were determined by an Agilent 8453 ultraviolet−visible spectrophotometer (Agilent). The concentration of zinc in prepared ZF2–4 was measured by an Agilent ICP–MS 7700S [Agilent; KAIST Analysis Center for Research Advancement (KARA), Daejeon, Republic of Korea]. The hydrodynamic radius was determined by a Malvern Zetasizer Nano ZSP DLS system (Malvern Panalytical Ltd., Worcestershire, UK). 2D ^1^H–^15^N HMQC or HSQC NMR spectroscopy was conducted by an AVANCE II‐800 MHz NMR spectrometer [Bruker BioSpin, Rheinstetten, Germany; Korea Basic Science Institute (KBSI), Ochang, Republic of Korea] equipped with a cryogenic probe. ITC measurements were performed by a PEAQ‐ITC instrument (Malvern Panalytical Ltd.). The fluorescence spectra were recorded on a FP‐8300 spectrofluorometer (JASCO Inc., Tokyo, Japan). The absorbance and fluorescence values were measured by a SpectraMax M5 microplate reader (Molecular Devices, Sunnyvale, CA, USA) and a HORIBA QuantaMaster QM 400 spectrophotometer (HORIBA, Kyoto, Japan; KARA). Images gained through gel/Western blot were visualized by a ChemiDoc MP imaging system (Bio‐Rad, Hercules, CA, USA). Morphologies of Aβ aggregates produced from aggregation experiments were taken on a Tecnai F20 transmission electron microscope (FEI Company, Eindhoven, Netherlands; KARA). The secondary structures of samples were analyzed by a JASCO‐815 150‐L CD spectropolarimeter (JASCO Inc.). Live‐cell microinjection experiments were performed by the combined system composed of an InjectMan 4 micromanipulator (Eppendorf, Hamburg, Germany) and a Femtojet 4i microinjector (Eppendorf). Fluorescence analysis of live cells was conducted by an EVOS FL fluorescence microscope (Advanced Microscopy Group, Bothell, WA, USA). Confocal microscopic images were taken by a Zeiss LSM 980 (Carl ZEISS, Oberkochen, Germany; KARA). Mass spectrometric analyses were conducted using a Waters Xevo TQ‐S micro Quadrupole mass spectrometer equipped with an ACQUITY Sample Manager FTN‐I and Binary Solvent Manager (Milford, MA, USA; Center for University‐Wide Research Facilities, Jeonbuk National University, Jeonju, Republic of Korea). Detailed experimental procedures are described in the Supporting Information.

## Author Contributions

**Seongmin Park**: conceptualization, investigation, formal analysis, writing – original draft, writing – review and editing. **Yunha Hwang**: investigation, formal analysis, writing – review and editing. **Jimin Kwak**: investigation, formal analysis, writing – original draft, writing – review and editing. **Yuxi Lin**: investigation, formal analysis. **Hye‐Yeon Park**: investigation, formal analysis. **Eunju Nam**: investigation, formal analysis. **Jiyeon Han**: investigation, formal analysis. **Soyeon Park**: investigation, formal analysis. **Hyunyong Kim**: investigation, formal analysis. **Ju‐Eun Kim**: investigation, formal analysis. **Hyun Goo Kang**: investigation, formal analysis. **Jiyong Park**: investigation, formal analysis, writing – review and editing, funding acquisition, writing – original draft. **Young‐Ho Lee**: investigation, formal analysis, funding acquisition, writing – review and editing. **Kyoung‐Shim Kim**: investigation, formal analysis, writing – review and editing. **Chul‐Ho Lee**: investigation, formal analysis, writing – review and editing, funding acquisition. **Seung Jae Lee**: conceptualization, investigation, formal analysis, writing – review and editing, funding acquisition. **Mi Hee Lim**: conceptualization, funding acquisition, project administration, writing – original draft, writing – review and editing, supervision.

## Funding

This work was supported by the National Research Foundation of Korea (NRF) grant funded by the Korean government {Creative Research Initiative [RS‐2022‐NR070709 (M.H.L.)], Global Science Research Center Program [RS‐2024‐00411134 (M.H.L.)], RS‐2025‐23292974 (S.J.L.), RS‐2021‐NR057690, RS‐2022‐NR069719, and RS‐2025‐25444385 (Y.‐H.L.), RS‐2024‐00407337 (J.K.)}, the KRIBB Research Initiative Program [KGM1312612 (C.‐H.L.)], the Korea Institute of Marine Science & Technology Promotion funded by the Ministry of Oceans and Fisheries [RS‐2026‐25541458 and RS‐2026‐25508103 (Y.‐H.L.)]; the KBSI funds [C623300, C623100, C612120, and C623500 (Y.‐H.L.)], the Institute for Basic Science [IBS‐R010‐A1 (J.P.)].

## Ethics Approval Statement

All animal experiments were approved by the Institutional Animal Care and Use Committee of KRIBB and were performed in accordance with the institutional guidelines at KRIBB (approval number, KRIBB‐AEC‐25182 and KRIBB‐AEC‐26267).

## Conflicts of Interest

The authors declare no conflicts of interest.

## Supporting information




**Supporting File 1**: advs77886‐sup‐0001‐SuppMat.pdf.

**Supporting File 2**: advs77886‐sup‐0002‐VideoS1.mp4.

**Supporting File 3**: advs77886‐sup‐0003‐VideoCaption.docx.

## Data Availability

The data that support the findings of this study are available from the corresponding author upon reasonable request.
