# Supplementary material for: Zinc Fingers as Programmable Metalloprotein Scaffolds for Altering Pathogenic Biomolecular Phase Transitions
Source: Adv Sci (Weinh). 2026 Sep 30:e77886. Online ahead of print. doi: 10.1002/advs.77886 (PMC13624870; doi:10.1002/advs.77886)
Supplement: Supplementary file 1 [file ADVS-9999-e77886-s003.pdf]

Supporting Information

Zinc Fingers as Programmable Metalloprotein Scaffolds for Altering Pathogenic Biomolecular Phase Transitions

Seongmin Park, Yunha Hwang, Jimin Kwak, Yuxi Lin, Hye-Yeon Park, Eunju Nam, Jiyeon Han, Soyeon Park, Hyunyong Kim, Ju-Eun Kim, Hyun Goo Kang, Jiyong Park, Young-Ho Lee,* Kyoung-Shim Kim,* Chul-Ho Lee,* Seung Jae Lee,* and Mi Hee Lim**

Supporting Methods

Syntheses of ZF2–4 and ZF2–4–P9

Synthesis of ^{15}N-Labeled Recombinant ZF2–4

Preparation of Amyloid-β (Aβ), ZF2–4 Fragments, and ZF2' Variants

Preparation of HiLyte^{TM} Fluor 555- and 647-Labeled ZF2–4 (^{HF555}ZF2–4 and ^{HF647}ZF2–4)

Prediction of Protein Structures by AlphaFold2 and AlphaFold3

Heterodimer Models

Molecular Dynamics (MD) Simulations of ZF2–4–Aβ_{40} Heterodimer Models

Selection of Relatively Stable ZF2–4–Aβ_{40} Heterodimer Models

Molecular Mechanics/Poisson-Boltzmann Surface Area (MM/PBSA) Analysis of ZF2–4–Aβ_{40}

Hydrodynamic Radius Measurements

Two-Dimensional (2D) ^{1}H–^{15}N Nuclear Magnetic Resonance (NMR) Experiments

Isothermal Titration Calorimetry (ITC)

Fluorescence-Quenching Measurements

Job Plot

Liquid–Liquid Phase Separation (LLPS) Studies

Turbidity Assay

Gel Electrophoresis with Western Blotting (Gel/Western Blot)

Transmission Electron Microscopy (TEM)

Circular Dichroism (CD) Spectroscopy

Thioflavin-T (ThT) Fluorescence Assay

8-Anilino-1-Naphthalenesulfonic Acid (ANS) Fluorescence Assay

Cell Culture and Cell Viability Measurements

Cell Culture and Microinjection Studies

Cellular and *Ex Vivo* Co-Localization of ZF2–4 with Aβ
Live-Cell Imaging Experiments
Parallel Artificial Membrane Permeability Assay Adapted for the Blood-Brain Barrier (PAMPA–BBB)
In Vivo Biodistribution Studies
Animals and Administration of ZF2–4–P9
Histochemical Analysis
Immunoblot Analysis of Aβ
Real-Time Polymerase Chain Reaction (RT–PCR)
Cognitive Behavior Evaluation
Blood Chemistry
Statistical Analysis

Supporting Table and Figures

Table S1 Kinetic parameters obtained from the turbidity assay
Table S2 Concentrations of ZF2–4–P9 in the brain, liver, kidney, and plasma 10 min after the intraperitoneal (*i.p.*) administration of ZF2–4–P9 (3 mg/kg) in mice
Table S3 Sequences of primer pairs

Figure S1 Structures of ZF2–4 and $A\beta_{40}$ used for MD simulations
Figure S2 Structural homology of ZF2–4 predicted by AlphaFold2 and AlphaFold3
Figure S3 Docking models of ZF2–4–$A\beta_{40}$ heterodimers
Figure S4 Computed root-mean-squared deviation (RMSD) values of ZF2–4–$A\beta_{40}$ heterodimers
Figure S5 RMSD profiles from the extended MD simulations of selected ZF2–4–$A\beta_{40}$ heterodimer models
Figure S6 Characterization of ZF2–4
Figure S7 Individual 2D NMR spectra of ^{15}N-labeled $A\beta_{40}$ alone and in the presence of ZF2–4
Figure S8 2D NMR analyses of ^{15}N-labeled ZF2–4 upon treatment of $A\beta_{40}$
Figure S9 Binding properties of ZF2–4 toward $A\beta_{40}$ measured by fluorescence-quenching experiments
Figure S10 Size distributions of ZF2–4 and $A\beta_{40}$ monitored by tricine–polyacrylamide gel electrophoresis (tricine–PAGE) experiments

Figure S11 Original tricine–PAGE data from Figure 3f and S10

Figure S12 Change in the secondary structure of Aβ_{40} upon aggregation with ZF2–4 analyzed by CD spectroscopy

Figure S13 Impact of ZF2–4 on the aggregation of Aβ_{42}

Figure S14 Effect of ZF2–4 on preformed Aβ_{40} aggregates

Figure S15 Preparation and characterization of ZF2–4–P9

Figure S16 Effects of ZF2–4–P9 on the body weight and locomotor activity in amyloid precursor protein/presenilin-1 (APP/PS1) transgenic (Tg) mice

Figure S17 Influence of ZF2–4–P9 administration on the molecular weight distribution of Aβ species in brain homogenates from APP/PS1 Tg mice

Figure S18 Representative images visualized by immunostaining with anti-Iba-1 and anti-GFAP antibodies

Figure S19 Quantitative analysis of the spatial association between Aβ plaques and microglia in the parietal cortices and hippocampi of Veh-added and ZF2–4–P9-treated APP/PS1 Tg mice

Figure S20 mRNA expression levels of inflammatory cytokines in the microglia isolated from the cortices following treatment with Veh or ZF2–4–P9 in WT and APP/PS1 Tg mice

Figure S21 Blood chemistry analysis for the levels of aspartate aminotransferase (AST), alkaline phosphatase (ALP), albumin, Creatinine (CREA), alanine aminotransferase (ALT), total bilirubin (T-Bin), blood urea nitrogen (BUN), and creatine kinase (CK)

Figure S22 Physicochemical properties of ZF2', ZF3', and ZF4'

Figure S23 Individual 2D NMR spectra of ^{15}N-labeled Aβ_{40} with and without ZFx'

Figure S24 Structural analysis of heterodimeric ZF2'–Aβ_{40} and ZF4'–Aβ_{40} complexes obtained from molecular docking simulations

Figure S25 Morphologies of the resultant Aβ_{40} aggregates with and without ZFx' visualized by TEM

Figure S26 Influence of ZFx' on the cytotoxicity of Aβ_{40}

Figure S27 Amino acid sequences and AlphaFold2-predicted structures of Mx

Figure S28 Morphologies of the resultant aggregates by incubation of Aβ_{40} with and without Mx for 8 h monitored by TEM

Figure S29 Influence of Mx on the cytotoxicity of Aβ_{40}

Supporting References

Supporting Methods

Syntheses of ZF2–4 and ZF2–4–P9. ZF2–4 and ZF2–4–P9 were recombinantly expressed in *Escherichia coli* (*E. coli*) and purified.[1] The proteins were expressed in the *E. coli* HIT (DE3) cells that were transformed with the pET15b vector (Amersham, Buckinghamshire, UK) encoding ZF2–4 or ZF2–4–P9. The competent cells were inoculated into the ampicillin-containing lysogeny broth (200 mL) at 37 °C with constant agitation (200 rpm) for 12–16 h. After being cultured, protein expression was induced by isopropyl-β-D-1-thiogalactopyranoside (IPTG, 0.1 mM) at 25 °C for 6 h. The final cell pellets were obtained with centrifugation at 11,355 × *g* at 4 °C for 20 min and then lysed in 25 mM MOPS [3-(*N*-morpholino)propanesulfonic acid], pH 7.4, 50 mM NaCl, 5 mM $MgCl_2$, 1 mM DTT (dithiothreitol), 0.01 µL/mL DNase I, 0.002 mg/mL phenylmethylsulfonyl fluoride using a sonicator (Sonics & Materials, Inc., Newtown, CT, USA) for 40 min (15 s on and 45 s off). The whole cell lysate was centrifuged at 28,306 × *g* at 4 °C and filtered using a 0.22 µm membrane syringe filter to obtain the supernatant. The clear supernatant was then applied to a sulphopropyl-sepharose column, washed with the activating buffer (25 mM MOPS, pH 7.4, 50 mM NaCl, 1 mM DTT, 5% glycerol), and purified with the buffer (25 mM MOPS, pH 7.4, 1 mM DTT, 5% glycerol) containing gradient NaCl (50–1,000 mM). The fractions housing ZF2–4 or ZF2–4–P9 were centrifuged using a 3 kDa cut-off membrane filter (Merck Millipore, Burlington, MA, USA) at 4 °C and 2,095 × *g*. The concentrated ZF2–4 or ZF2–4–P9 fractions were re-applied to a Superdex 75 column, washed with the activating buffer, and filtered using a 3 kDa cut-off membrane filter at 4 °C and 2,095 × *g*. The final pure sample was concentrated, aliquoted, and stored at –80 °C until used. The fractions from each purification step were monitored by 15% sodium dodecyl sulfate–polyacrylamide gel electrophoresis (SDS–PAGE) and Coomassie brilliant blue staining. The concentrations of the protein and zinc bound to the protein were determined by electronic absorption spectroscopy (ε_{280} = 1,865 $M^{-1}cm^{-1}$) and inductively coupled plasma-mass (ICP–MS) spectrometry.

Synthesis of ^{15}N-Labeled Recombinant ZF2–4. The expression and purification of ^{15}N-labeled ZF2–4 were identical to those of ZF2–4, except for the cultivated system in the ^{15}N-labeled M9 mineral medium. The ^{15}N-labeled M9 mineral medium was prepared by dissolving the ^{15}N-labeled M9 salt solution (10x), 0.4% D-(+)-glucose, 1 mM $MgSO_4 \cdot 7H_2O$, 1 µg/mL biotin, 1 µg/mL thiamin·HCl, and 10 mL of the trace elements solution (100x) in ddH_2O. The ^{15}N-labeled M9 salt solution (10x) was dissolved in ddH_2O composed of 423 mM Na_2HPO_4, 220 mM KH_2PO_4, 85.6 mM NaCl, and 91.8 mM $^{15}NH_4Cl$, while the trace elements solution

(100x) was dissolved in ddH_2O containing 13.4 mM EDTA, 3.1 mM $FeCl_3 \cdot 6H_2O$, 0.62 mM $ZnCl_2$, 76 μM $CuCl_2 \cdot 2H_2O$, 42 μM $CoCl_2 \cdot 6H_2O$, 162 μM H_3BO_3, and 8.1 μM $MnCl_2 \cdot 4H_2O$. The purified ^{15}N-labeled ZF2–4 was stored at –80 °C until used.

Preparation of Aβ, ZF2–4 Fragments, and ZF2' Variants. Aβ was dissolved in NH_4OH (1% w/w, aq; Sigma-Aldrich, St. Louis, MO, USA). The resultant solutions were aliquoted, lyophilized overnight, and stored at –80 °C. A stock solution of Aβ was prepared by dissolving the lyophilized peptide with NH_4OH (1% w/w, aq; 10 μL) followed by addition of ddH_2O, as previously reported.[2] In the case of ZF proteins, the stock solutions of ZF2–4 fragments and ZF2' variants were prepared by dissolving the lyophilized peptide in ddH_2O. The concentration of the Aβ solution was determined by measuring the absorbance at 280 nm (for $A\beta_{40}$, ε_{280} = 1,450 $M^{-1}cm^{-1}$; for $A\beta_{42}$, ε_{280} = 1,490 $M^{-1}cm^{-1}$). As the extinction coefficients of ZF2–4 fragments and ZF2' variants are not known, their concentrations were determined based on theoretical extinction coefficients (for ZF2' and M30, ε_{205} = 140,670 $M^{-1}cm^{-1}$; for ZF3', ε_{280} = 1,490 $M^{-1}cm^{-1}$; for ZF4', ε_{205} = 134,900 $M^{-1}cm^{-1}$; for M13 and M28, ε_{205} = 147,230 $M^{-1}cm^{-1}$; for M32, ε_{205} = 147,920 $M^{-1}cm^{-1}$) obtained from the protein-parameter calculator online tool.[3]

Preparation of ^{HF555}ZF2–4 and ^{HF647}ZF2–4. The labeling of ZF2–4 with HiLyte™ Fluor 555 or HiLyte™ Fluor 647 was carried out employing an NH_2-reactive HiLyte™ 555 or an NH_2-reactive HiLyte™ 647 labeling kit, respectively, following the manufacturer's protocol (AnaSpec, Fremont, CA, USA). The reaction mixture was prepared by adding the dye solution (9 mM) to 10 mg/mL ZF2–4 with the 10:1 dye-to-protein molar ratio. Under dark conditions, the mixture was shaken for 1 h at room temperature and, subsequently, loaded onto an activated desalting column containing the elution buffer. The ^{HF555}ZF2–4 and ^{HF647}ZF2–4 were then purified from the protein-unbound dye, collected, and stored at –20 °C until used. All experiments were performed in a N_2-filled glove box (Coy Laboratory Inc., Grass Lake, MI, USA).

Prediction of Protein Structures by AlphaFold2 and AlphaFold3. We used the AI-based computational method (AlphaFold2 and AlphaFold3)[4-6] to predict three-dimensional (3D) protein structures based on their amino acid sequences. Among multiple structural candidates, the best ranked model with the highest pLDDT (predicted local distance difference test) score was visualized using PyMOL software as a representative predicted model.

Heterodimer Models. We prepared the models of heterodimeric interfaces comprising Aβ_{40} and ZF2–4, ZF2', or ZF4' based on docking simulations. For the models of ZF2–4, ZF2', or ZF4', we used an AlphaFold2[4,5]-predicted 3D model with the highest pLDDT score. For the structural model of Aβ_{40}, the solution-phase NMR structure of Aβ_{40} monomer deposited in the protein data bank (PDB 2LFM[7]) was used as its initial conformation. Subsequently, docking simulations using the structural models of individual proteins were conducted using the ClusPro protein–protein docking server.[8] The models of Aβ_{40} docked with ZF2–4, ZF2', or ZF4' were visualized using PyMOL software. Among them, the twelve ZF2–4–Aβ_{40} and one ZF2'–Aβ_{40} or ZF4'–Aβ_{40} heterodimeric models, with relatively lower binding energy, were selected.

MD Simulations of ZF2–4–Aβ_{40} Heterodimer Models. To evaluate the stabilities of the ZF2–4–Aβ_{40} heterodimer models, we performed a series of MD simulations initiated from the results of docking simulations. For each docked model, we prepared two models with and without Zn(II) bound to ZF motifs. Then each dimer model was solvated in an octahedral box filled with TIP3P water molecules, while maintaining at least 20 Å of minimal distance from the surface of the protein to the solvation boundary. Chlorine anions were included to ensure the charge neutrality. AmberFF14SB force field was used to model the peptide.[9] The 12-6-4 Lennard-Jones (LJ) parameters were adopted to parameterize chlorine anions and Zn(II) cations.[10,11] It should be noted that the 12-6-4 LJ parameters were shown to reproduce the tetrahedral coordination environment of Zn(II)-binding sites better than the standard 12-6 LJ parameter.[12] For MD simulations, a 0.2 fs integration step was used with the Settle algorithm that constrains the bond distances between a heavy atom and attached hydrogens.[13] We applied the Particle Mesh Ewald (PME) algorithm to facilitate the computations of long-range electrostatic interactions.[14] The energy of each solvated model was minimized *via* 4,000 steps, while restraining the coordinates of all carbon atoms using harmonic potentials with the spring constant of 4.0 kcal/mol/Å^2. The temperature was elevated from 0 to 100 K in 2 ns, while restraining all carbon atoms with the same harmonic potentials for energy minimizations. The temperature was then increased from 100 to 300 K in 10 ns, while restraining all C_α atoms with harmonic potentials with the spring constant of 10 kcal/mol/Å^2. Additional 10 ns of equilibrations were performed at 300 K and 1 atm. We used Amber20 software suite for energy minimizations and MD simulations.[15]

Along with the equilibrated ZF2–4–Aβ_{40} interfaces, up to 1.0 μs constant temperature-pressure MD simulations were followed. Initially, MD simulations without Zn(II) were performed. We then selected the models without Zn(II) which preserved the dimer contacts

throughout 1.0 μs MD simulations. We then conducted 1.0 μs MD simulations for the selected dimer models in the presence of Zn(II) bound to ZF motifs. In this study, the selected models maintained the dimer interface in the presence of Zn(II), relative to that in the absence of Zn(II). Lastly, we continued the simulations up to 2.5 μs for two of the stable dimer models with Zn(II). Amber20 software suite augmented with a graphics processing unit was used for MD simulations.

Selection of Relatively Stable ZF2–4–Aβ_{40} Heterodimer Models. We compared the relative stabilities of MD simulations. First, we computed the RMSD relative to the initial docked model (Figure S3). The RMSD plot of MD trajectories in the absence of Zn(II) suggested that Models 1, 2, 4, 8, 9, 10, and 11 maintained the deviation lower than 8 Å from the initial docked model. Along with the seven selected models, we launched additional MD simulations with being bound to Zn(II). The simulations suggested that Models 8, 9, and 11 preserved the dimer interface in the presence of Zn(II). Lastly, we performed MD simulations of Models 8, 9, and 11 with Zn(II), in order to compare their stability in an extended time scale. For each model, five independent simulations were conducted, which was intended to rule out the dependence of sampled conformers to the initial condition. For Model 8, one independent trajectory preserved stable dimer interface up to 1.0 μs; however, when we extended the simulation further, the interface began to dissociate after 1.0 μs (Figure S4). In the case of Model 11, two out of five trajectories maintained the dimer interface up to 1.0 μs. Again, when the simulation of the stable runs was continued, we found one of the dimer interfaces was stable up to 2.5 μs, whereas the other run began to dissociate after 1.3 μs. On the contrary, all five independent runs of Model 9 did not maintain the dimer interface after 0.5 μs. Based on these findings, we concluded that Models 11 and 8 are the most and the second most stable heterodimer models, respectively.

MM/PBSA Analysis of ZF2–4–Aβ_{40}. We computed the binding affinity of the most stable dimer interface (Model 11) using the MM/PBSA method. The binding energy of the interface (ΔG_{bind}) comprises the gas-phase (ΔG_{gas}) and the solvation corrections (ΔG_{solv}) as below.

$$\Delta G_{\mathrm{bind}} = \Delta G_{\mathrm{gas}} + \Delta G_{\mathrm{solv}}$$

$$\Delta G_{\mathrm{bind}} = \Delta G_{\mathrm{gas}}(\mathrm{dimer}) - [\Delta G_{\mathrm{gas}}(\mathrm{ZF2–4}) + \Delta G_{\mathrm{gas}}(\mathrm{A\beta_{40}})]$$

$$\Delta G_{\text{solv}} = \Delta G_{\text{solv}}(\text{dimer}) - [\Delta G_{\text{solv}}(\text{ZF2–4}) + \Delta G_{\text{solv}}(\text{A}\beta_{40})]$$

The gas-phase energies of the heterodimer [ΔG_{gas}(dimer)] is the summation of covalent bonding, angle, dihedral, improper dihedral, Coulomb, van der Waals energies, and the translational and rotational entropic contributions of the molecule, which are computed at the MM level of the theory. The gas-phase energies of individual monomers ΔG_{gas}(ZF2–4) and ΔG_{gas}(Aβ_{40}) were averaged of the same MD trajectories by ignoring the other monomer. The solvation energies of the heterodimer and the individual monomers were computed using the PBSA method. We used *mbondi2* parameter set to define the solute atomic radii of the heterodimer and the monomers. The solvent ionic strength of 0.15 mol/L and the solute internal dielectric constant of 2.0 were used for the numerical solution of Poisson-Boltzmann equation. *MMPBSA.py* software implemented in Amber20 program suite was used for the computations of the binding free energies.[15]

Hydrodynamic Radius Measurements. The solutions of either Aβ_{40} (100 μM), ZF2–4 (100 μM), or both were prepared in 20 mM HEPES [2-[4-(2-hydroxyethyl)piperazin-1-yl]ethanesulfonic acid], pH 7.4, 1 mM DTT (1,4-dithiothreitol). To remove possible preformed aggregates, the prepared samples were passed through a filter (0.22 μm). Their hydrodynamic radius was then measured by dynamic light scattering (DLS) at room temperature. A laser operating at 633 nm was used as a light source. The scattered intensity was detected at an angle of 173° to the incident beam. Averaged values of hydrodynamic radius were calculated by a cumulant method using the software provided by the manufacturer (Zetasizer Software v7.12; Malvern Panalytical Ltd.). The measurements were conducted in quadruplicate.

2D ^{1}H–^{15}N NMR Experiments. To prepare a stock solution of ^{15}N-labeled Aβ_{40}, uniformly ^{15}N-labeled Aβ_{40} was dissolved in NH_4OH (1% w/w, aq), lyophilized for 48 h, and stored at –80 °C. To remove possible preformed aggregates, the lyophilized peptide was then redissolved in diluted NaOH (pH 10) and passed through a filter (0.22 μm). The solution of ^{15}N-labeled Aβ_{40} (20 μM) was prepared with and without ZF2–4 or ZFx' (200 μM) in 20 mM HEPES, pH 7.4, 1 mM DTT, 10% v/v D_2O for 2D ^{1}H–^{15}N selective optimized flip-angle short-transient heteronuclear multiple-quantum correlation (SOFAST–HMQC) NMR experiments. In the case of ^{15}N-labeled ZF2–4, the undissolved precipitates in its stock solution prepared in 25 mM MOPS, pH 7.4, 50 mM NaCl were removed by centrifugation at 40,000 rpm for 1 h. The solution of ^{15}N-labeled ZF2–4 (50 μM) was prepared with and without Aβ_{40} (100 μM) in 20

mM HEPES, pH 7.4, 1 mM DTT, 10% v/v D_2O for 2D ^1H–^{15}N heteronuclear single quantum coherence (HSQC) NMR experiments. The SOFAST–HMQC and HSQC spectra were acquired with 180 and 24 scans, respectively, at 10 °C. Dimensions of the SOFAST–HMQC and HSQC spectra were 1,024 (^1H) × 128 (^{15}N) and 2,048 (^1H) × 256 (^{15}N) points, respectively. Experimental data were processed with NMRpipe[16] and Sparky 3.115.[17] The ^1H and ^{15}N assignments of ^{15}N-labeled Aβ_{40} were determined based on the previous report.[18,19] The chemical shift perturbation was calculated following the equation below.[20] Note that $\Delta\delta_H$ and $\Delta\delta_N$ represent the changes in the chemical shifts of ^1H and ^{15}N of ^{15}N-labeled Aβ_{40} or ^{15}N-labeled ZF2–4, respectively.

$$\Delta\delta_{NH} = \sqrt{(\Delta\delta_H)^2 + \left(\frac{\Delta\delta_N}{6.5}\right)^2}$$

ITC. The solutions of Aβ_{40} and ZF2–4 were centrifuged at 10,000 × *g* for 3 min to remove visible air bubbles prior to loading into the instrument (Malvern Panalytical Ltd.). ZF2–4 (300 µM; 20 mM HEPES, pH 7.4, 1 mM DTT) in the syringe was titrated into Aβ_{40} (20 µM; 20 mM HEPES, pH 7.4, 1 mM DTT) in the sample cell at 10 °C using 19 injections. The first injection volume was 0.4 µL to minimize artifacts arising from air bubbles, followed by eighteen 2 µL injections. The sample cell was stirred continuously at 750 rpm. The initial delay and the reference power were set to 180 s and 10 µcal/s, respectively. The heat of dilution was determined by titrating ZF2–4 into the corresponding buffered solution (20 mM HEPES, pH 7.4, 1 mM DTT) under identical conditions. Raw thermograms were baseline-corrected, and the heat of dilution was subtracted to generate the binding isotherm. The resulting isotherm was fitted using a one-set-of-sites binding model with the software provided by Malvern Panalytical Ltd. The reported errors for the thermodynamic parameters represent the fitting errors.

Fluorescence-Quenching Measurements. The K_d value for the ZF2–4–Aβ_{40} complex was measured by fluorescence spectroscopy employing HiLyte™ Fluor 488-labeled Aβ_{40} (^{HF488}Aβ_{40}). Various concentrations of ZF2–4 were titrated into the solution of ^{HF488}Aβ_{40} (320 nM) in 20 mM HEPES, pH 7.4, 1 mM DTT. Upon treatment of ZF2–4, the intrinsic fluorescence of ^{HF488}Aβ_{40} was quenched (JASCO Inc., Tokyo, Japan; λ_{ex} = 503 nm; λ_{em} = 507–675 nm). The change in fluorescence was plotted as a function of [ZF2–4]. The plot was then fitted using the equation below to obtain the K_d value of the ZF2–4–Aβ_{40} complex.[21] Data are presented as mean ± s.e.m. (standard error of the mean) of three independent experiments. The

measurements were conducted in triplicate.

$$\frac{\Delta F}{F_0} = \frac{K_d + [^{HF488}A\beta_{40}]_{total} + [ZF2\text{–}4] - \sqrt{(K_d + [^{HF488}A\beta_{40}]_{total} + [ZF2\text{–}4]^2 - 4 \times [^{HF488}A\beta_{40}]_{total} \times [ZF2\text{–}4]}}{2 \times [^{HF488}A\beta_{40}]_{total}}$$

Job Plot. To determine the binding stoichiometry between ZF2–4 and Aβ_{40}, the ^{HF488}Aβ_{40} fluorescence was recorded (HORIBA, Kyoto, Japan; λ_{ex} = 503 nm; λ_{em} = 528 nm) by varying the molar ratio between ZF2–4 and ^{HF488}Aβ_{40} under the fixed total concentration (640 nM) in 20 mM HEPES, pH 7.4, 1 mM DTT. Normalized $\Delta F/F_0$ values were plotted as a function of the molar fraction of ^{HF488}Aβ_{40} to obtain the Job plot. Data are presented as mean ± s.e.m. of three independent experiments. The measurements were conducted in triplicate.

LLPS Studies. To monitor droplet formation, Aβ_{40} (100 μM) containing 10% v/v fluorophore-conjugated ^{HF488}Aβ_{40} was incubated with and without ZF2–4 (100 μM) for 3 h at 37 °C in 20 mM KPi, pH 7.4, 75 mM NaCl, 20% w/v PEG, 0.02% w/v NaN_3, 1 mM DTT under quiescent conditions. Aliquots (10 μL) were drop-cast onto glass slides (Sigma-Aldrich), covered with coverslips (Fisher, Hampton, NH, USA), and sealed using a PAP pen (Sigma-Aldrich). Phase-separated droplets were imaged using a Zeiss LSM 980 confocal microscope (λ_{ex} = 488 nm) from at least five randomly selected fields per condition. Droplet radii were quantified using the Fiji software.

Turbidity Assay. Aβ_{40} (100 μM) with ZF2–4, ZFx', or Mx (25, 50, 100, and 200 μM) were incubated at 37 °C in 20 mM HEPES, pH 7.4, 150 mM NaCl, 1 mM DTT under quiescent conditions. All experiments were prepared in a 96-well half-area white microplate with the clear flat bottom that has a non-binding surface coated with polystyrene (Corning, Kennebunk, ME, USA). The absorbance value was measured by a microplate reader (Molecular Devices, Sunnyvale, CA, USA; λ_{abs} = 600 nm). The kinetic parameters of Aβ_{40} aggregation in the absence and presence of ZF2–4, ZF3', M13, or M30 were acquired by fitting the absorbance curves using a modified Boltzmann-sigmoidal equation below in the Origin software.[22] I_0 and A are the initial absorbance intensity and amplitude, respectively. The $t_{1/2}$ value indicates the half time when absorbance reaches 50% of its maximum intensity, while k is the rate constant of the elongation phase. The lag time, t_{lag}, was defined using an equation below.[23]

$$I(t) = I_0 + \frac{A}{1+ e^{-k\left(t-t_{1/2}\right)}}$$

$$t_{lag} = t_{1/2} - 2(1/k)$$

The kinetic parameters of Aβ_{40} aggregation in the presence of ZF2', ZF4', M28, or M32 were obtained by fitting the absorbance curves using a double Boltzmann-sigmoidal equation below in the Origin software.[24] I_0 and A are the initial and maximum absorbance intensities, respectively, while p and $1 - p$ are the fractions of the curve comprising phase 1 and 2, respectively. k_1 and k_2 are the constant intervals that control the rise of phase 1 and 2, respectively. The t_{c1} and t_{c2} values indicate the half time when absorbance reaches 50% of its maximum intensity of each phase 1 and 2, respectively. Data are presented as mean ± s.e.m. of three independent experiments.

$$I(t) = I_0 + A\left[\frac{p}{1+ e^{(t-t_{c1})/k_1}} + \frac{1-p}{1+ e^{(t-t_{c2})/k_2}}\right]$$

Gel/Western Blot. Aβ_{40} (100 µM) and ZF2–4 (25, 50, 100, and 200 µM) were incubated in the buffered solution for 0.6, 2, 4, and 8 h at 37 °C under quiescent conditions. The samples were prepared using Eppendorf tubes (Hamburg, Germany). The resultant peptide species were analyzed by gel/Western blot using an anti-Aβ antibody (6E10; #803003, BioLegend, San Diego, CA, USA).[2] Each sample (10 µL) was separated onto a tricine gel (10–20% w/v acrylamide; Invitrogen, Carlsbad, CA, USA). Following separation, the peptides were transferred onto nitrocellulose membranes and blocked with bovine serum albumin (BSA, 3% w/v; Sigma-Aldrich) in Tris-buffered saline (TBS) containing 0.1% v/v Tween-20 (Sigma-Aldrich) (TBS-T) for 4 h at room temperature or overnight at 4 °C. The membranes were incubated with 6E10 (1:2,000) in the solution of BSA (2% w/v in TBS-T) overnight at room temperature. After washing with TBS-T three times (10 min), the goat anti-mouse secondary antibody (1:5,000; Cayman, Ann Arbor, MI, USA) in the solution of BSA (2% w/v in TBS-T) was added for 1.5 h at room temperature. A homemade ECL kit[25] was used to visualize the images gained by gel/Western blot on a ChemiDoc MP Imaging System (Bio-Rad, Hercules, CA, USA). The measurements were conducted in triplicate.

TEM. Samples for TEM measurements were prepared following the previously reported methods.[2] Glow-discharged grids (Formvar/Carbon 300-mesh; Electron Microscopy Sciences,

Hatfield, PA, USA) were treated with peptide samples (7.5 μL) for 2 min at room temperature. Excess sample was removed using filter paper followed by washing with ddH_2O three times. Each grid incubated with uranyl acetate (1% w/v ddH_2O; 7.5 μL) for 2 min was blotted off and dried overnight at room temperature. Locations of the samples on the grids were randomly picked for taking more than 20 images per each grid.

CD Spectroscopy. The secondary structures of $A\beta_{40}$ and ZF2–4 were analyzed by CD spectroscopy.[26-28] $A\beta_{40}$ (100 μM) was incubated in the absence and presence of ZF2–4 (25 μM) in 20 mM HEPES, pH 7.4, 150 mM NaF, 1 mM DTT. The samples were incubated at 37 °C under quiescent conditions. The samples were prepared using Eppendorf tubes. Similarly, to identify the secondary structures of ZF2', ZF3', and ZF4', the peptides (100 μM) were prepared in 20 mM HEPES, pH 7.4, 150 mM NaF, 1 mM DTT. CD spectra of the samples were collected in a range from 195 to 255 nm with a cell path length of 0.5 mm. The digital integration time, bandwidth, and scanning speed were 4 s, 2 nm, and 20 nm/min, respectively. Each spectrum was smoothed by Fourier transforms. The secondary structure of ZF2', ZF3', and ZF4' was analyzed following the previously reported BeStSel method.[27] The measurements were conducted in triplicate.

ThT Fluorescence Assay. Aβ (20 μM) and ZF2–4 (5, 10, 20, and 40 μM) were incubated with ThT (20 μM) at 37 °C in 20 mM HEPES, pH 7.4, 150 mM NaCl, 1 mM DTT under quiescent conditions. All experiments were prepared in a 96-well half-area black microplate that has a non-binding surface coated with polystyrene (Corning). The fluorescence intensity was measured by a microplate reader (Molecular Devices; λ_{ex} = 440 nm; λ_{em} = 490 nm). The kinetic parameters of Aβ aggregation in the absence and presence of ZF2–4 were acquired by fitting the fluorescence curves using a modified Boltzmann-sigmoidal equation below in the Origin software.[22] F_0 and A are the initial fluorescence intensity and amplitude, respectively. The $t_{1/2}$ value indicates the half time when fluorescence reaches 50% of its maximum intensity, while k is the rate constant of the elongation phase. The t_{lag} was defined using an equation below.[23] Data are presented as mean ± s.e.m. of three independent experiments.

$$F(t) = F_0 + \frac{A}{1 + e^{-k\left(t - t_{1/2}\right)}}$$

$$t_{lag} = t_{1/2} - 2(1/k)$$

ANS Fluorescence Assay. Aβ_{40} (20 μM) and ZF2–4, ZFx', or Mx (5, 10, 20, and 40 μM) were incubated with ANS (100 μM) at 37 °C in 20 mM HEPES, pH 7.4, 150 mM NaCl, 1 mM DTT under quiescent conditions. All experiments were prepared in a 96-well half-area black microplate that has a non-binding surface coated with polystyrene (Corning). The fluorescence intensity was measured by a microplate reader (Molecular Devices; λ_{ex} = 350 nm; λ_{em} = 480 nm). The kinetic parameters of Aβ aggregation in the absence and presence of ZF2–4 were acquired by fitting the fluorescence curves using a modified Boltzmann-sigmoidal equation below in the Origin software.[22] F_0 and A are the initial fluorescence intensity and amplitude, respectively. The $t_{1/2}$ value indicates the half time when fluorescence reaches 50% of its maximum intensity, while k is the rate constant of the elongation phase. The t_{lag} was defined using an equation below.[23] Data are presented as mean ± s.e.m. of three independent experiments.

$$F(t) = F_0 + \frac{A}{1 + e^{-k\left(t - t_{1/2}\right)}}$$

$$t_{lag} = t_{1/2} - 2(1/k)$$

Cell Culture and Cell Viability Measurements. The human neuroblastoma SH-SY5Y cell line was purchased from the American Type Culture Collection (ATCC; Manassas, VA, USA). The cell line was maintained in the media containing 50% v/v minimum essential medium (MEM; GIBCO, Grand Island, NY, USA) and 50% v/v F-12 (GIBCO) supplemented with 10% v/v fetal bovine serum (FBS; Sigma-Aldrich) and 100 U/mL penicillin with 100 mg/mL streptomycin (GIBCO). The cells were grown and maintained at 37 °C in a humidified atmosphere with 5% CO_2. The cells used for our studies did not indicate mycoplasma contamination. Cell viability was determined by the MTT assay [MTT = 3-(4,5-dimethylthiazol-2-yl)-2,5-diphenyltetrazolium bromide; Biosesang, Seoul, Republic of Korea]. Aβ_{40} (100 μM) with ZF2–4, ZFx', or Mx (25, 50, 100, and 200 μM) were pre-incubated for 8 h at 37 °C under quiescent conditions. The samples were prepared using Eppendorf tubes. Cells were seeded in a 96-well microplate (10,000 cells/100 μL) and treated with the samples to make the final concentrations as 10 μM for Aβ_{40} and 2.5, 5, 10, and 20 μM for ZF2–4, ZFx', or Mx. After 48 h of incubation, MTT [5 mg/mL in PBS (pH 7.4, GIBCO); 25 μL] was added to each well and the plate was incubated for 4 h at 37 °C. Formazan produced by cells was solubilized using an acidic solution of *N*,*N*-dimethylformamide (DMF; pH 4.5, 50% v/v, aq) and SDS (20%

w/v; Wako Chemicals, Osaka, Japan) overnight at room temperature in the dark. Absorbance was measured at 600 nm by a microplate reader (Molecular Devices). Cell viability was calculated, compared to that of the cells treated with an equivalent amount of the buffered solution (20 mM HEPES, pH 7.4, 150 mM NaCl). Data are presented as mean ± s.e.m. of three independent experiments. For statistical analysis, the Student's *t*-test was used.

Cell Culture and Microinjection Studies. SH-SY5Y cells were maintained in medium containing 50% v/v Dulbecco's modified eagle medium and 50% v/v nutrient mixture F-12 (phenol red-free DMEM/F-12; GIBCO), supplemented with 10% v/v FBS (GIBCO) and 100 U/mL penicillin-streptomycin (GIBCO). Cells were cultured at 37 °C in a humidified atmosphere with 5% CO_2. The cells used in this study were confirmed to be free of mycoplasma contamination. Cells were seeded onto imaging dishes (35 mm) at a density of 1.0×10^5 cells in 1 mL of culture medium and incubated for 48 h prior to microinjection. Protein or peptide solutions were centrifuged at 5,000 rpm for 1 min to remove large aggregates that could clog the microinjection glass capillary. Solutions containing $A\beta_{40}$ (10 μM), ZF2–4 or ZF2' (10 μM), or both were mixed with Texas Red-labeled dextran (0.5 mg/mL; MW = 3,000; Invitrogen) and microinjected into more than 250 cells per experimental condition using an InjectMan 4 micromanipulator and a Femtojet 4i microinjector. The injection pressure (P_i), compensation pressure (P_c), and injection time (t_i) were set to 50 hPa, 20 hPa, and 0.2 s, respectively.

Cell viability was determined according to a previously reported procedure.[29] The number of Texas Red-positive cells detected 3 h after microinjection was defined as 100% cell viability, thereby excluding cells damaged during the microinjection procedure. After a total incubation period of 48 h (45 h after the initial assessment), the remaining Texas Red-positive cells were counted, and cell viability was calculated relative to the number of successfully microinjected cells detected at the 3 h time point. More than 250 cells were microinjected for each experimental condition to ensure that at least 100 successfully microinjected cells were available for quantitative analysis. Experiments were performed in at least three independent biological replicates, and data are presented as mean ± s.e.m. Fluorescence images were acquired using a fluorescence microscope equipped with an RFP filter set [λ_{ex} = 531 (± 40) nm; λ_{em} = 593 (± 40) nm]. Statistical analyses were performed using Student's *t*-test.

Cellular and *Ex Vivo* Co-Localization of ZF2–4 with Aβ. To visualize the co-localization between ZF2–4 and Aβ under cellular conditions, SH-SY5Y cells were seeded on an imaging dish (35 mm; 1.5×10^5 cells per 1 mL) and incubated for 48 h. Cells were first treated with

$A\beta_{40}$ (50 μM), followed by the addition of ^{HF647}ZF2–4 (50 μM) after 2 h, to make the final concentrations as 5 μM for both $A\beta_{40}$ and ^{HF647}ZF2–4. After an additional 12 h of incubation, cellular membranes were stained with the CellBrite™ green cytoplasmic membrane dye (1:2,000 in growth medium; Biotium, San Francisco, CA, USA) for 30 min. After washing twice with fresh growth medium for 5 min each, cells were fixed with 4% w/v formaldehyde (Sigma-Aldrich) in PBS (GIBCO) for 15 min. Following fixation, cells were blocked with 3% w/v BSA in TBS (Sigma-Aldrich) for 1.5 h at room temperature and subsequently incubated with 6E10 (1:2,000; #803003; BioLegend) in TBS containing 2% w/v BSA for 2 h. After washing four times with TBS for 5 min each, cells were incubated with an Alexa Fluor™ 350-conjugated donkey anti-mouse secondary antibody (1:1,000; #A-10035; Thermo Fisher Scientific, Waltham, MA, USA) in TBS containing 2% w/v BSA for 1 h at room temperature. Cells were then washed four times with TBS for 5 min each and stored at 4 °C until imaging.

For *ex vivo* experiments, coronal brain sections obtained from 6-month-old APP/PS1 Tg mice were washed with PBS, treated with ^{HF647}ZF2–4 (5 μM), and incubated for 12 h at 4 °C. The sections were then blocked for 1 h at room temperature in PBS containing 1% w/v BSA, 5% v/v donkey serum, and 0.3% v/v Triton X-100, followed by overnight incubation at 4 °C with 6E10 (1:500; #803003; BioLegend). After washing three times with PBS for 10 min each, the sections were incubated with an Alexa Fluor™ 350-conjugated donkey anti-mouse secondary antibody (1:500; #A-10035; Thermo Fisher Scientific) for 2 h at room temperature. The sections were subsequently washed three times with PBS for 10 min each and mounted onto silane-coated glass slides using VECTASHIELD Antifade Mounting Medium (#H-1000; Vector Laboratories, Newark, CA, USA). At least six randomly selected fields per condition were imaged using a Zeiss LSM 980 confocal microscope. To minimize spectral crosstalk among fluorescence channels, images were acquired by sequential scanning using excitation wavelengths of 633 nm for ^{HF647}ZF2–4 and 405 nm for the Alexa Fluor™ 350-conjugated secondary antibody.

Live-Cell Imaging Experiments. To characterize the interaction between Aβ and cell membranes, SH-SY5Y cells were plated on an imaging dish (35 mm; 4.5 x 10^5 cells per 1 mL) and incubated for 48 h. $A\beta_{40}$ (100 μM) and ZF2–4 (100 μM) were pre-incubated for 8 h at 37 °C under quiescent conditions. The samples were prepared using Eppendorf tubes. Cells were treated with the resultant samples to make the final concentrations as 10 μM for both $A\beta_{40}$ and ZF2–4. After 48 h of incubation, cellular membranes were stained with the CellBrite™ green cytoplasmic membrane dye (1:2,000 in growth medium; Biotium) for 30 min. After washing

with the fresh growth medium two times (5 min), cells were fixed with 4% w/v formaldehyde (Sigma-Aldrich) in PBS (GIBCO) for 15 min. Following the fixation, the cells were blocked with BSA (3% w/v in TBS; Sigma-Aldrich) for 1.5 h at room temperature. The cells were incubated with 6E10 (1:2,000; #803003, BioLegend) in the solution of BSA (2% w/v in TBS) for 2 h. After washing with TBS (4x, 5 min), the Alexa Fluor 647-goat anti-mouse secondary antibody (1:1,000; Invitrogen) in the solution of BSA (2% w/v in TBS) was added for 1 h at room temperature. The cells were then washed with TBS (4x, 5 min) and stored at 4 °C. At least four randomly selected fields per condition were captured with a Zeiss LSM 980 confocal microscope. To avoid crosstalk interference between multiple fluorescent channels, fluorescent images were sequentially scanned (λ_{ex} = 488 nm for a cellular membrane dye; λ_{ex} = 633 nm for an anti-mouse secondary antibody). The Manders' correlation coefficient was calculated using the Coloc2 plugin in Fiji software. This coefficient indicates the degree of co-localization between the quantities of interest, independent on signal proportionality.[30]

Furthermore, for imaging the oligomeric and fibrillar aggregates inside the cells, cells were plated on an imaging dish (35 mm; 1.0 x 10^5 cells per 1 mL) and incubated for 48 h before microinjection. Solutions of a protein or peptide were centrifuged at 5,000 rpm for 1 min to remove large aggregates that can clog a microinjection glass capillary. The solutions of Aβ_{40} (10 μM) with and without ZF2–4 or ZF2' (10 μM) were mixed followed by injection into the cells using an InjectMan 4 micromanipulator and a Femtojet 4i microinjector. The P_i, P_c, and t_i were set to 50 hPa, 20 hPa, and 0.2 s, respectively. After 48 h of incubation, to visualize the distribution of intracellular Aβ_{40} species, cells were stained with antibodies (6E10 for Aβ species or A11 for Aβ oligomers) or a fluorescent dye [thioflavin-S (ThS) for Aβ fibrils]. The cells were first fixed with 4% w/v formaldehyde (Sigma-Aldrich) in PBS (GIBCO) for 15 min, permeabilized in cold methanol for 30 min, washed three times with PBS (GIBCO) for 8 min, incubated with the blocking solution, BSA (3% w/v in TBS; Sigma-Aldrich), for 1.5 h at room temperature, and incubated with 6E10 (1:1,000; #803003, BioLegend) or A11 (1:500; AHB0052, Merck KGaA, Damstadt, Germany) diluted with BSA (2% w/v in TBS; Sigma-Aldrich) overnight at 4 °C. Afterward, the cells were washed four times with TBS (5 min), incubated with fluorescent-conjugated secondary antibodies [for 6E10, Alexa Fluor 647-goat anti-mouse secondary antibody (1:1,000; Invitrogen); for A11, Cy3^{TM}-goat anti-rabbit secondary antibody (1:1,000; Invitrogen)] in the solution of BSA (2% w/v in TBS; Sigma-Aldrich) for 2 h at room temperature, and washed three times with TBS for 8 min. For ThS staining, cells were incubated for 8 min (dark) at a concentration of 0.125% w/v ThS (Sigma-Aldrich) dissolved in 50% v/v ethanol (filtration before use) and washed two times in 50% v/v

PBS/ethanol for 3 min followed by washing three times in PBS for 2 min. Following the staining, cells were imaged by a Zeiss LSM 980 confocal microscope (λ_{ex} = 633 nm for an anti-mouse secondary antibody; λ_{ex} = 561 nm for an anti-rabbit secondary antibody; λ_{ex} = 488 nm for ThS). The signal intensities of 6E10-, A11-, or ThS-positive Aβ species, respectively, were quantified using Fiji software.

PAMPA–BBB. PAMPA–BBB measurements for ZF2–4–P9 were conducted employing the PAMPA explorer kit (*p*ION, Inc. Billerica, MA, USA) following previously reported protocols with minor modifications.[31,32] ZF2–4–P9 was diluted with Prisma HT buffer (pH 7.4; *p*ION) to a final concentration of 100 μM and then added to the wells in the donor plate (200 μL, number of replicates = 12). BBB-1 lipid formulation (5 μL; *p*ION) was used to coat the polyvinylidene fluoride (PVDF; 0.45 μm) filter membrane on the acceptor plate. The acceptor plate was placed on top of the donor plate, forming a sandwich, and the brain sink buffer (200 μL; *p*ION) was treated to each well of the acceptor plate. The sandwich was incubated for 4 h at ambient temperature without stirring. Electronic absorbance spectra of the solutions in the blank, reference, donor, and acceptor plates were measured on a microplate reader. The optical density (OD) of a compound in the reference, donor, and acceptor wells (C^{Ref}, C^{Don}, and C^{Acc}, respectively) was calculated by subtracting the background intensity ($\mathrm{OD_{Blank}}$). These values were used to determine the membrane retention factor (R_{m}) and lag time (τ_{lag}) according to the following equations:

$$R_{\mathrm{m}} = 1 - \frac{C^{\mathrm{Don}}}{C^{\mathrm{Ref}}} - \frac{C^{\mathrm{Acc}}}{C^{\mathrm{Ref}}}$$

$$\tau_{\mathrm{lag}} = (54R_{\mathrm{m}} + 1) \times 60$$

The $-\log P_{\mathrm{e}}$ value was calculated using:

$$-\log P_{\mathrm{e}} = -\log_{10}[-\frac{2.303 \times V_d}{A \times (t - \tau_{\mathrm{lag}})} \times \log_{10}\{\frac{1}{1 - R_{\mathrm{m}}} \cdot \frac{C_d(t)}{C_d(0)}\}]$$

Here, V_d is the donor well volume (0.2 cm^3); A is the membrane area (0.3 cm^2); t is the incubation time (4 h); $C_d(t)$ is the absorbance of the compound in the donor well at t; $C_d(0)$ is the initial absorbance of the compound in the donor well. Central nervous system (CNS) +/–

designations were assigned by referring to compounds that were identified in previous reports.[33,34]

***In Vivo* Biodistribution Studies.** C57BL/6J (B6) mice were purchased from the Jackson Laboratory (Bar Harbor, ME, USA) and maintained at KRIBB (Daejeon, Republic of Korea). Eight-week-old male B6 mice (25–27 g) were used for the experiments. All animal procedures were approved by the Institutional Animal Care and Use Committee of KRIBB and performed in accordance with the institutional guidelines (approval number, KRIBB-AEC-26267). For biodistribution studies, ZF2–4–P9 was administered into B6 mice *i.p.* at a dose of 3 mg/kg. At 10 min post-administration, the animals were sacrificed. Blood samples were collected *via* retro-orbital puncture, immediately transferred into K_2EDTA tubes, and centrifuged at 5,000 × g for 15 min at 4 °C to obtain plasma within 30 min of collection. Plasma samples were stored at −80 °C until analysis. The brains, livers, and kidneys were collected, thoroughly rinsed with physiological saline, flash-frozen in liquid N_2, and stored at −80 °C until analysis. Each organ sample was weighed and homogenized with 1 mL of H_2O using a tissue homogenizer.

For the quantification of ZF2–4–P9, plasma and homogenized organ samples (100 μL) were mixed with H_2O (900 μL; LC-grade) and incubated for 30 min at 4 °C. The samples were then centrifuged at 13,000 rpm for 10 min at 4 °C, and the resulting supernatants were subjected to liquid chromatography–mass spectrometry (LC–MS) analysis using a Waters Xevo TQ-S micro triple quadrupole mass spectrometer. The LC–MS system was operated in the positive ion mode. The chromatographic separation of ZF2–4–P9 was conducted on an ACQUITY UPLC® Protein BEH C4 column (1.7 μm; 2.1 × 100 mm; Milford, MA, USA) (sample injection volume, 10 μL; flow rate, 0.45 mL/min). The mobile phase was pumped with isocratic elution of H_2O containing 0.1% v/v formic acid:CH_3CN containing 0.1% v/v formic acid (95:5, v/v). ZF2–4–P9 was detected by selected ion monitoring at *m/z* 715.796 with a retention time of 6.49 min. Data acquisition and analysis were performed using MassLynx (version 5.0) and TargetLynx (version 5.0), respectively.

Animals and Administration of ZF2–4–P9. B6;C3-Tg(APPswe, PSEN1ΔE9)85Dbo/Mmjax (APP/PS1) mice, an Alzheimer's disease mouse model, were purchased from the Jackson Laboratory (Bar Harbor) and bred in a hemizygote state by crossing C57BL/6J × C3H F1 hybrid mice and non-transgenic C57BL/6J × C3H F1 mice at the KRIBB. A mouse in which the inserted gene is not expressed was used as a non-Tg control. The APP/PS1 mouse is a double Tg mouse encoding mutant amyloid precursor protein (Swedish, K670N and M671L) and

mutant presenilin1 (PSEN1-ΔE9).[35] These mutations are known to accelerate the accumulation of Aβ aggregates.[36] Amyloid plaques first appear in the brains of APP/PS1 female mice around 4 months of age, resulting in cognitive impairment around 6–7 months of age.[37,38] Female Tg mice and non-Tg littermates were housed separately in standard polycarbonate plastic cages (4–5 mice per cage) until 5 months of age. All mice were housed in a specific pathogen-free environment with a 12 h-light and 12 h-dark cycle (light on at 7:00 AM), 50–60% humidity, and 21 (± 2) °C temperature, and were fed a standard chow diet (2018s, Envigo Teklad, Madison, WI, USA) with free access to water. All animal experiments were approved by the Institutional Animal Care and Use Committee of KRIBB and were performed in accordance with the institutional guidelines at KRIBB (approval number, KRIBB-AEC-25182).

ZF2–4–P9 was diluted with 20 mM HEPES, pH 7.4, 150 mM NaCl, 1mM DTT and stored at −80 °C with protection from light until use. The prepared samples were administered intraperitoneally to mice at a dose of 3 mg/kg of body weight daily for 38 days. The Veh solution (20 mM HEPES, pH 7.4, 150 mM NaCl, 1 mM DTT) was administered to mice in the same manner. APP/PS1 Tg mice were randomly divided into two groups: Veh-treated Tg mice (n = 9); ZF2–4–P9-treated Tg mice (n = 7); Veh-treated wild-type mice (WT, n = 10) as a control group.

Histochemical Analysis. On the last day of the injection, the mice were sacrificed rapidly by cervical dislocation, and their brains were stored in 4% w/v paraformaldehyde. The brains were sectioned into 40 μm thick sections using a vibratome (Vibratome VT1000S, Leica, Wetzlar, Germany). Free-floating sections were incubated with 3% v/v H_2O_2 in PBS for 10 min and blocked with 4% w/v BSA for 1 h at room temperature. Sections were then incubated with a mouse monoclonal Bam-10 antibody (1:1,000, A5213, Sigma-Aldrich), a rabbit polyclonal ionized calcium-binding adapter molecule 1 antibody (Iba-1, 019-19741, 5% HS, Wako Chemicals), or a glial fibrillary acidic protein antibody (GFAP, Z0334, 5% GS, Dako, Carpinteria, CA, USA) overnight at 4 °C. Tissues were then sequentially incubated for 1 h with a biotinylated goat anti-mouse IgG antibody (1:200, BA-9200, Vector, Burlingame, CA, USA) or a biotinylated anti-rabbit IgG antibody (1:200, BA-1000, Vector) followed by incubation with an avidin/biotin complex (ABC) kit (PK-4000). Tissues were developed by 3,3’-diaminobenzidine (sc-216567, Santa Cruz, Dallas, TX, USA) and mounted on silane-coated slide glass (5116-20F, Muto-glass, Tokyo, Japan) with Canada balsam (C1795, Sigma-Aldrich). Mounted tissues were visualized using an Olympus DP74 digital camera (Olympus, Tokyo, Japan).

For ThS staining, free-floating sections were washed in PBS, mounted onto slide glasses, and air-dried at room temperature. ThS (Sigma-Aldrich) was prepared in 50% w/v ethanol at a final concentration of 1% w/v and filtered through a 0.22 μm polyethersulfone membrane before use. The sections were hydrated through a graded ethanol series (80%, 70%, and 50%) and incubated with the ThS solution for 15 min at room temperature. Following staining, the sections were sequentially washed with 50%, 70%, and 80% ethanol and rinsed with ddH_2O. The sections were subsequently stained with DAPI (0.5 μg/mL; Invitrogen) for 5 min and rinsed with sterile distilled water. Finally the slides were mounted using ProLong™ Gold Antifade Mountant (P36931; Invitrogen), and fluorescence images were acquired using an Olympus fluorescence microscope.

For Iba-1/Bam-10 co-staining, free-floating sections were blocked with 4% w/v BSA for 1 h at room temperature and then co-incubated overnight at 4 °C with a mouse monoclonal anti-Aβ antibody (Bam-10; 1:1,000; A5213, Sigma-Aldrich) and a rabbit polyclonal anti-Iba-1 antibody (019-19741; 5% HS; Wako Chemicals). The sections were subsequently incubated with Alexa Fluor™ 488-conjugated goat anti-mouse IgG (1:200; A10680; Invitrogen) and Alexa Fluor™ 594-conjugated goat anti-rabbit IgG (1:200; A11012; Invitrogen) for 1 h at room temperature in the dark. After washing, the sections were mounted onto silane-coated glass slides (5116-20F; Muto-glass) using ProLong™ Gold antifade reagent (P36931; Invitrogen). Images were acquired using a Zeiss LSM 800 confocal microscope. Bam-10-, Iba-1-, GFAP-, or ThS-positive areas in the parietal cortex and hippocampus were quantified using the MetaMorph image analysis system (Molecular Devices).

Immunoblot Analysis of Aβ. Parietal cortices were individually homogenized in a homogenization buffer (50 mM Tris-HCl, pH 8.0, 150 mM NaCl, 1% Nonidet P-40, 0.1% SDS, and 0.1% sodium deoxycholate), supplemented with a protease inhibitor cocktail (Roche, Mannheim, Germany). Following homogenization, the samples were centrifuged at 20,000 × *g* for 30 min at 4 °C. The resulting pellets were resuspended in 20 mM Tris-HCl, pH 7.4 containing 2% SDS and centrifuged at 25,000 × g for 30 min at 4 °C. The supernatants were collected as the SDS-soluble fractions and subjected to Western blot analysis. The SDS-soluble fractions (5 μg) were loaded onto Bol™ Bis-Tris Plus Mini Protein Gels (NW04122; Invitrogen), transferred onto PVDF membranes (Bio-Rad), and blocked with BSA (3% w/v) in TBS-T for 3 h at room temperature. The membranes were incubated overnight at room temperature with an anti-Aβ antibody (6E10; #803003; 1:2,000 dilution in TBS-T containing 2% w/v BSA). After washing with TBS-T (three times, 10 min each), the membranes were

incubated with an HRP-conjugated goat anti-mouse secondary antibody (#10004302; 1:5,000 dilution in TBS-T containing 2% w/v BSA) for 1 h at room temperature. Blots were visualized using an enhanced chemiluminescence detection kit (Thermo Fisher Scientific).

RT–PCR. For total RNA extraction, frontal cortices were individually homogenized with the TRIzol™ reagent (800 μL; 15596018; Invitrogen) using TissueLyser II (Qiagen). Following homogenization, chloroform (200 μL; C2432; Sigma-Aldrich) was added to each sample. After centrifugation at 16,600 × *g* for 10 min, the aqueous supernatant was collected, and RNA was precipitated with isopropanol (0918; VWR Life Science, Radnor, PA, USA), and washed with 75% ethanol (1 mL). After centrifugation at 16,600 × *g* for 15 min, the supernatant was removed, and the RNA pellet was dissolved in UltraPur™ DNase/RNase-Free Distilled Water (10977015; Invitrogen). RNA concentrations were determined using a NanoDrop ND-1000 spectrophotometer (Thermo Fisher Scientific). One microgram of total RNA was used for cDNA synthesis with a Reverse Transcription System (A3500; Promega, Madison, WI, USA) according to the manufacturer's protocols. For RT–PCR, cDNA was mixed with gene-specific primers and Power SYBR™ Green PCR Master Mix (4367659; Applied Biosystems, Warrington, UK). The *GAPDH* was used as the reference gene, and relative gene expression was determined using the comparative cycle threshold (Ct) method. Primer names and sequences are provided in Table S3.

Cognitive Behavior Evaluation. To identify the locomotor activity of mice after injection of ZF2–4–P9, an open field test was conducted 28 days after injection, as in the previous study.[39] Mice were placed in the center of the white chamber (45 × 45 × 45 cm^3) and freely explored for 30 min, after which they were returned to their home cages. The locomotion of the mice was recorded and analyzed using a video camera system, SMART (Panlab, Barcelona, Spain). The novel object recognition test (NOT) was conducted to determine recognition memory, as in the previous study.[38] After 30 days of administration, the mice were placed in the NOT chamber (40 × 20 × 20 cm^3) without objects for 5 min to allow habituation and then returned to their home cages. The mice were placed back into the NOT chamber containing two identical cylindrical objects (height, 10 cm; diameter, 2 cm) and allowed to explore freely for 10 min. After 24 h, one of the objects was replaced with a new square pillar (10 cm high, 2.5 cm wide, and 2 cm long), and the mice were placed in the chamber for 10 min. The movements of mice were recorded on video, and preference percentages were analyzed using the following formula: [(exploring the novel object or exploring the familiar object)/(exploring the novel object +

exploring the familiar object) × 100 (%)]. The object exploration was defined as touching and sniffing objects with forepaws or noses.

Blood Chemistry. At the end of the experimental period, blood samples were taken from the orbital venous congestion to determine the concentrations of plasma biomarkers. Plasma samples were prepared by centrifugation of the blood samples at 10,000 × *g* for 5 min at 4 °C, and the samples were stored at –80 °C until analysis. The plasma levels of ALT, AST, ALP, albumin, T-Bin, CREA, CK, and BUN were directly quantified using an automatic blood chemistry analyzer (AU480; Beckman Coulter, Krefeld, Germany).

Statistical Analysis. GraphPad Prism software (GraphPad Software, Inc., La Jolla, CA, USA) was used for the statistical analysis. Comparisons of two samples were performed with the Student's *t*-test, and comparisons of multiple samples were conducted with a one-way ANOVA followed by Tukey–Kramer's multiple-comparison test. Data were denoted as mean ± s.e.m. Statistical difference was considered significant at $^{*}P < 0.05$, $^{**}P < 0.01$, or $^{***}P < 0.001$.

Table S1. Kinetic parameters obtained from the turbidity assay.

	t_{lag} (h)	$t_{1/2,1}$ (h)	$t_{1/2,2}$ (h)	I_{max} / $I_{A\beta}$
**Aβ_{40}**	4.29 (± 0.01)	4.68 (± 0.01)	*n.d.*	1.00 (± 0.01)
+ **ZF2–4** (1 equiv)	0.76 (± 0.05)	1.37 (± 0.02)	*n.d.*	4.50 (± 0.04)
+ **ZF2'** (1 equiv)	*n.d.*	1.81 (± 0.01)	5.74 (± 0.02)	2.82 (± 0.02)
+ **ZF3'** (1 equiv)	3.87 (± 0.06)	4.57 (± 0.05)	*n.d.*	0.95 (± 0.03)
+ **ZF4'** (1 equiv)	*n.d.*	2.89 (± 0.01)	4.78 (± 0.02)	1.66 (± 0.02)
+ **M13** (1 equiv)	4.19 (± 0.07)	4.66 (± 0.03)	*n.d.*	0.86 (± 0.03)
+ **M28** (1 equiv)	*n.d.*	3.26 (± 0.01)	4.38 (± 0.02)	1.25 (± 0.02)
+ **M30** (1 equiv)	3.96 (± 0.05)	4.51 (± 0.05)	*n.d.*	0.88 (± 0.03)
+ **M32** (1 equiv)	*n.d.*	3.45 (± 0.01)	5.01 (± 0.03)	1.14 (± 0.02)

The kinetic parameters of Aβ_{40} incubated with and without ZF2–4 or its variants were obtained by fitting the absorbance curves using the modified Boltzmann-sigmoidal[22] or double Boltzmann-sigmoidal[24] equations. *n.a.*, not available; *n.d.*, not determined. It should be noted that the $t_{1/2,2}$ and t_{lag} values of Aβ_{40} aggregation showing monophasic and biphasic growths, respectively, are not defined.

Table S2. Concentrations of ZF2–4–P9 in the brain, liver, kidney, and plasma 10 min after the *i.p.* administration of ZF2–4–P9 (3 mg/kg) in mice.

Matrix	Concentration (ng/mL or ng/g)[a]	Distribution (%)	Organ:plasma ratio
Brain	185.5 (± 36.5)	6.2 (± 1.2)	4.1 (± 0.8)
Liver	41.5 (± 6.3)	1.4 (± 0.2)	0.9 (± 0.1)
Kidney	228.6 (± 87.4)	7.6 (± 2.9)	5.0 (± 1.9)
Plasma	45.7 (± 4.4)	1.5 (± 0.2)	–

[a]Mean (n = 3).

Table S3. Sequences of primer pairs.

	Forward (5' to 3')	Reverse (5' to 3')
Il-1β	CTA CAG GCT CCG AGA TGA ACA AC	TCC ATT GAG GTG GAG AGC TTT C
iNos	GTT CTC AGC CCA ACA ATA CAA GA	GTG GAC GGG TCG ATG TCA C
Tnf-α	CCC TCA CAC TCA GAT CAT CTT CT	GCT ACG ACG TGG GCT ACA G
Il-6	TCC ATC CAG TTG CCT TCT TG	TTC CAC GAT TTC CCA GAG AAC
Il-10	GGG TTG CCA AGC CTT ATC G	TCT CAC CCA GGG AAT TCA AAT G
Tgf-β	TAC CAT GCC AAC TTC TGT CTG GGA	ATG TTG GAC AAC TGC TCC ACC TTG
GAPDH	AGG TCG GTG TGA ACG GAT TTG	TGT AGA CCA TGT AGT TGA GGT CA

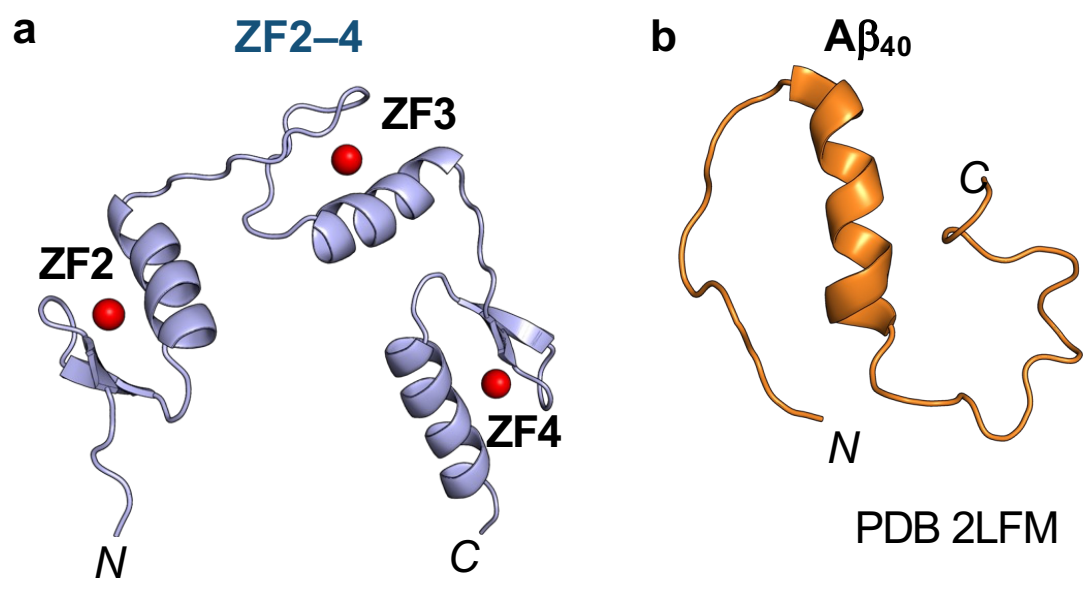

Figure S1. Structures of ZF2–4 and Aβ_{40} used for MD simulations. (a,b) Conformations of ZF2–4 predicted by AlphaFold2,[4,5] followed by docking three zinc ions, and Aβ_{40} (PDB 2LFM[7]). It should be noted that AlphaFold2 predicts ZF structures based on databases containing Zn(II)-bound forms.

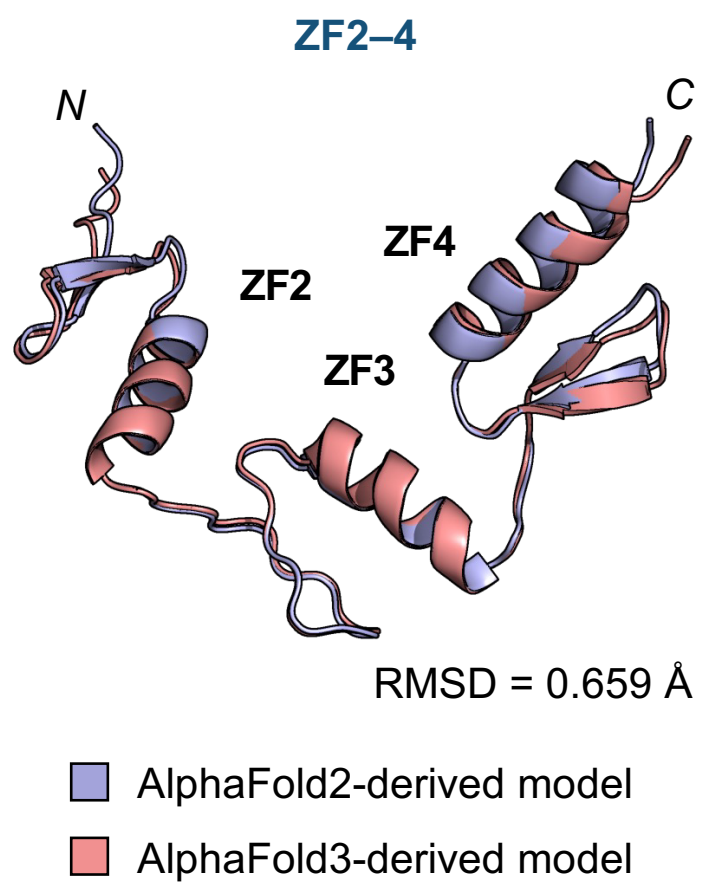

Figure S2. Structural homology of ZF2–4 predicted by AlphaFold2[4,5] and AlphaFold3.[6] Given the repetitive sequence pattern of classical ZF proteins ($X_4CX_2CX_{12}HX_3HX_3$) and AlphaFold's prediction logic, the structural homology is expected between the ZF models obtained by AlphaFold2 and AlphaFold3. Indeed, a significant structural concordance was observed between the AlphaFold2- and AlphaFold3-predicted ZF2–4, with the indication of RMSD below 1 Å. The structures of ZF2–4 predicted by AlphaFold2 and AlphaFold3 are depicted in lavender and salmon, respectively.

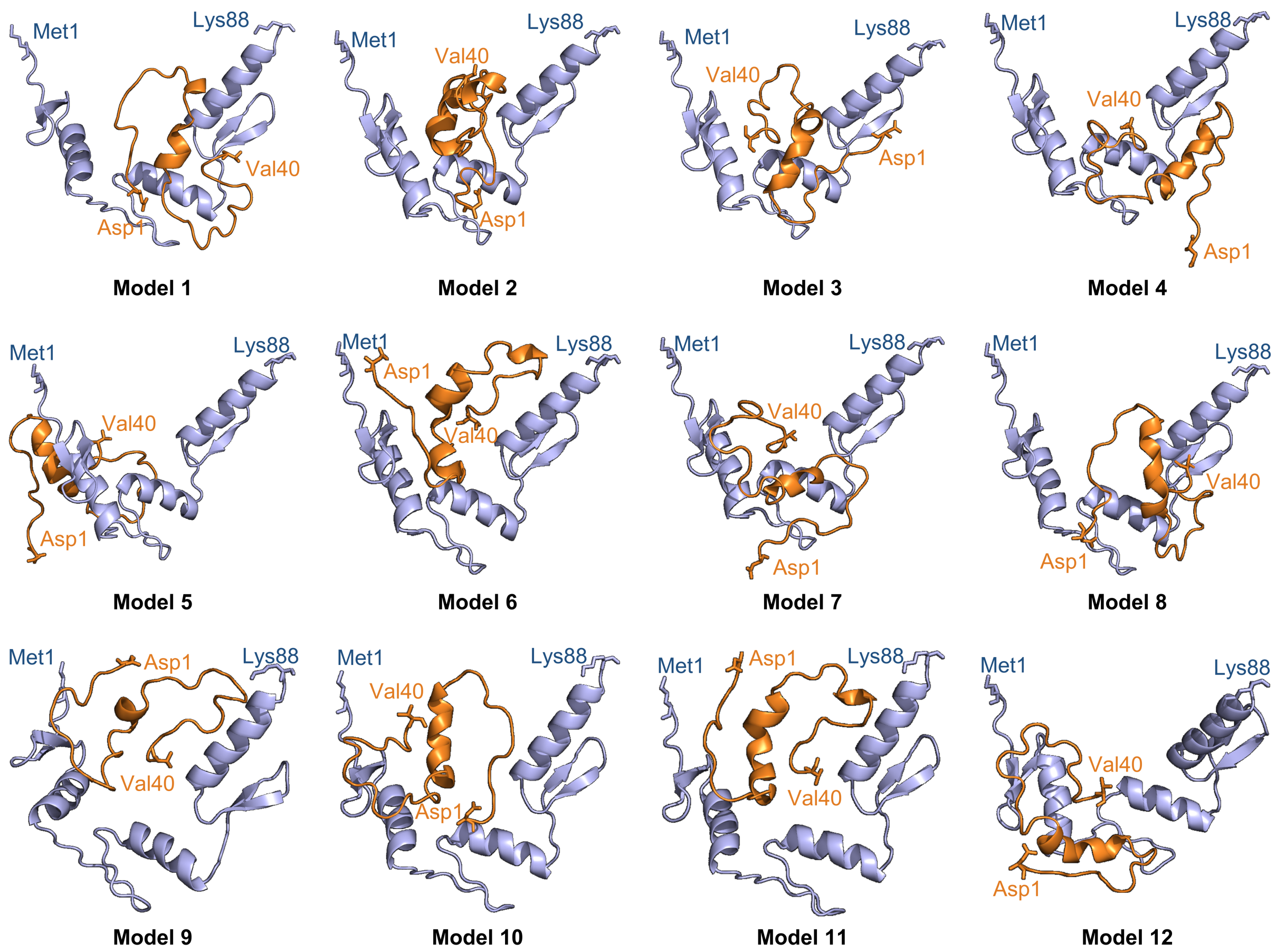

Figure S3. Docking models of ZF2–4–Aβ_{40} heterodimers. For docking studies, we used the structures of ZF2–4 predicted by AlphaFold2[4,5] and monomeric Aβ_{40} (PDB 2LFM[7]) derived from solution NMR experiments. It should be noted that AlphaFold2 predicts ZF structures based on databases containing Zn(II)-bound forms.

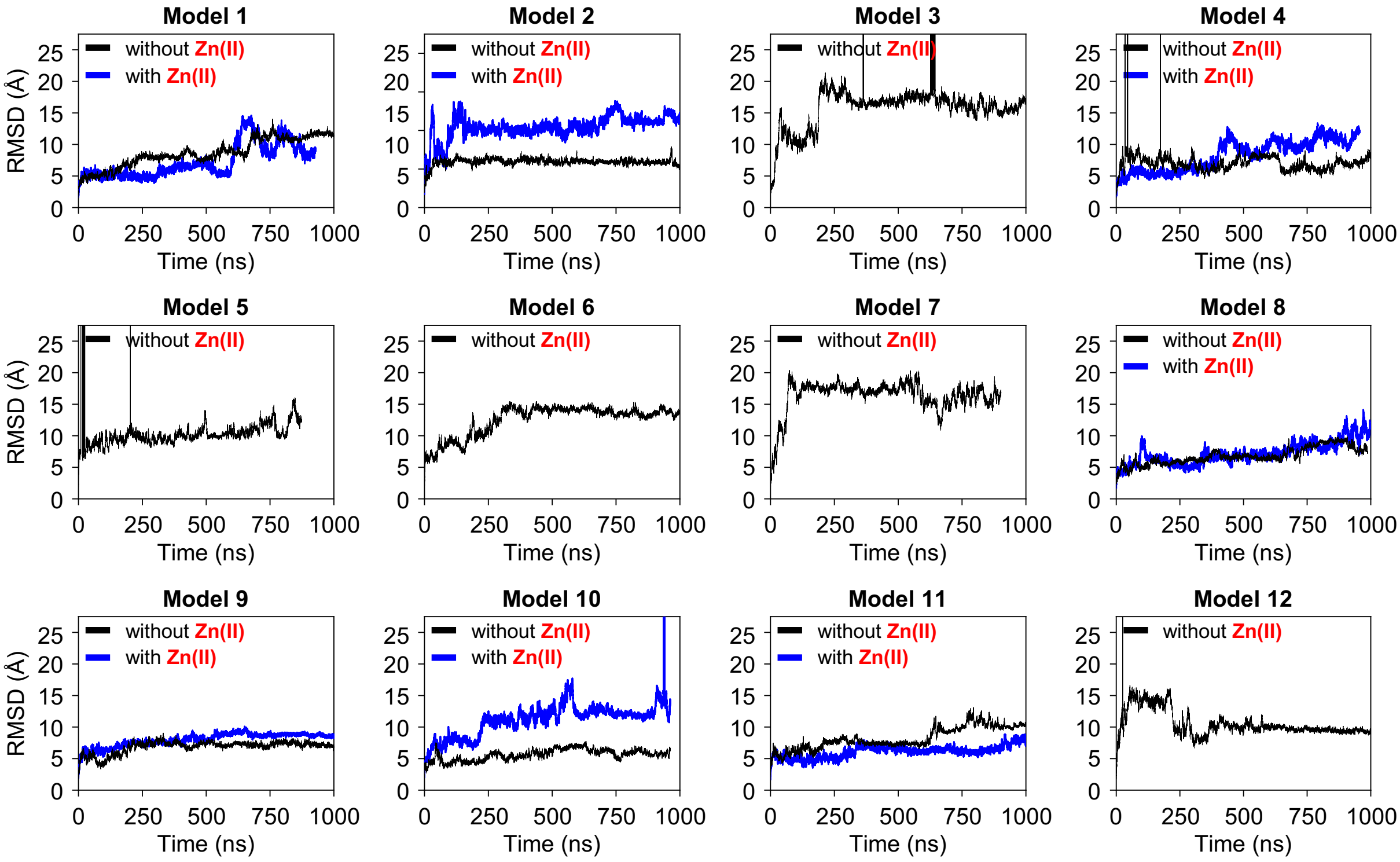

Figure S4. Computed RMSD values of ZF2–4–Aβ40 heterodimers.

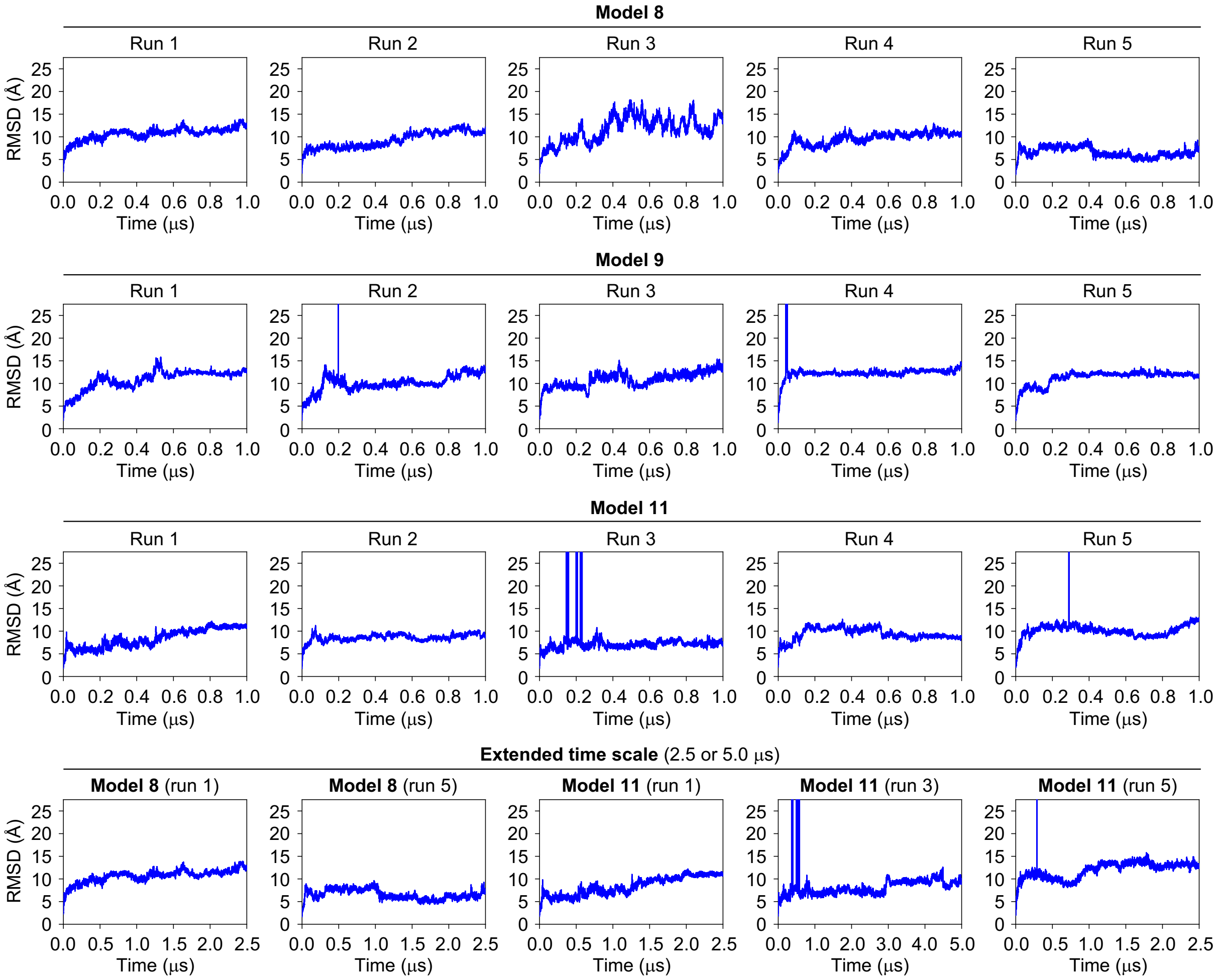

Figure S5. RMSD profiles from the extended MD simulations of selected ZF2–4–Aβ_{40} heterodimer models.

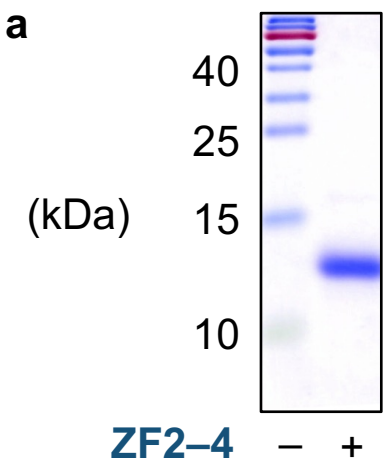

b

Samples	Zn (μg/kg)	Zn (μM)
ZF2–4	3873.7	59.3
H_2O	0.1	2.2×10^{-3}

Figure S6. Characterization of ZF2–4. (a) Purification of ZF2–4 confirmed by SDS–PAGE. Conditions: [ZF2–4] = 40 μM; 25 mM MOPS, pH 7.4, 50 mM NaCl. (b) Amount of Zn(II) in ZF2–4 analyzed by ICP–MS. Conditions: [ZF2–4] = 20 μM; H_2O.

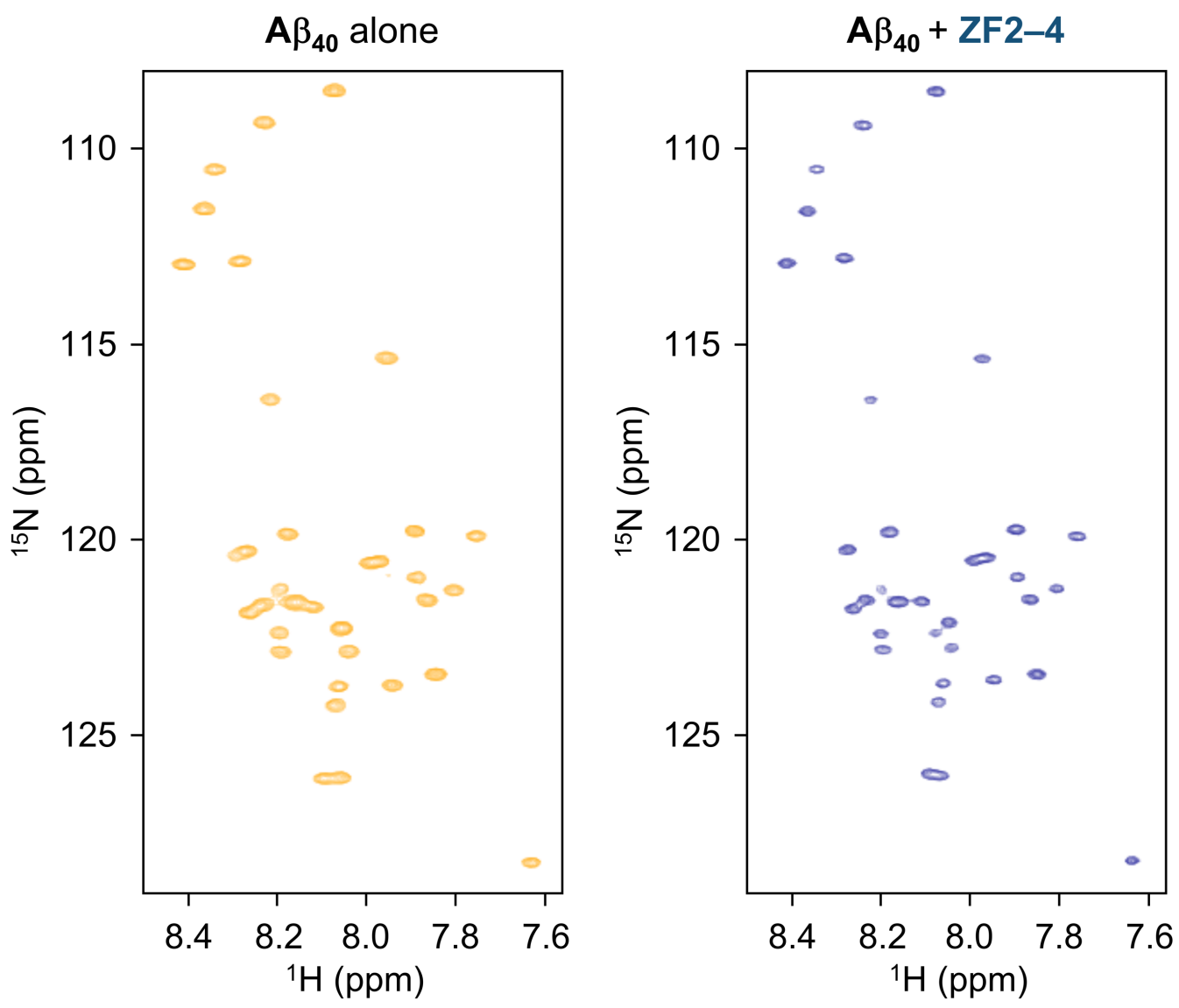

Figure S7. Individual 2D NMR spectra of ^{15}N-labeled Aβ_{40} alone and in the presence of ZF2–4. The corresponding overlaid spectra are shown in Figure 2g. Conditions: [^{15}N-labeled Aβ_{40}] = 20 μM; [ZF2–4] = 200 μM; 20 mM HEPES, pH 7.4, 1 mM DTT, 10% v/v D_2O; 10 °C.

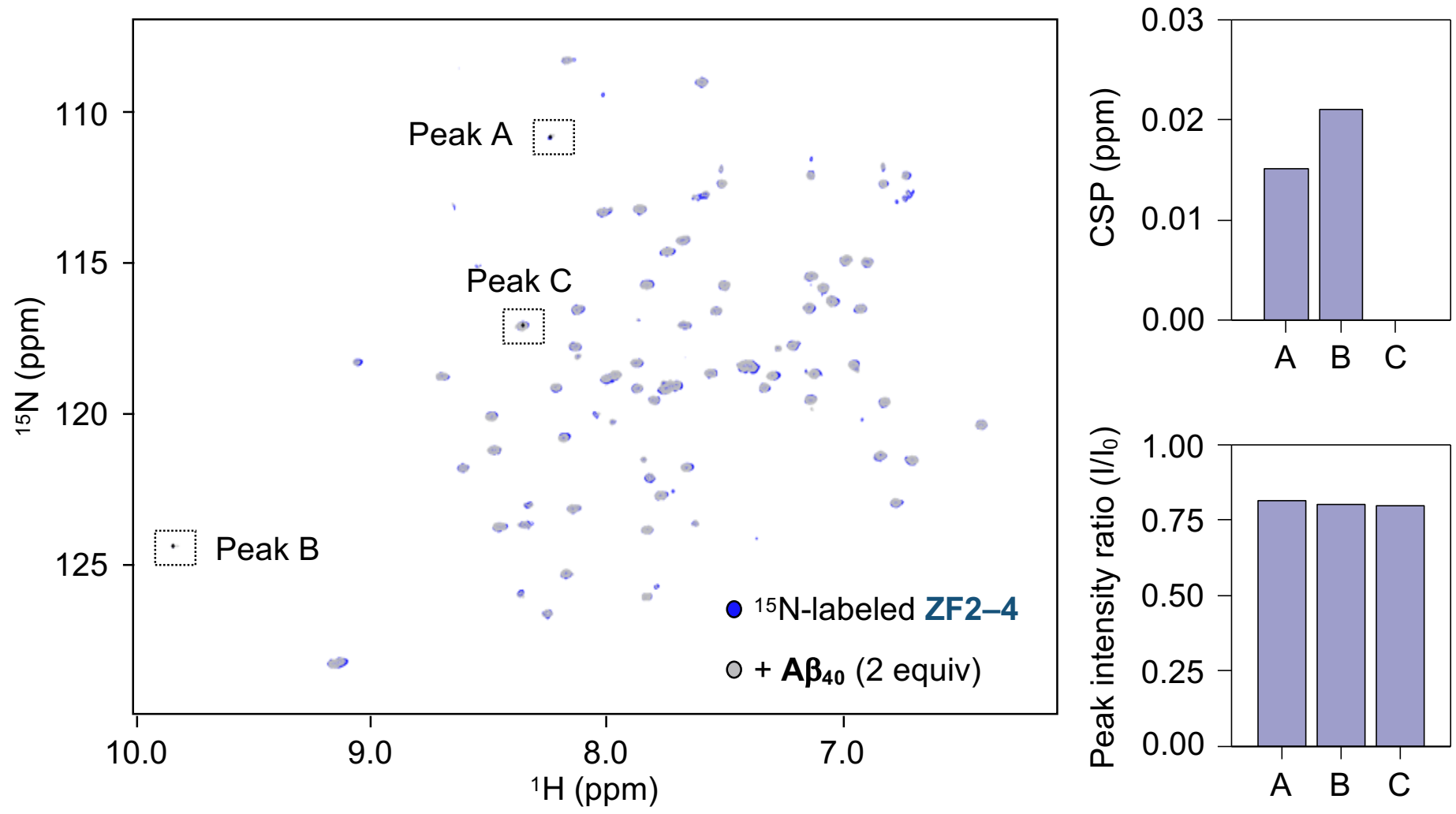

Figure S8. 2D NMR analyses of ^{15}N-labeled ZF2–4 upon treatment of Aβ_{40}. The CSPs and peak intensity ratios of three peaks are depicted as the representative residues that were significantly or not significantly shifted upon addition of Aβ_{40}. Conditions: [^{15}N-labeled ZF2–4] = 50 μM; [Aβ_{40}] = 100 μM; 20 mM HEPES, pH 7.4, 1 mM DTT, 10% v/v D_2O; 10 °C.

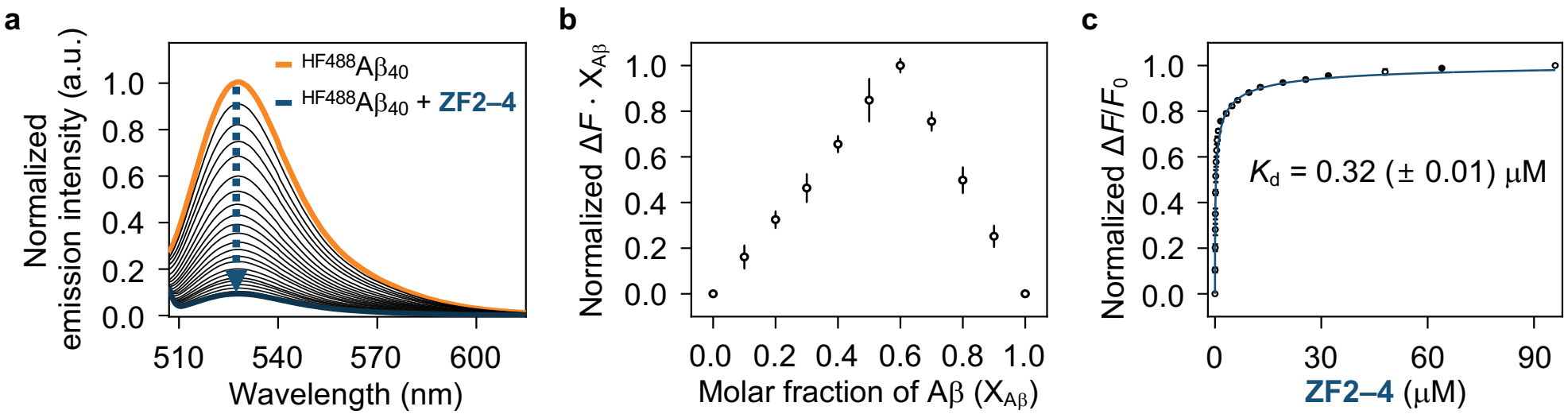

Figure S9. Binding properties of ZF2–4 toward $A\beta_{40}$ measured by fluorescence-quenching experiments. Changes in the fluorescence intensity of $^{HF488}A\beta_{40}$ were monitored upon titration with increasing concentrations of ZF2–4. The K_d value for the ZF2–4–$A\beta_{40}$ complex was determined by fitting the $\Delta F/F_0$ values using the binding stoichiometry estimated by the Job plot. Conditions: (for fluorescence-quenching studies) [$^{HF488}A\beta_{40}$] = 320 nM; [ZF2–4] = 0 to 96 μM; 20 mM HEPES, pH 7.4, 1 mM DTT; room temperature; λ_{ex} = 503 nm; λ_{em} = 507–675 nm; (for Job plot measurements) [$^{HF488}A\beta_{40}$] + [ZF2–4] = 640 nM; 20 mM HEPES, pH 7.4, 1 mM DTT; room temperature; λ_{ex} = 503 nm; λ_{em} = 528 nm. Data are presented as mean ± s.e.m. (standard error of the mean) of three independent experiments.

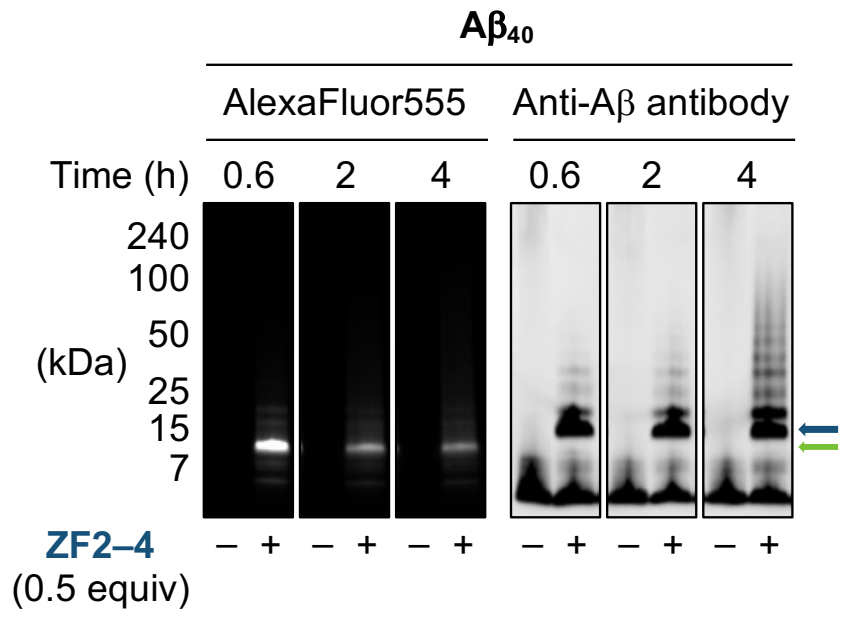

Figure S10. Size distributions of ZF2–4 and Aβ_{40} monitored by tricine–PAGE experiments. The molecular weight distributions of ZF2–4 and Aβ_{40} were visualized by tricine–PAGE with fluorophore-conjugated ZF2–4 and an anti-Aβ antibody (6E10), respectively. The peaks assigned to monomeric ZF2–4 (*ca*. 10.2 kDa) and the ZF2–4–Aβ_{40} adduct in a 1:1 ratio (*ca*. 14.5 kDa) are indicated with green and blue arrows, respectively. The unprocessed gel images are presented in Figure S11. Conditions: [Aβ_{40}] = 100 μM; [^{HF555}ZF2–4 or ZF2–4] = 50 μM; 20 mM HEPES, pH 7.4, 150 mM NaCl, 1 mM DTT; incubation for 0.6, 2, and 4 h; 37 °C; quiescence. All measurements were performed in triplicate.

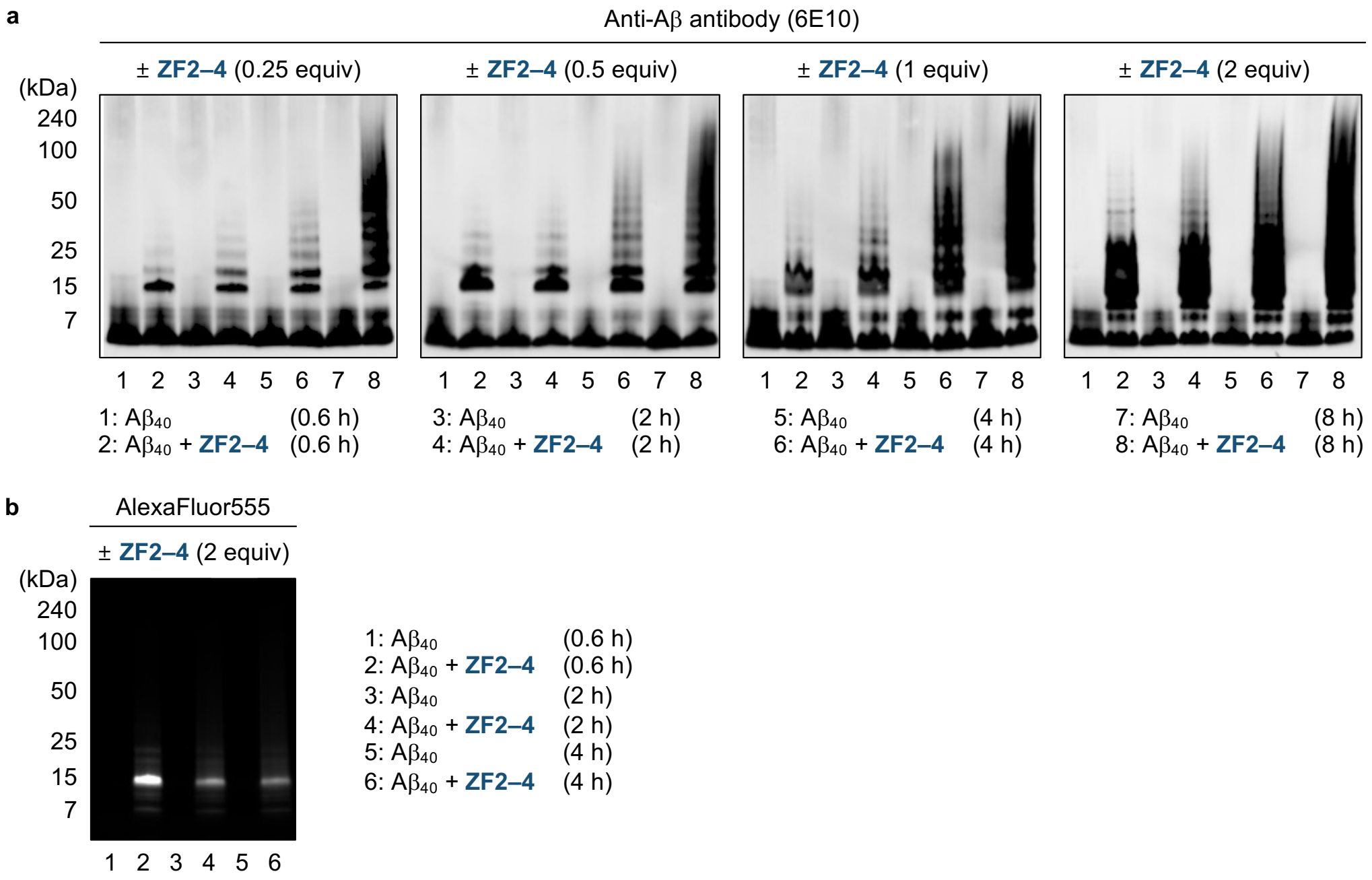

Figure S11. Original tricine–PAGE data from Figure 3f and S10.

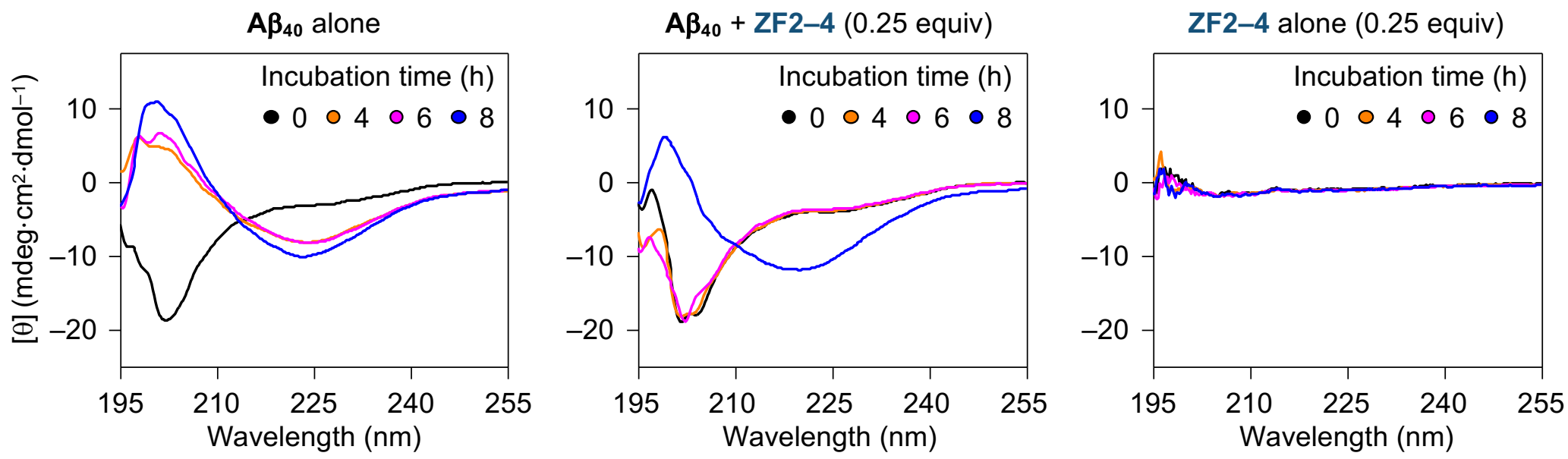

Figure S12. Change in the secondary structure of $A\beta_{40}$ upon aggregation with ZF2–4 analyzed by CD spectroscopy. $A\beta_{40}$ was incubated with 0.25 equiv of ZF2–4 for 0, 4, 6, and 8 h. The signals of $A\beta_{40}$ with ZF2–4 were obtained by subtracting the features of ZF2–4 under the same conditions. Conditions: [$A\beta_{40}$] = 100 μM; [ZF2–4] = 25 μM; 20 mM HEPES, pH 7.4, 150 mM NaF, 1 mM DTT; incubation for 0, 4, 6, and 8 h; 37 °C; quiescence. All measurements were performed in triplicate.

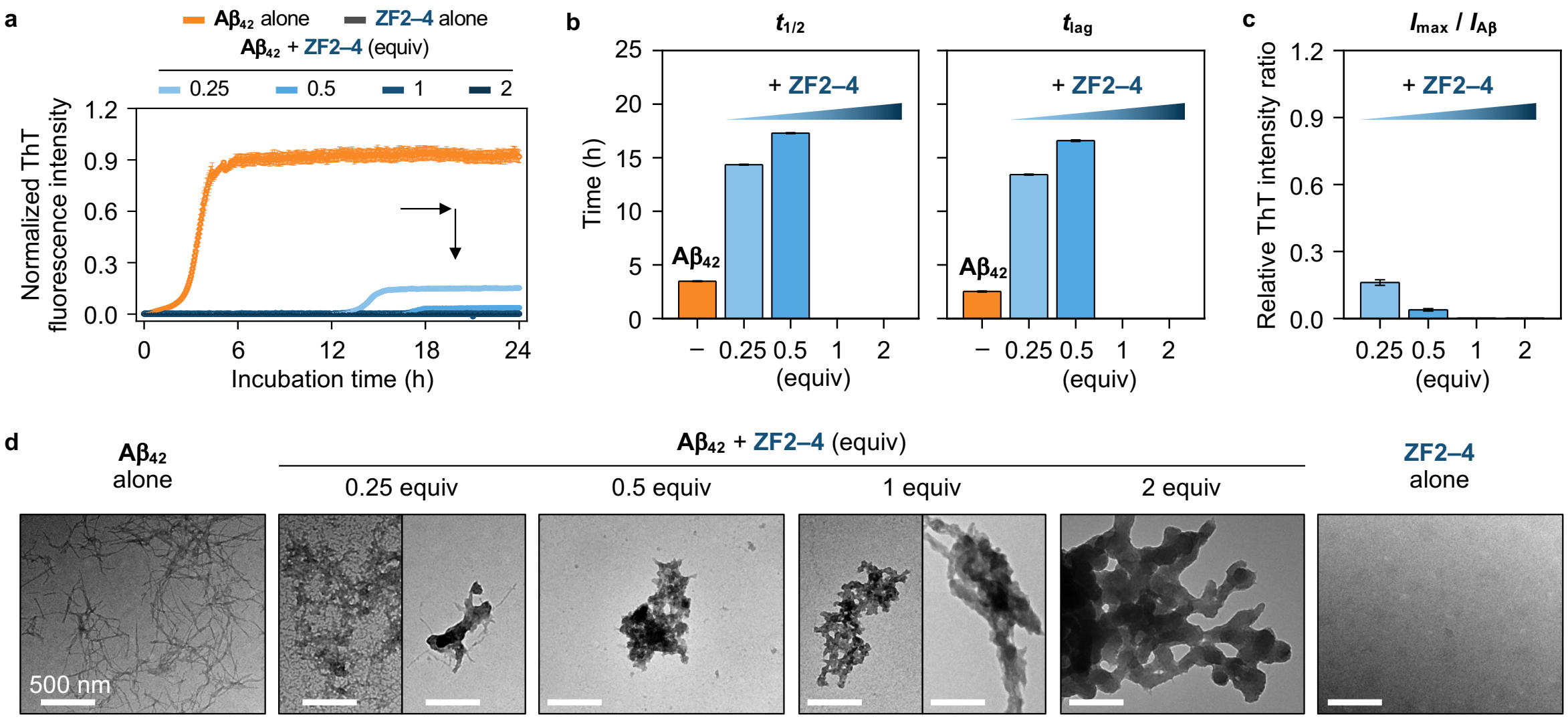

Figure S13. Impact of ZF2–4 on the aggregation of Aβ_{42}. (a–c) Formation of β-sheet-rich aggregates in the samples of Aβ_{42} incubated with and without ZF2–4 monitored by the ThT fluorescence assay. The normalized fluorescence intensity was plotted as a function of incubation time and fitted to calculate kinetic parameters. Conditions: [Aβ_{42}] = 20 μM; [ZF2–4] = 5, 10, 20, and 40 μM; [ThT] = 20 μM; 20 mM HEPES, pH 7.4, 150 mM NaCl, 1 mM DTT; 37 °C; quiescence; λ_{ex} = 440 nm; λ_{em} = 490 nm. Data are presented as mean ± s.e.m. of three independent experiments. (d) Morphologies of the resultant Aβ_{42} aggregates generated with and without ZF2–4 visualized by TEM. Conditions: [Aβ_{42}] = 100 μM; [ZF2–4] = 25, 50, 100, and 200 μM; 20 mM HEPES, pH 7.4, 150 mM NaCl, 1 mM DTT; 37 °C; quiescence. Scale bar = 500 nm.

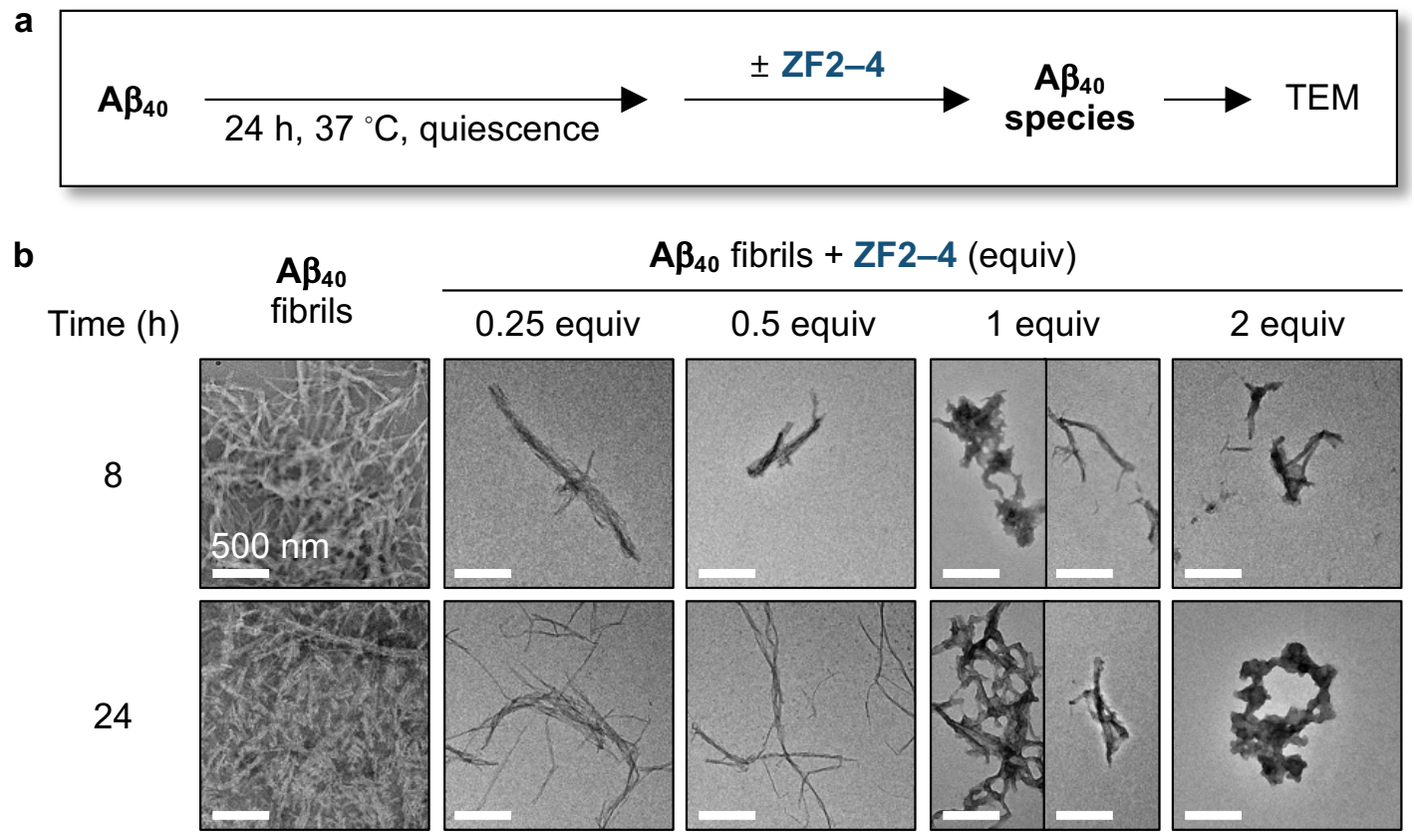

Figure S14. Effect of ZF2–4 on preformed $A\beta_{40}$ aggregates. (a) Scheme of the disaggregation studies. (b) Morphologies of the resultant aggregates monitored by TEM. Preformed $A\beta_{40}$ aggregates produced by incubation for 24 h were treated with different concentrations of ZF2–4 followed by additional incubation for 8 h or 24 h. Conditions: $[A\beta_{40}]$ = 100 μM; [ZF2–4] = 25, 50, 100, and 200 μM; 20 mM HEPES, pH 7.4, 150 mM NaCl, 1 mM DTT; 37 °C; quiescence. Scale bar = 500 nm.

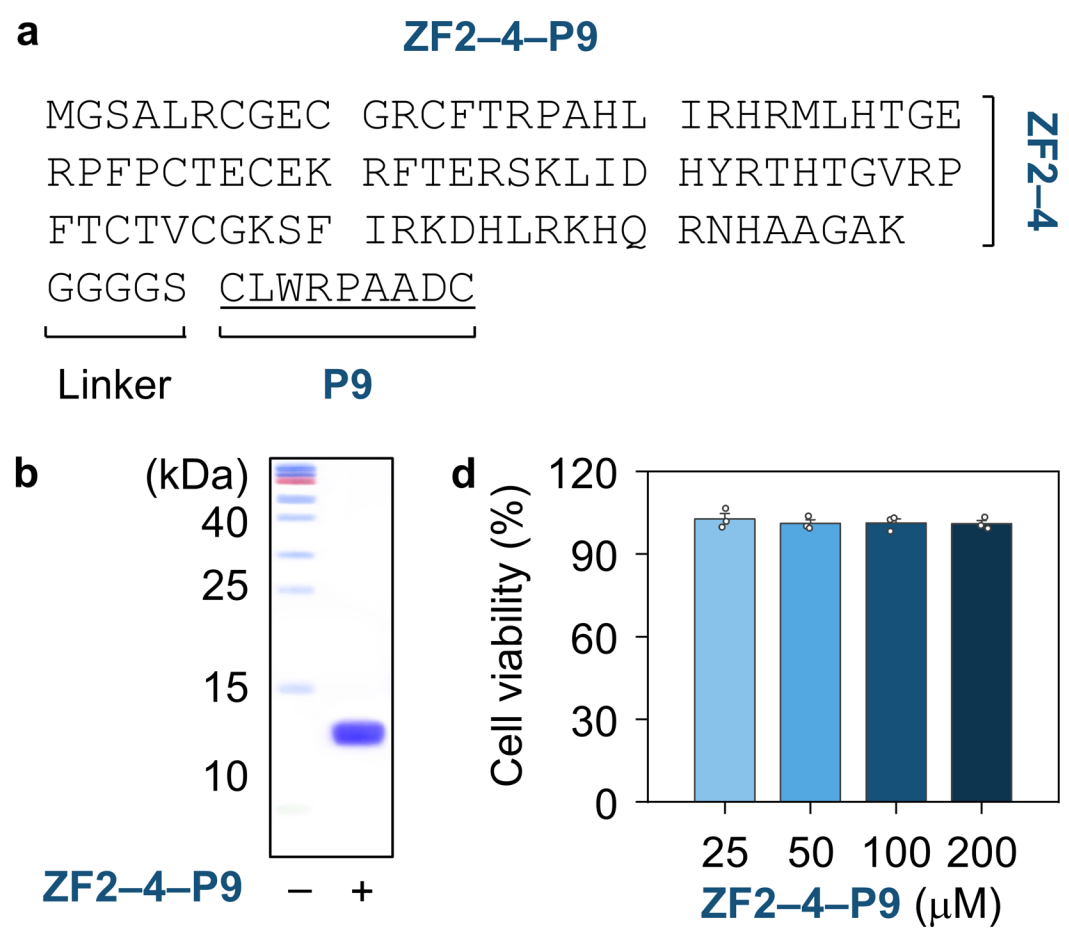

c

	ZF2–4	ZF2–4–P9
$-\log P_e$	5.79	5.00
CNS(+/–)	CNS–	CNS+

Figure S15. Preparation and characterization of ZF2–4–P9. (a) Amino acid sequence of ZF2–4–P9. The P9 motif at the *C*-terminus is underlined. (b) Purification of ZF2–4–P9 confirmed by SDS–PAGE. Conditions: [ZF2–4–P9] = 40 μM; 25 mM MOPS, pH 7.4, 50 mM NaCl. (c) BBB permeability of ZF2–4 and ZF2–4–P9 evaluated using the PAMPA–BBB. A $-\log P_e$ value below 5.4 corresponds to CNS+ and above 5.7 to CNS–. Conditions: [ZF2–4–P9] = 100 μM; Prisma HT, pH 7.4. (d) Cytotoxicity of ZF2–4–P9 determined by the MTT assay. Cell viability was determined relative to cells treated with an equivalent volume of the corresponding buffered solution. Conditions: [ZF2–4–P9] = 25, 50, 100, and 200 μM; 20 mM HEPES, pH 7.4, 150 mM NaCl; 48 h incubation; 37 °C; quiescence conditions; SH-SY5Y cells that were non-differentiated. Data are presented as mean ± s.e.m. of three independent experiments.

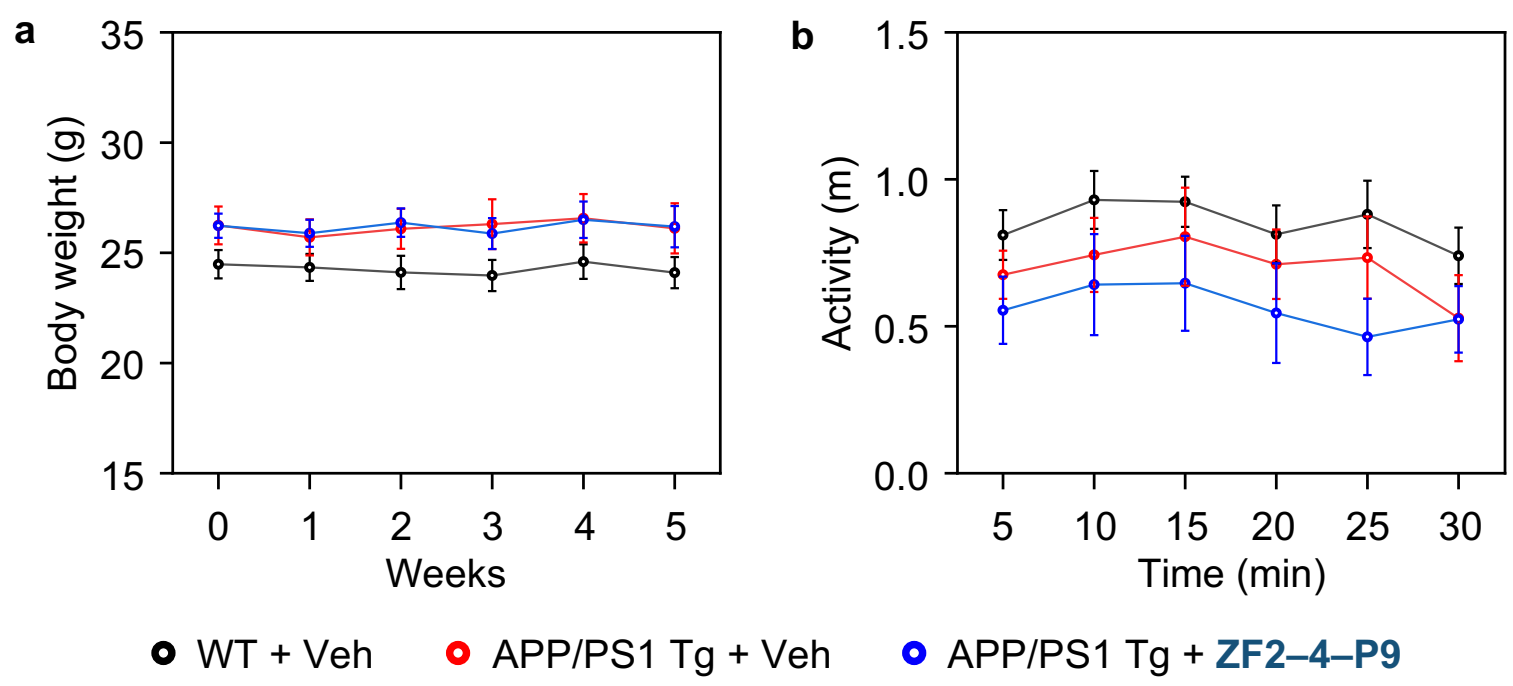

Figure S16. Effects of ZF2–4–P9 on the body weight and locomotor activity in APP/PS1 Tg mice. (a) Change in the body weight of WT and APP/PS1 Tg mice throughout the treatment of Veh and ZF2–4–P9. Animal number: *n* = 10 for Veh-treated WT mice; *n* = 9 for Veh-treated APP/PS1 Tg mice; *n* = 7 for ZF2–4–P9-treated APP/PS1 Tg mice. Data are presented as mean ± s.e.m. (b) Measurement of the distance traveled in 5 min intervals explored over a 30 min period during the open field test. Animal number: *n* = 10 for Veh-treated WT mice; *n* = 7 for Veh-treated APP/PS1 Tg mice; *n* = 7 for ZF2–4–P9-treated APP/PS1 Tg mice. Data are presented as mean ± s.e.m.

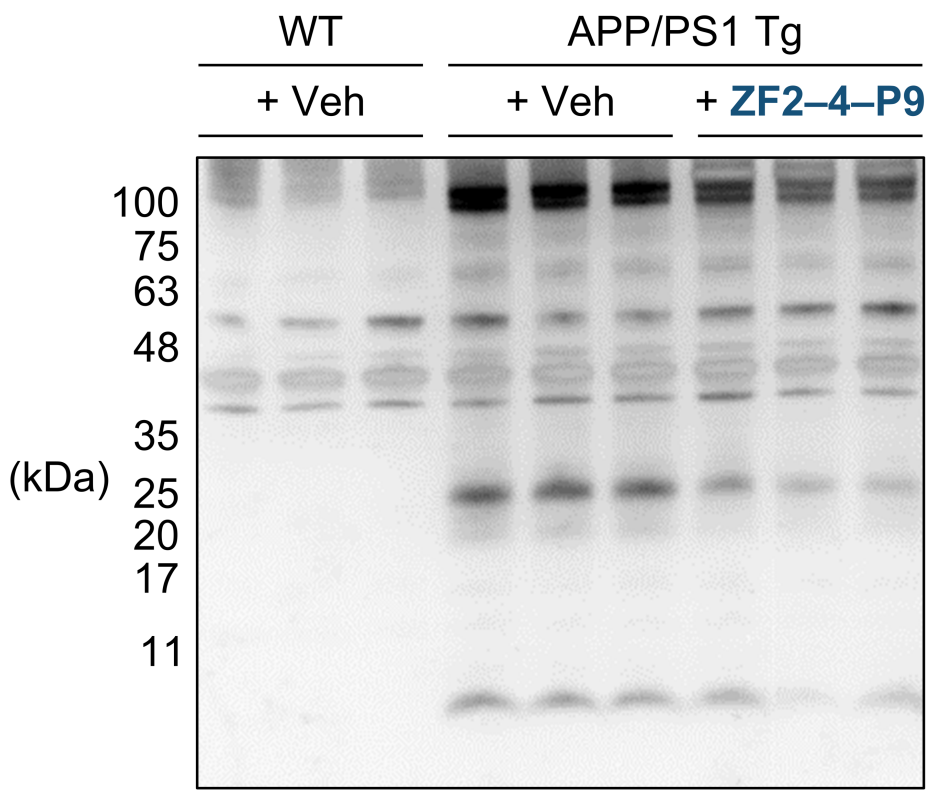

Figure S17. Influence of ZF2–4–P9 administration on the molecular weight distribution of Aβ species in brain homogenates from APP/PS1 Tg mice. Following centrifugation of brain homogenates, the resulting pellets were resuspended in 20 mM Tris, pH 7.4 containing 2% SDS and centrifuged again. The resulting supernatants, corresponding to the SDS-soluble fractions, were subjected to gel/Western blot analysis using an anti-Aβ antibody (6E10).

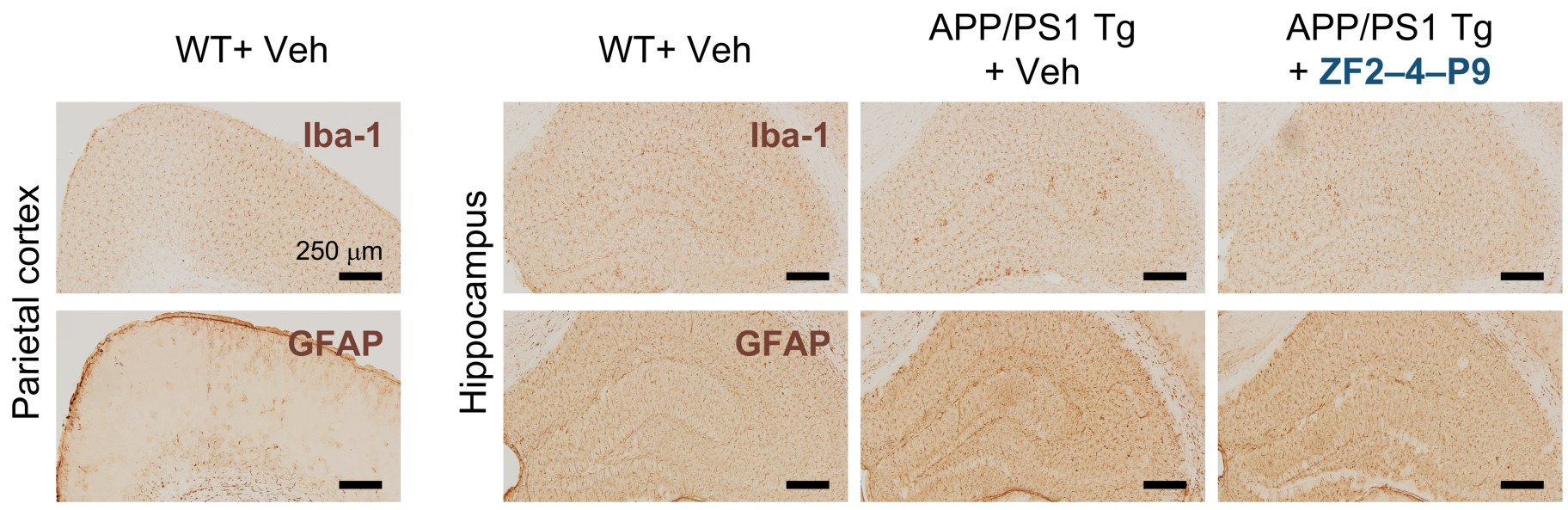

Figure S18. Representative images visualized by immunostaining with anti-Iba-1 and anti-GFAP antibodies. Scale bar = 250 μm.

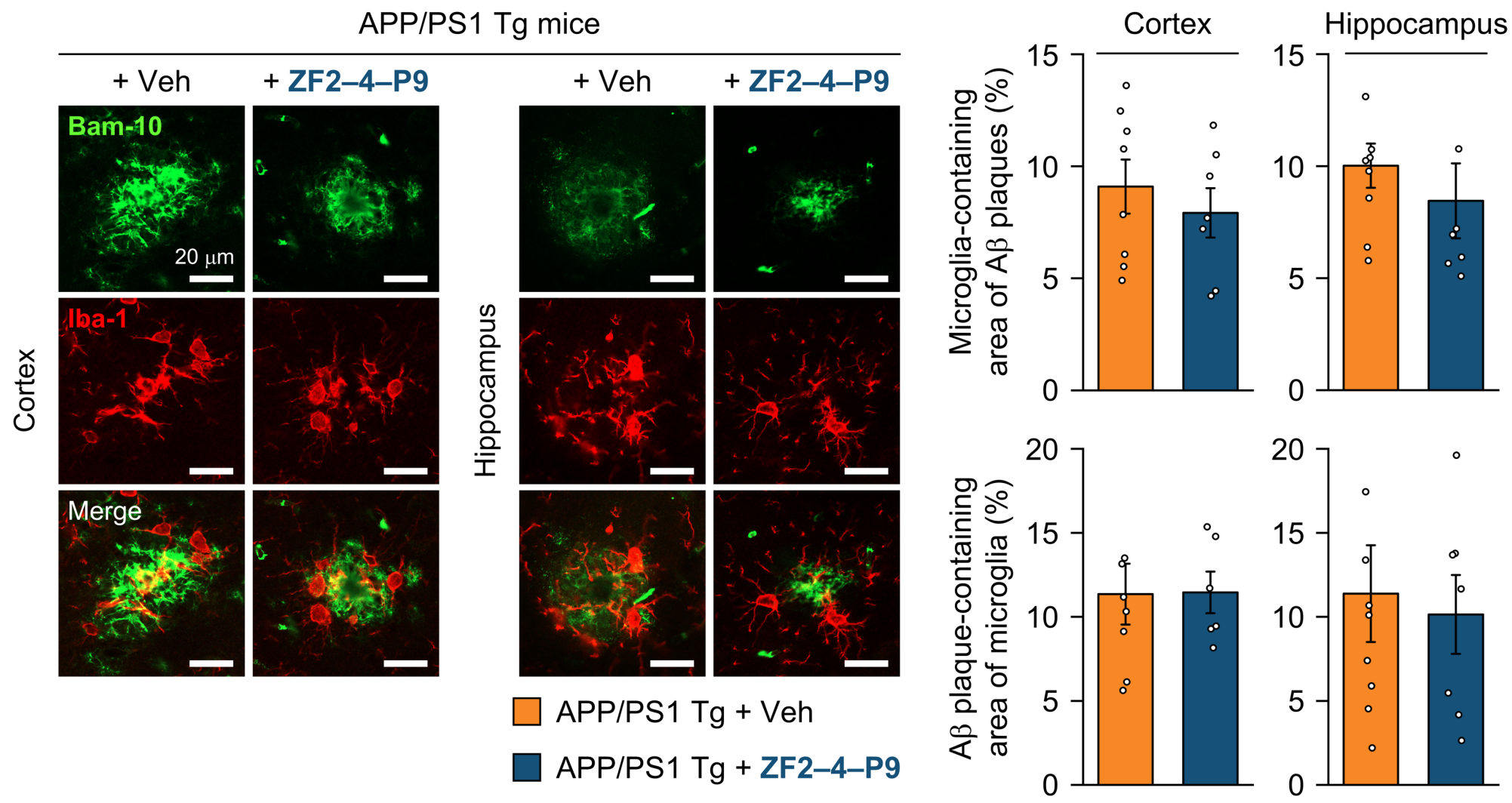

Figure S19. Quantitative analysis of the spatial association between Aβ plaques and microglia in the parietal cortices and hippocampi of Veh-treated APP/PS1 Tg and ZF2–4–P9-treated APP/PS1 Tg mice. Representative images obtained by co-immunostaining with anti-Aβ (Bam-10) and anti-Iba1 antibodies were shown. Scale bar = 20 μm. Animal number: n = 9 for Veh-treated APP/PS1 Tg mice; n = 7 for ZF2–4–P9-treated APP/PS1 Tg mice. Data are presented as mean ± s.e.m.

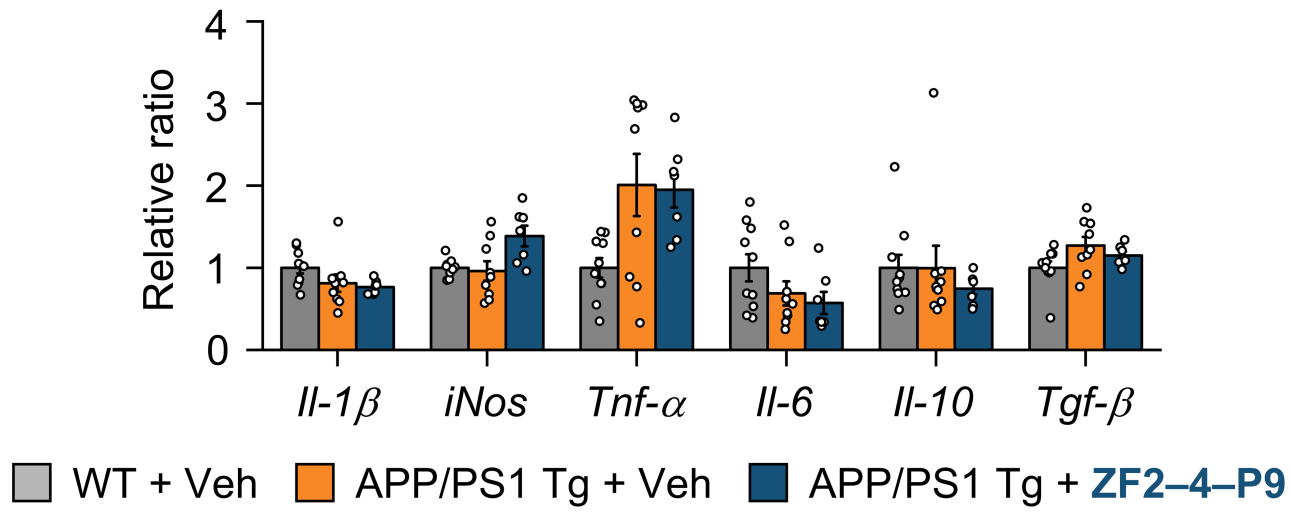

Figure S20. mRNA expression levels of inflammatory cytokines in the microglia isolated from the cortices following treatment with Veh or ZF2–4–P9 in WT and APP/PS1 Tg mice. Proinflammatory marker: *Il-1β*, *iNos*, *Tnf-α*, and *Il-6*; immunoregulatory cytokine: *Il-10*; antiinflammatory marker: *Tgf-β*. Data are presented as mean ± s.e.m.

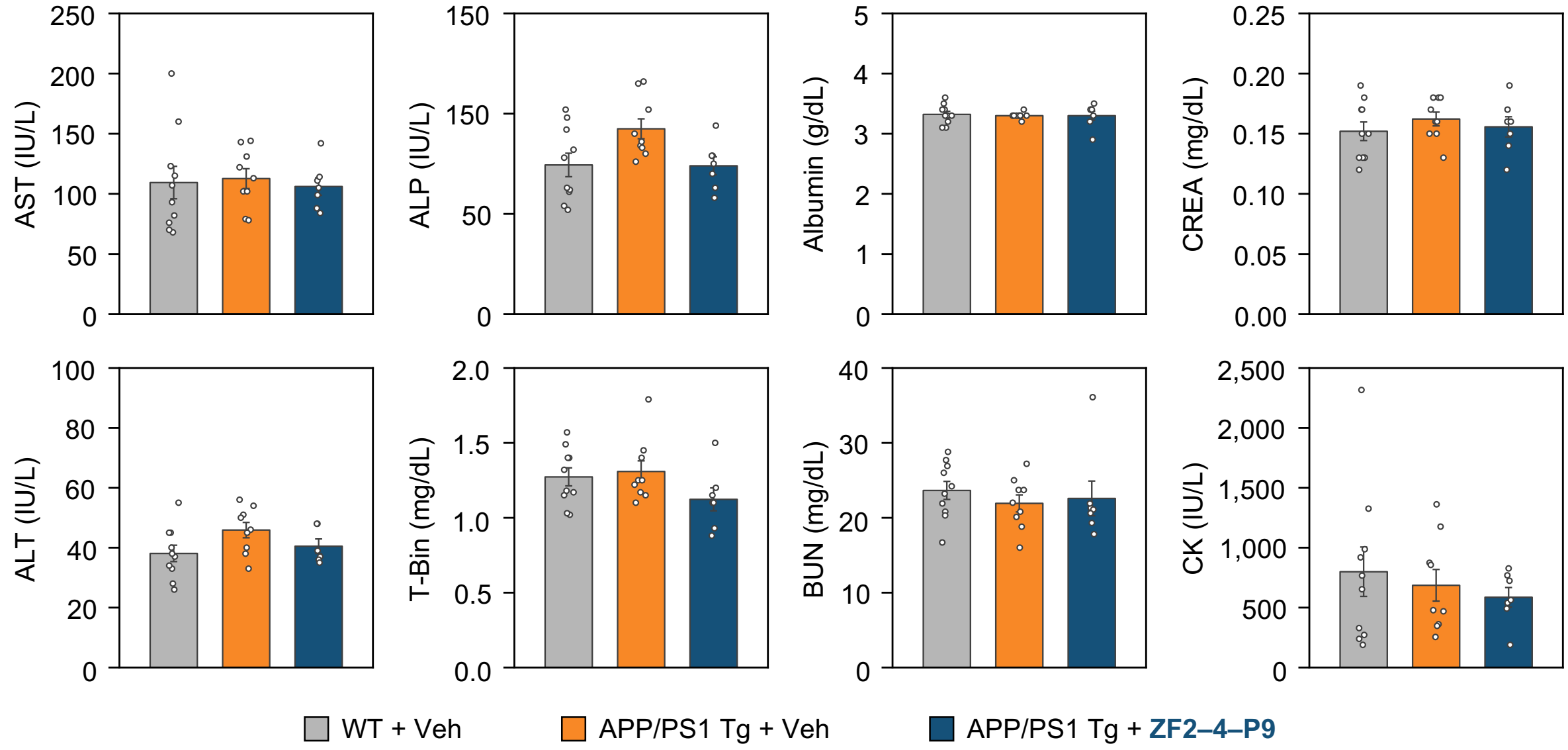

Figure S21. Blood chemistry analysis for the levels of AST, ALP, albumin, CREA, ALT, T-Bin, BUN, and CK. Animal number: *n* = 10 for Veh-treated WT mice; *n* = 9 for Veh-treated APP/PS1 Tg mice; *n* = 7 for ZF2–4–P9-treated APP/PS1 Tg mice. Data are presented as mean ± s.e.m.

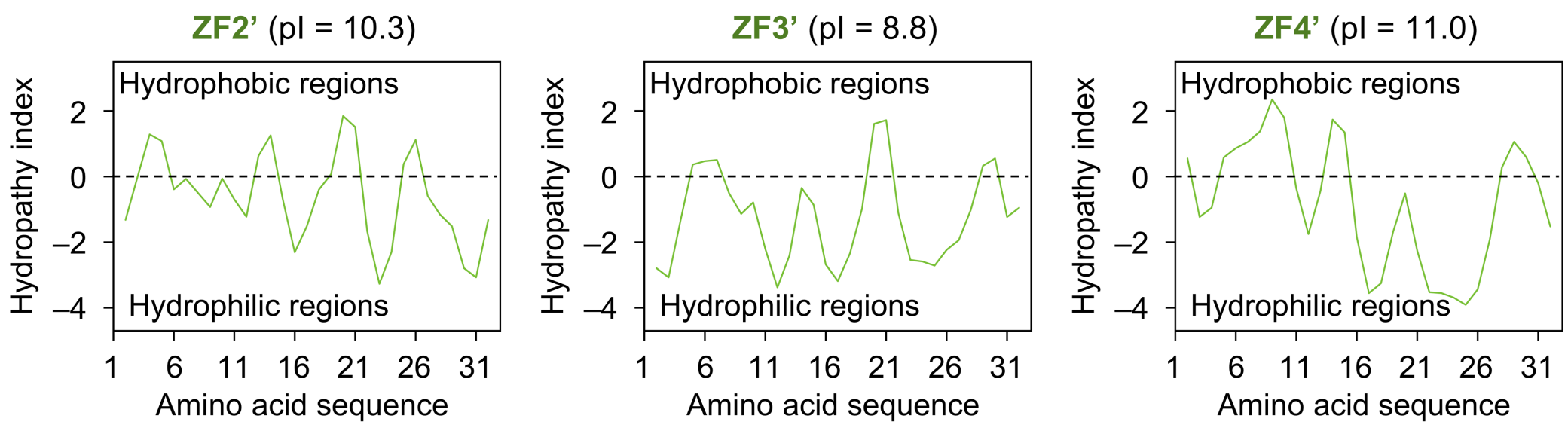

Figure S22. Physicochemical properties of ZF2', ZF3', and ZF4'. Hydrophilicity and hydrophobicity profiles were calculated using the ExPASy server.

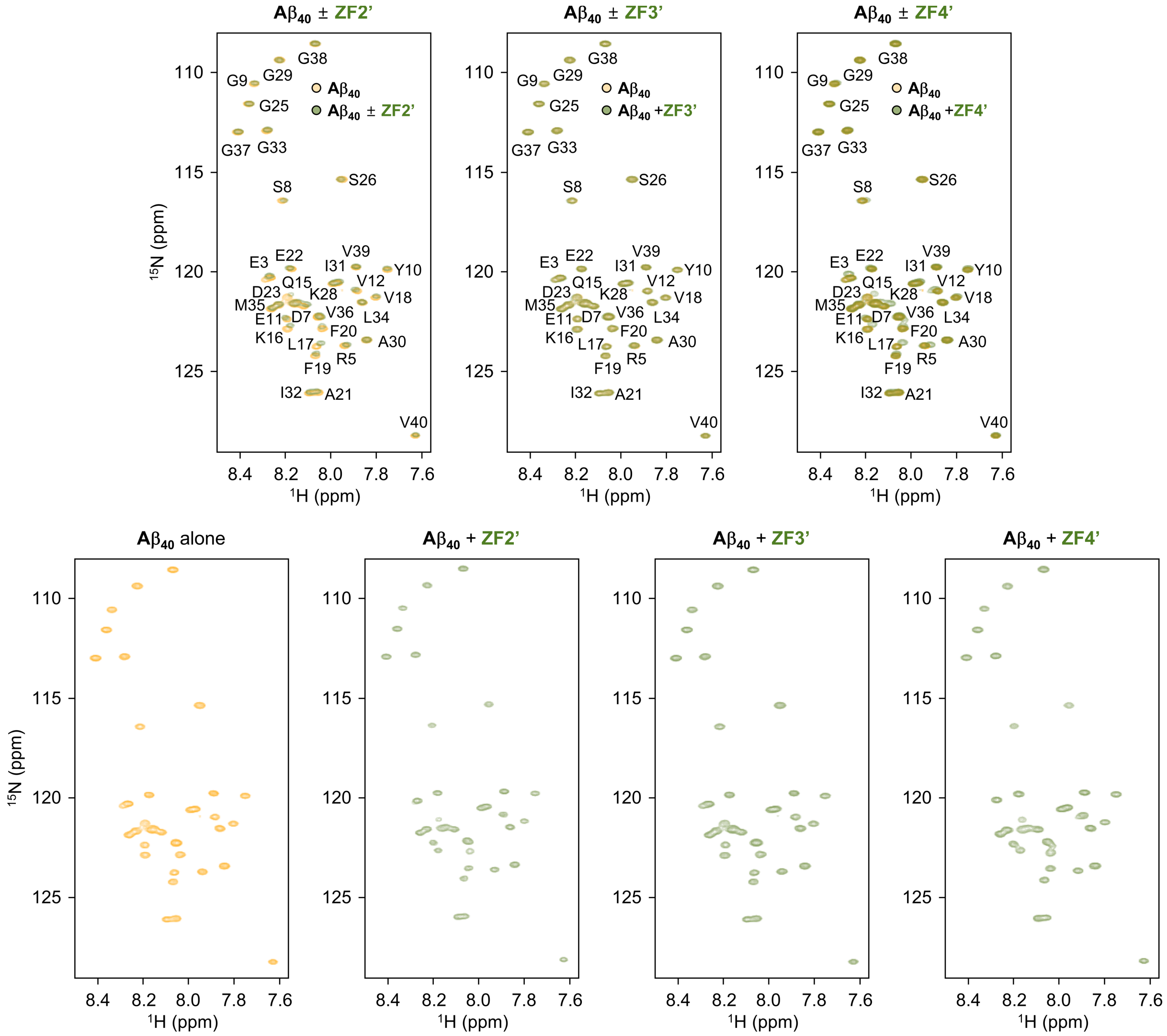

Figure S23. Individual 2D NMR spectra of ^{15}N-labeled Aβ_{40} in the absence and presence of ZFx'. Conditions: [^{15}N-labeled Aβ_{40}] = 20 μM; [ZFx'] = 200 μM; 20 mM HEPES, pH 7.4, 1 mM DTT, 10% v/v D_2O; 10 °C.

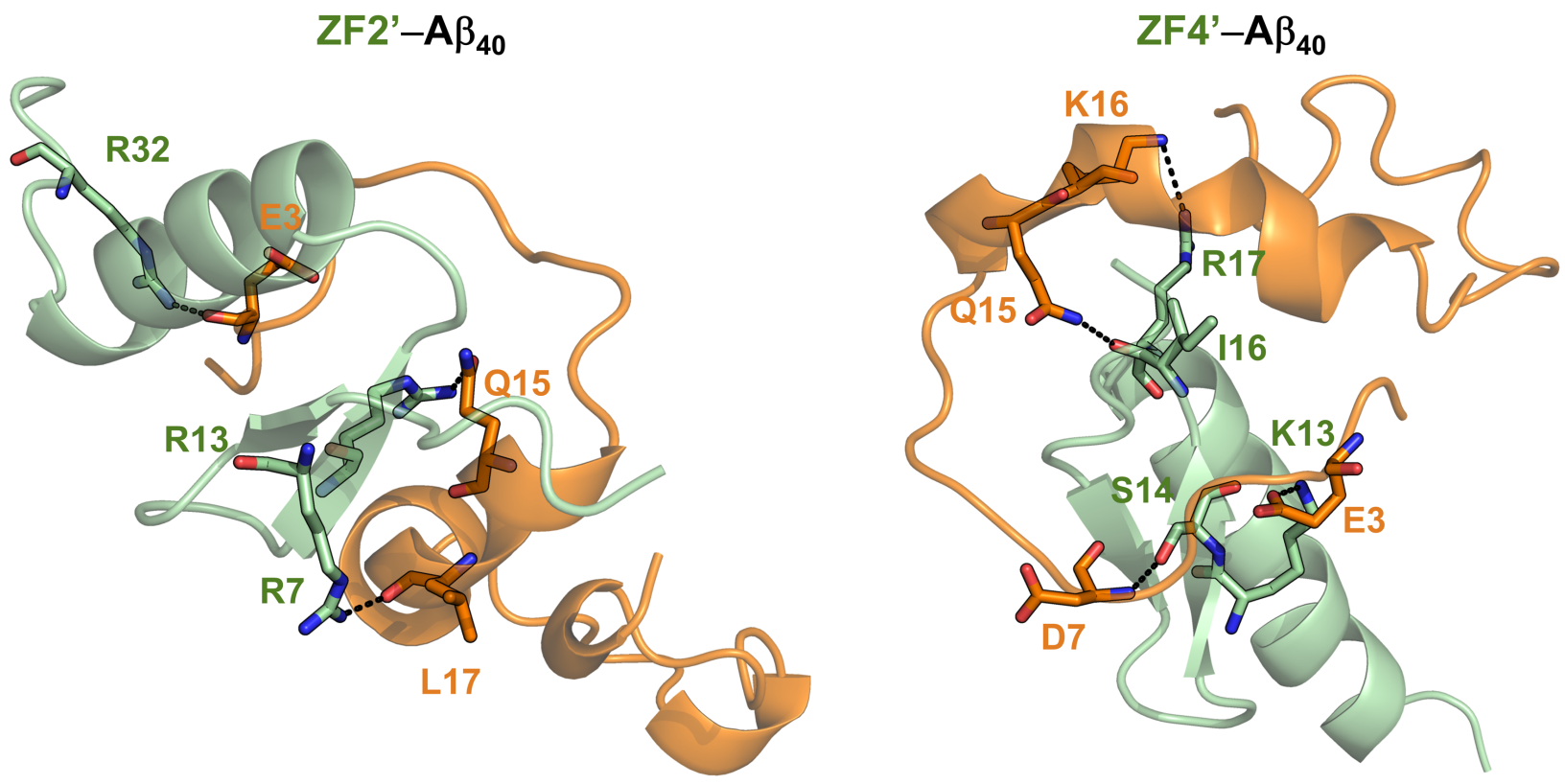

Figure S24. Structural analysis of the heterodimeric ZF2'–Aβ_{40} and ZF4'–Aβ_{40} complexes obtained from molecular docking simulations. ZFx' and Aβ_{40} are shown in green and orange, respectively. Possible hydrogen bonds within 3.0 Å are indicated by black dashed lines. The corresponding monomeric structures of ZFx' and Aβ_{40} are presented in Figure 6c and S1b, respectively.

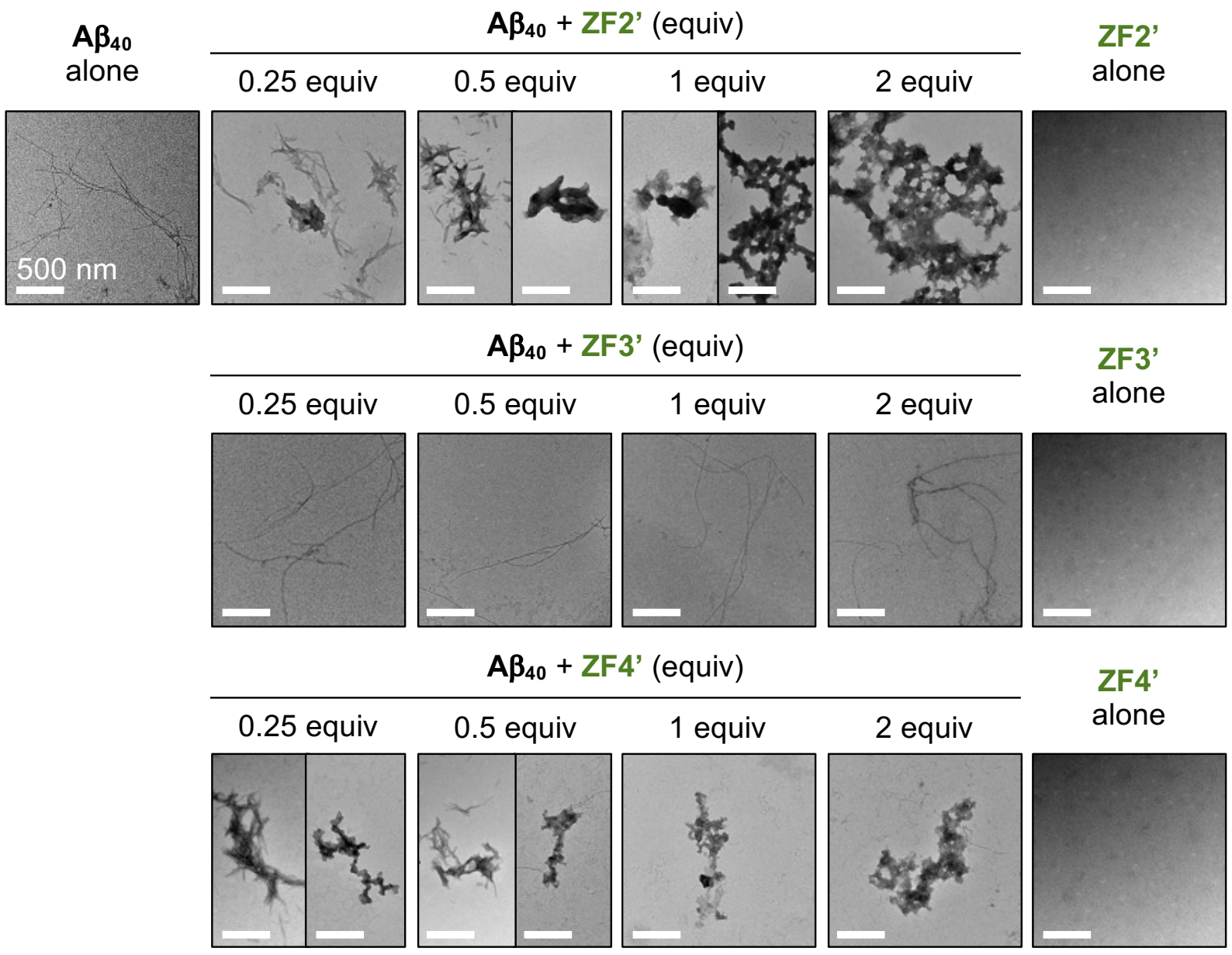

Figure S25. Morphologies of the resultant $A\beta_{40}$ aggregates with and without ZFx' visualized by TEM. Conditions: $[A\beta_{40}]$ = 100 μM; [apo-ZFx'] = 25, 50, 100, and 200 μM; [Zn(II)] = 22.5, 45, 90, and 180 μM; 20 mM HEPES, pH 7.4, 150 mM NaCl, 1 mM DTT; 37 °C; quiescence. It should be noted that 0.9 equiv of Zn(II) was added to apo-ZFx' and incubated for 3 min to minimize free Zn(II) in solution. Scale bar = 500 nm.

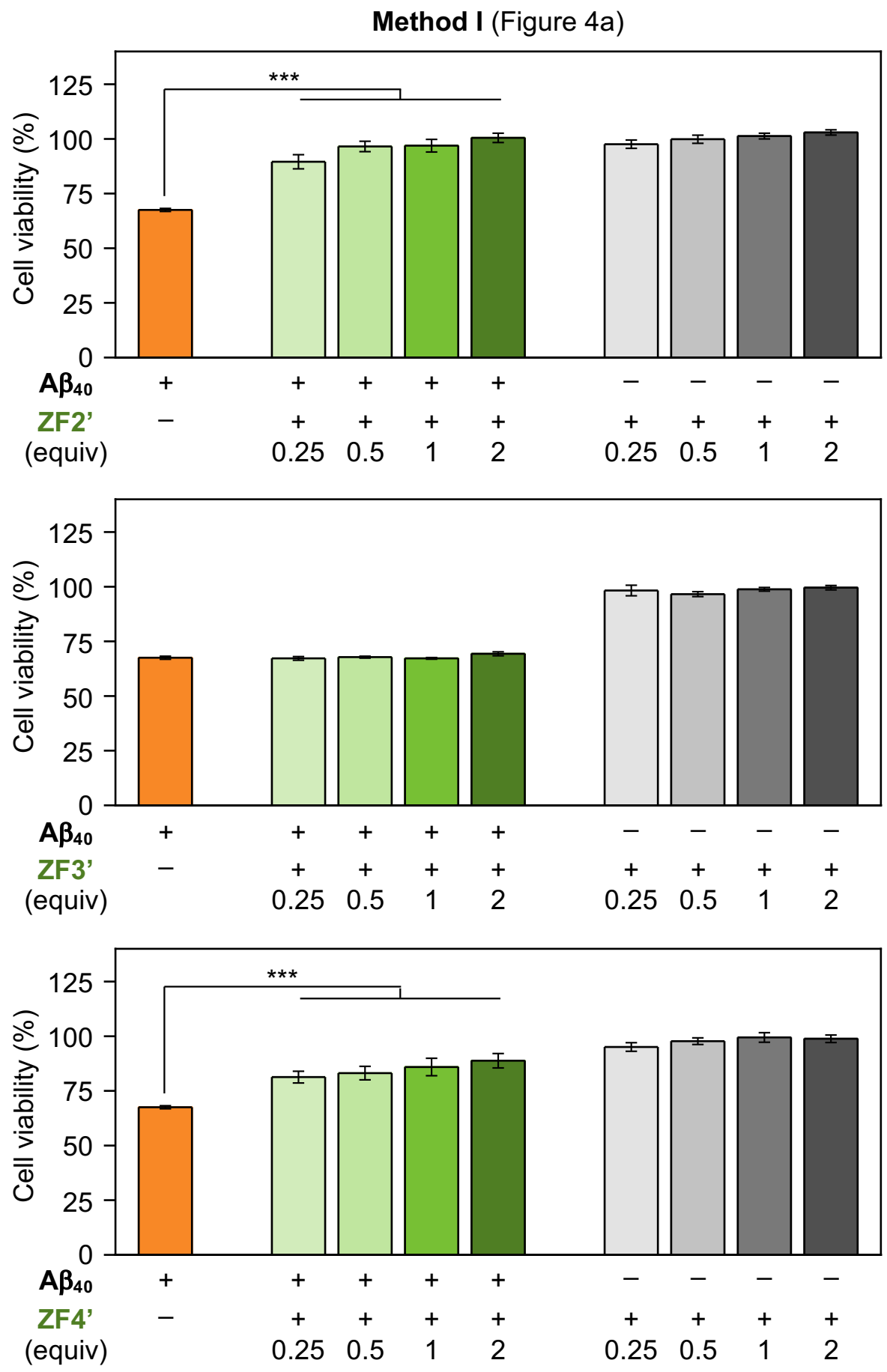

Figure S26. Influence of ZFx' on the cytotoxicity of Aβ_{40}. The viability (%) of cells treated with either Aβ_{40}, ZFx', or both was assessed using the MTT assay. Cell survival was calculated in comparison to that with an equivalent amount of the buffered solution. Conditions: [Aβ_{40}] = 10 µM; [apo-ZFx'] = 2.5, 5, 10, and 20 µM; [Zn(II)] = 2.25, 4.5, 9, and 18 µM; 20 mM HEPES, pH 7.4, 150 mM NaCl; pre- and post-incubation for 8 h and 48 h, respectively; 37 °C; quiescence; SH-SY5Y cells that were non-differentiated. It should be noted that 0.9 equiv of Zn(II) was added to apo-ZFx' and incubated for 3 min to minimize free Zn(II) in solution. ***P < 0.001; n = 3; Student's t-test. Data are presented as mean ± s.e.m.

a

Series I		
	Amino acid sequences	β-strand moieties
**M1**	RDGSA LRCGE CEKRF TRPAH LIRHR MLHTG ERPFP	X
**M2**	RDGSA LRCGE CGKSF TRPAH LIRHR MLHTG ERPFP	O
**M3**	RDGSA LPCGE CGRCF TRPAH LIRHR MLHTG ERPFP	O
**M4**	RDGSA LPCGE CEKRF TRPAH LIRHR MLHTG ERPFP	X
**M5**	[1]DGSA LPCGE CGKSF TRPAH LIRHR MLHTG ERPFP	O
**M6**	RDGSA LTCGE CGRCF TRPAH LIRHR MLHTG ERPFP	O
**M7**	RDGSA LTCGE CEKRF TRPAH LIRHR MLHTG ERPFP	X
**M8**	RDGSA LTCGE CGKSF TRPAH LIRHR MLHTG ERPFP	O
**M9**	RDGSA FRCGE CGRCF TRPAH LIRHR MLHTG ERPFP	O
**M10**	RDGSA FRCGE CEKRF TRPAH LIRHR MLHTG ERPFP	X
**M11**	RDGSA FRCGE CGKSF TRPAH LIRHR MLHTG ERPFP	O
**M12**	RDGSA FPCGE CGRCF TRPAH LIRHR MLHTG ERPFP	O
**M13**	RDGSA FPCGE CEKRF TRPAH LIRHR MLHTG ERPFP	X
**M14**	RDGSA FPCGE CGKSF TRPAH LIRHR MLHTG ERPFP	O
**M15**	RDGSA FTCGE CGRCF TRPAH LIRHR MLHTG ERPFP	O
**M16**	RDGSA FTCGE CEKRF TRPAH LIRHR MLHTG ERPFP	X
**M17**	RDGSA FTCGE CGKSF TRPAH LIRHR MLHTG ERPFP	O

b

Series II		
	Amino acid sequences	β-strand moieties
**M18**	RDGSA LRCGE CGRRF TRPAH LIRHR MLHTG ERPFP	O
**M19**	RDGSA FTCGE CGRRF TRPAH LIRHR MLHTG ERPFP	O
**M20**	RDGSA FPCGE CGRRF TRPAH LIRHR MLHTG ERPFP	O
**M21**	RDGSA LRCGE CGRSF TRPAH LIRHR MLHTG ERPFP	O
**M22**	RDGSA FPCGE CGRSF TRPAH LIRHR MLHTG ERPFP	O
**M23**	RDGSA FTCGE CGRSF TRPAH LIRHR MLHTG ERPFP	O
**M24**	RDGSA LRCGE CGKCF TRPAH LIRHR MLHTG ERPFP	O
**M25**	RDGSA FPCGE CGKCF TRPAH LIRHR MLHTG ERPFP	O
**M26**	RDGSA FTCGE CGKCF TRPAH LIRHR MLHTG ERPFP	O
**M27**	RDGSA LRCGE CGKRF TRPAH LIRHR MLHTG ERPFP	O
**M28**	RDGSA FPCGE CGKRF TRPAH LIRHR MLHTG ERPFP	O
**M29**	RDGSA FTCGE CGKRF TRPAH LIRHR MLHTG ERPFP	O
**M30**	RDGSA LRCGE CERCF TRPAH LIRHR MLHTG ERPFP	X
**M31**	RDGSA FPCGE CERCF TRPAH LIRHR MLHTG ERPFP	X
**M32**	RDGSA FTCGE CERCF TRPAH LIRHR MLHTG ERPFP	O
**M33**	RDGSA LRCGE CERRF TRPAH LIRHR MLHTG ERPFP	X
**M34**	RDGSA FPCGE CERRF TRPAH LIRHR MLHTG ERPFP	X
**M35**	RDGSA FTCGE CERRF TRPAH LIRHR MLHTG ERPFP	X
**M36**	RDGSA LRCGE CERSF TRPAH LIRHR MLHTG ERPFP	X
**M37**	RDGSA FPCGE CERSF TRPAH LIRHR MLHTG ERPFP	X
**M38**	RDGSA FTCGE CERSF TRPAH LIRHR MLHTG ERPFP	O
**M39**	RDGSA LRCGE CEKCF TRPAH LIRHR MLHTG ERPFP	X
**M40**	RDGSA FPCGE CEKCF TRPAH LIRHR MLHTG ERPFP	X
**M41**	RDGSA FTCGE CEKCF TRPAH LIRHR MLHTG ERPFP	O
**M42**	RDGSA LRCGE CEKSF TRPAH LIRHR MLHTG ERPFP	X
**M43**	RDGSA FPCGE CEKSF TRPAH LIRHR MLHTG ERPFP	X
**M44**	RDGSA FTCGE CEKSF TRPAH LIRHR MLHTG ERPFP	X

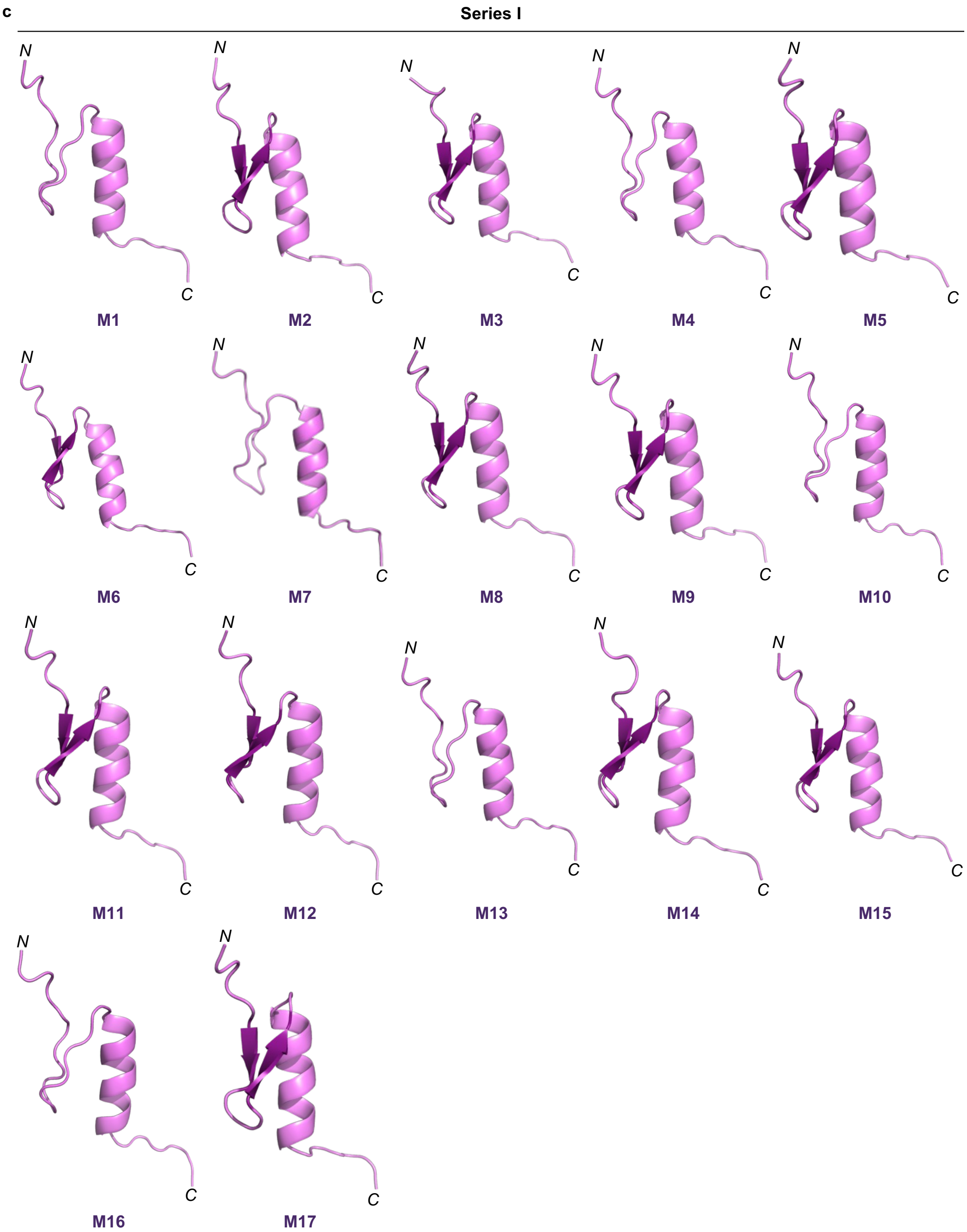
c
Series I
N
C
M1
M2
M3
M4
M5
M6
M7
M8
M9
M10
M11
M12
M13
M14
M15
M16
M17

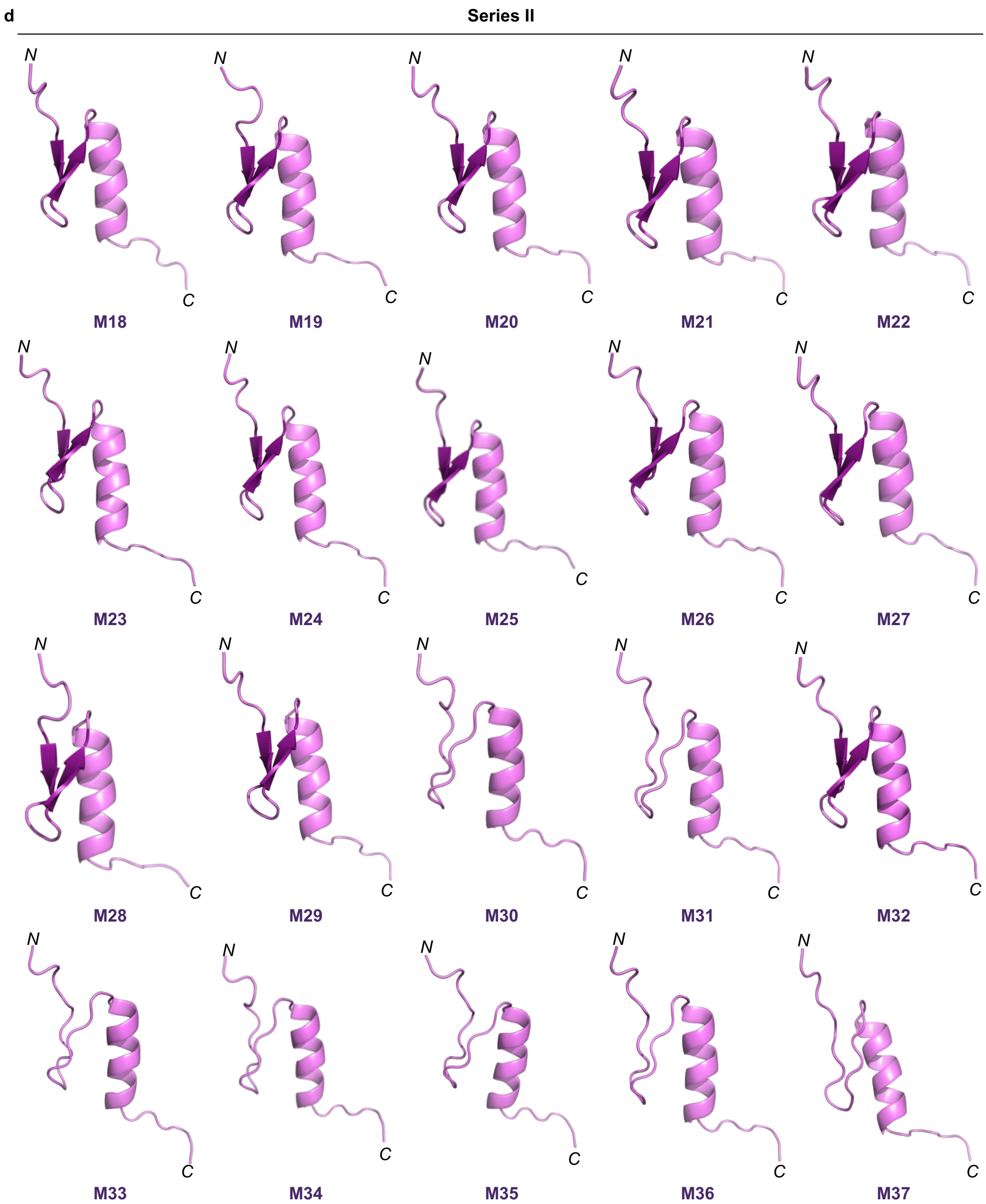
d
Series II
M18
M19
M20
M21
M22
M23
M24
M25
M26
M27
M28
M29
M30
M31
M32
M33
M34
M35
M36
M37

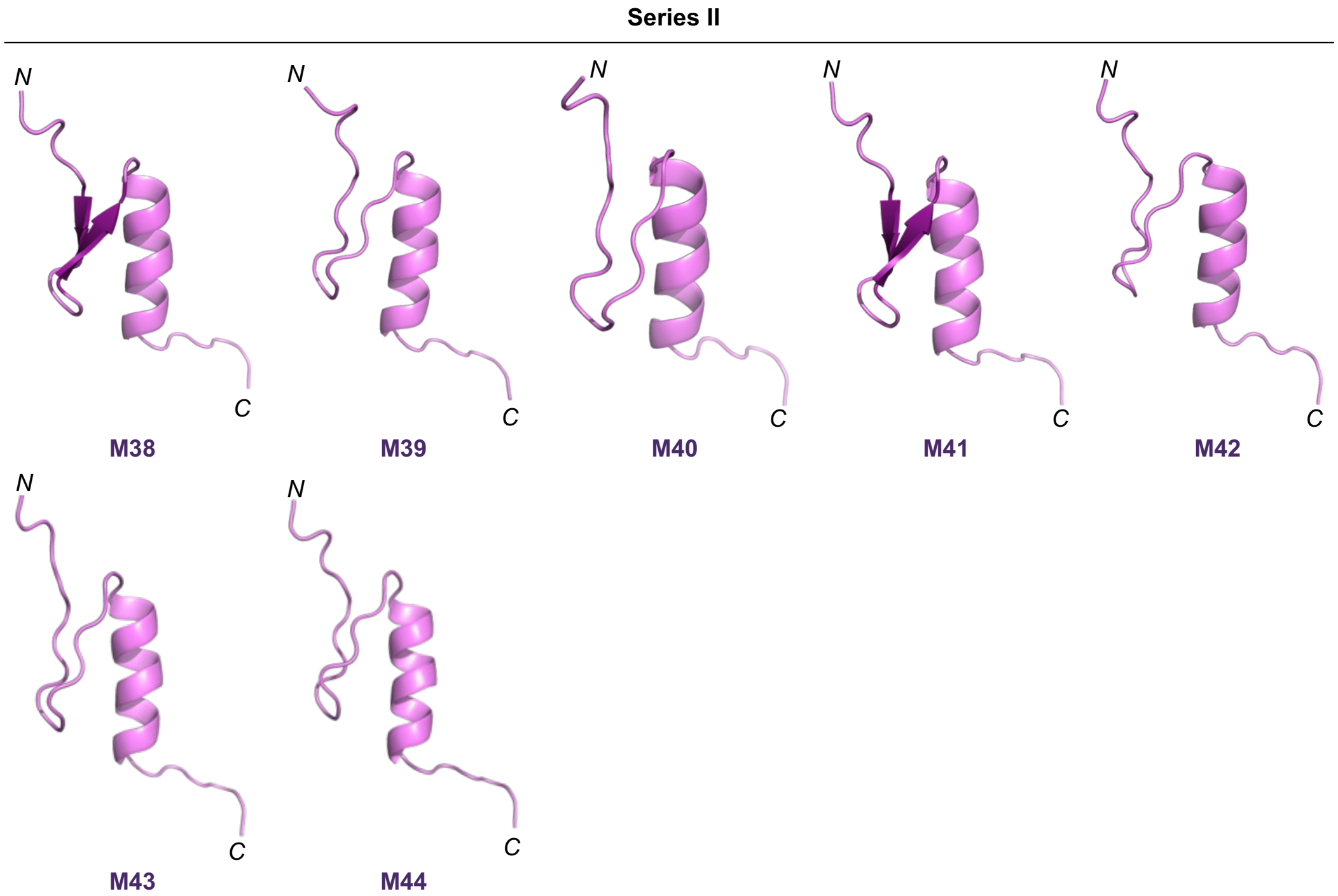

Figure S27. Amino acid sequences and AlphaFold2-predicted structures of Mx. (a,b) Amino acid sequences of Mx. M1–M44 were strategically designed by altering amino acid residues in the β_1 and β_2 regions based on the parent ZF2' framework. (c,d) Structures of Mx predicted by AlphaFold2.[4,5] It should be noted that AlphaFold2 predicts ZF structures based on databases containing Zn(II)-bound forms.

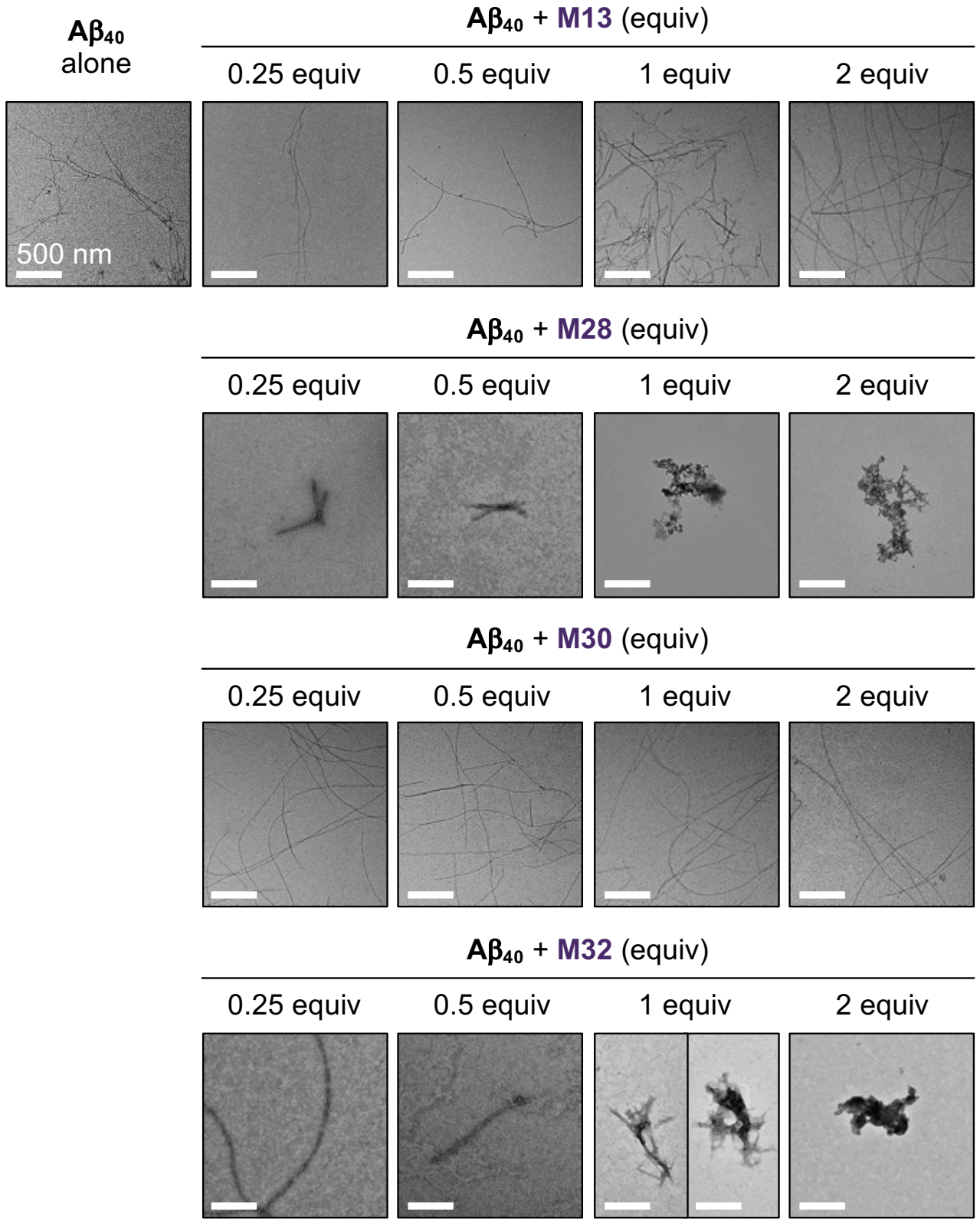

Figure S28. Morphologies of the resultant aggregates by incubation of $A\beta_{40}$ with and without Mx for 8 h monitored by TEM. Conditions: $[A\beta_{40}]$ = 100 μM; [apo-Mx] = 25, 50, 100, and 200 μM; [Zn(II)] = 22.5, 45, 90, and 180 μM; 20 mM HEPES, pH 7.4, 150 mM NaCl, 1 mM DTT; 37 °C; quiescence. It should be noted that 0.9 equiv of Zn(II) was added to apo-Mx and incubated for 3 min to minimize free Zn(II) in solution. Scale bar = 500 nm.

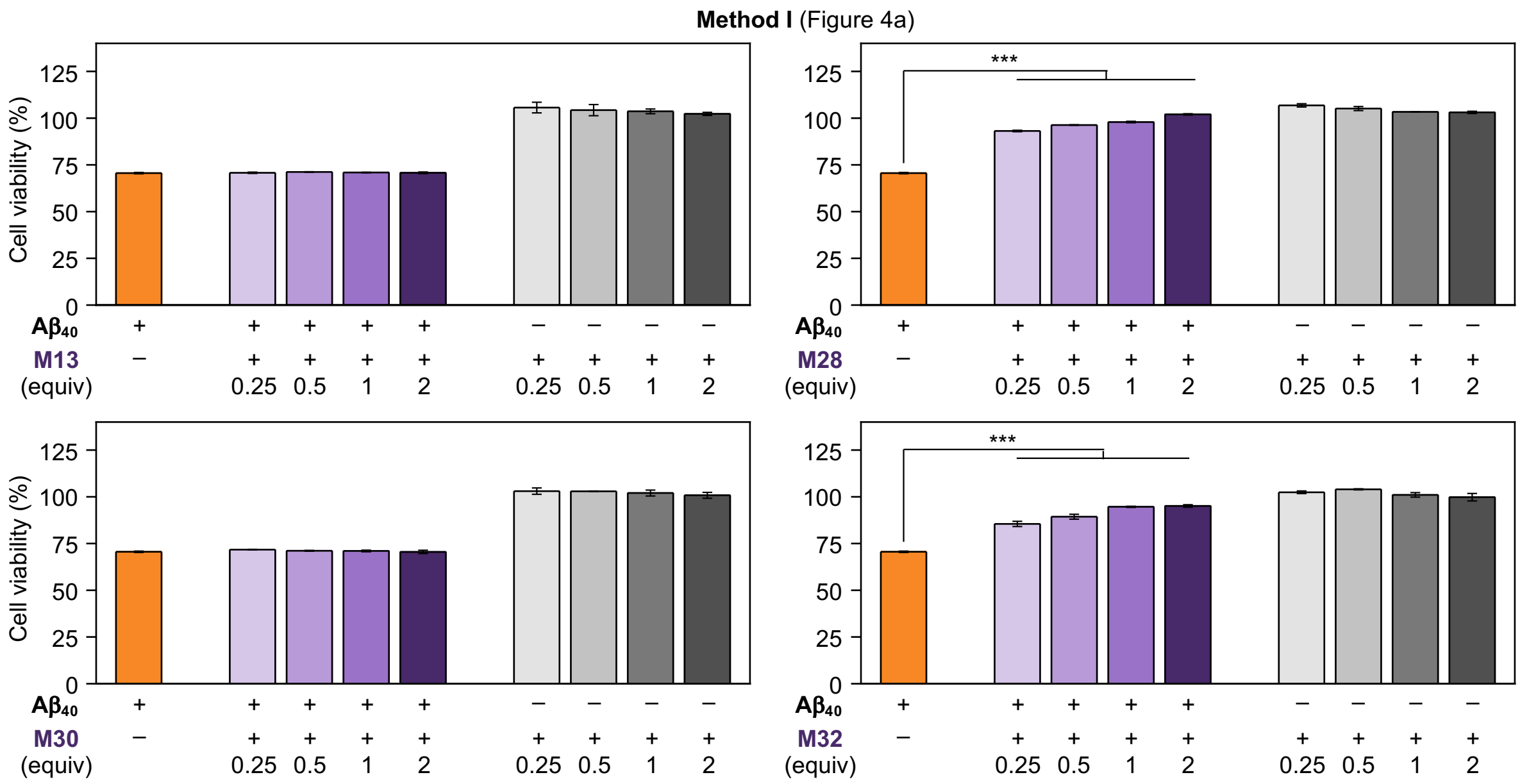

Figure S29. Influence of Mx on the cytotoxicity of Aβ_{40}. The viability of cells treated with either Aβ_{40}, selected Mx, or both was determined by the MTT assay. Cell survival was calculated in comparison to that with an equivalent amount of the buffered solution. Conditions: [Aβ_{40}] = 10 μM; [apo-Mx] = 2.5, 5, 10, and 20 μM; [Zn(II)] = 2.25, 4.5, 9, and 18 μM; 20 mM HEPES, pH 7.4, 150 mM NaCl; pre- and post-incubation for 8 h and 48 h, respectively; 37 °C; quiescence; SH-SY5Y cells that were non-differentiated. It should be noted that 0.9 equiv of Zn(II) was added to apo-Mx and incubated for 3 min to minimize free Zn(II) in solution. ***P < 0.001; n = 3; Student's t-test. Data are presented as mean ± s.e.m.

Supporting References

1. Y. Hwang, A. K. Mohammad Mydul Islam, S. Park, et al., "Decoding the Parkinson's Symphony: PARIS, Maestro of Transcriptional Regulation and Metal Coordination for Dopamine Release," *ACS Chemical Neuroscience* 15 (2024): 447–455, https://doi.org/10.1021/acschemneuro.3c00640.
2. J. Han, J. Yoon, J. Shin, et al., "Conformational and Functional Changes of the Native Neuropeptide Somatostatin Occur in the Presence of Copper and Amyloid-β," *Nature Chemistry* 14 (2022): 1021–1030, https://doi.org/10.1038/s41557-022-00984-3.
3. E. Gasteiger, A. Gattiker, C. Hoogland, I. Ivanyi, R. D. Appel, and A. Bairoch, "ExPASy: The Proteomics Server for In-Depth Protein Knowledge and Analysis," *Nucleic Acids Research* 31 (2003): 3784–3788, https://doi.org/10.1093/nar/gkg563.
4. J. Jumper, R. Evans, A. Pritzel, et al., "Highly Accurate Protein Structure Prediction with AlphaFold," *Nature* 596 (2021): 583–589, https://doi.org/10.1038/s41586-021-03819-2.
5. M. Mirdita, K. Schütze, Y. Moriwaki, L. Heo, S. Ovchinnikov, and M. Steinegger, "ColabFold: Making Protein Folding Accessible to All," *Nature Methods* 19 (2022): 679–682, https://doi.org/10.1038/s41592-022-01488-1.
6. J. Abramson, J. Adler, J. Dunger, et al., "Accurate Structure Prediction of Biomolecular Interactions with AlphaFold 3," *Nature* 630 (2024): 493–500, https://doi.org/10.1038/s41586-024-07487-w.
7. S. Vivekanandan, J. R. Brender, S. Y. Lee, and A. Ramamoorthy, "A Partially Folded Structure of Amyloid-β(1-40) in an Aqueous Environment," *Biochemical and Biophysical Research Communications* 411 (2011): 312–316, https://doi.org/10.1016/j.bbrc.2011.06.133.
8. D. Kozakov, D. R. Hall, B. Xia, et al., "The ClusPro Web Server for Protein–Protein Docking," *Nature Protocols* 12 (2017): 255–278, https://doi.org/10.1038/nprot.2016.169.
9. J. A. Maier, C. Martinez, K. Kasavajhala, L. Wickstrom, K. E. Hauser, and C. Simmerling, "ff14SB: Improving the Accuracy of Protein Side Chain and Backbone Parameters from ff99SB," *Journal of Chemical Theory and Computation* 11 (2015): 3696–3713, https://doi.org/10.1021/acs.jctc.5b00255.
10. P. Li, L. F. Song, and K. M. Merz, Jr., "Systematic Parameterization of Monovalent Ions Employing the Nonbonded Model," *Journal of Chemical Theory and Computation* 11 (2015): 1645–1657, https://doi.org/10.1021/ct500918t.
11. P. Li and K. M. Merz, Jr., "Taking into Account the Ion-Induced Dipole Interaction in the Nonbonded Model of Ions," *Journal of Chemical Theory and Computation* 10 (2014): 289–

297, https://doi.org/10.1021/ct400751u.

12. O. Melse, I. Antes, V. R. I. Kaila, and M. Zacharias, "Benchmarking Biomolecular Force Field-Based Zn(II) for Mono- and Bimetallic Ligand Binding Sites," *Journal of Computational Chemistry* 44 (2023): 912–926, https://doi.org/10.1002/jcc.27052.
13. S. Miyamoto and P. A. Kollman, "Settle: An Analytical Version of the SHAKE and RATTLE Algorithm for Rigid Water Models," *Journal of Computational Chemistry* 13 (1992): 952–962, https://doi.org/10.1002/jcc.540130805.
14. T. Darden, D. York, and L. Pedersen, "Particle Mesh Ewald: An N·log(N) Method for Ewald Sums in Large Systems," *The Journal of Chemical Physics* 98 (1993): 10089–10092, https://doi.org/10.1063/1.464397.
15. D. A. Case, T. E. Cheatham, 3rd, T. Darden, et al., "The Amber Biomolecular Simulation Programs," *Journal of Computational Chemistry* 26 (2005): 1668–1688, https://doi.org/10.1002/jcc.20290.
16. F. Delaglio, S. Grzesiek, G. W. Vuister, G. Zhu, J. Pfeifer, and A. Bax, "NMRPipe: A Multidimensional Spectral Processing System Based on UNIX Pipes," *Journal of Biomolecular NMR* 6 (1995): 277–293, https://doi.org/10.1007/bf00197809.
17. Sparky 3 (2020): https://www.cgl.ucsf.edu/home/sparky/.
18. S. J. Hyung, A. S. DeToma, J. R. Brender, et al., "Insights into Antiamyloidogenic Properties of the Green Tea Extract (-)-Epigallocatechin-3-Gallate toward Metal-Associated Amyloid-β Species," *Proceedings of the National Academy of Sciences of the United States of America* 110 (2013): 3743–3748, https://doi.org/10.1073/pnas.1220326110.
19. Y. Yi, Y. Lin, J. Han, et al., "Impact of Sphingosine and Acetylsphingosines on the Aggregation and Toxicity of Metal-Free and Metal-Treated Amyloid-β," *Chemical Science* 12 (2020): 2456–2466, https://doi.org/10.1039/d0sc04366d.
20. F. A.A. Mulder, D. Schipper, R. Bott, and R. Boelens, "Altered Flexibility in the Substrate-Binding Site of Related Native and Engineered High-Alkaline Bacillus Subtilisins," *Journal of Molecular Biology* 292 (1999): 111–123, https://doi.org/10.1006/jmbi.1999.3034.
21. P. Thordarson, "Determining Association Constants from Titration Experiments in Supramolecular Chemistry," *Chemical Society Reviews* 40 (2011): 1305–1323, https://doi.org/10.1039/C0CS00062K.
22. Y. Lin, B. R. Sahoo, D. Ozawa, et al., "Diverse Structural Conversion and Aggregation Pathways of Alzheimer's Amyloid-β (1–40)," *ACS Nano* 13 (2019): 8766–8783,

https://doi.org/10.1021/acsnano.9b01578.

23. P. Arosio, T. P. J. Knowles, and S. Linse, "On the Lag Phase in Amyloid Fibril Formation," *Physical Chemistry Chemical Physics* 17 (2015): 7606–7618, https://doi.org/10.1039/C4CP05563B.
24. A. El Aferni, M. Guettari, and T. Tajouri, "Mathematical Model of Boltzmann's Sigmoidal Equation Applicable to the Spreading of the Coronavirus (Covid-19) Waves," *Environmental Science and Pollution Research* 28 (2021): 40400–40408, https://doi.org/10.1007/s11356-020-11188-y.
25. D. D. Mruk and C. Y. Cheng, "Enhanced Chemiluminescence (ECL) for Routine Immunoblotting: An Inexpensive Alternative to Commercially Available Kits," *Spermatogenesis* 1 (2011): 121–122, https://doi.org/10.4161/spmg.1.2.16606.
26. N. J. Greenfield, "Using Circular Dichroism Spectra to Estimate Protein Secondary Structure," *Nature Protocols* 1 (2006): 2876–2890, https://doi.org/10.1038/nprot.2006.202.
27. A. Micsonai, F. Wien, L. Kernya, et al., "Accurate Secondary Structure Prediction and Fold Recognition for Circular Dichroism Spectroscopy," *Proceedings of the National Academy of Sciences of the United States of America* 112 (2015): E3095–E3103, https://doi.org/10.1073/pnas.1500851112.
28. Z. Du, E. Nam, Y. Lin, et al., "Unveiling the Impact of Oxidation-Driven Endogenous Protein Interactions on the Dynamics of Amyloid-β Aggregation and Toxicity," *Chemical Science* 14 (2023): 5340–5349, https://doi.org/10.1039/d3sc00881a.
29. Y. Nagai, T. Inui, H. A. Popiel, et al., "A Toxic Monomeric Conformer of the Polyglutamine Protein," *Nature Structural & Molecular Biology* 14 (2007): 332–340, https://doi.org/10.1038/nsmb1215.
30. K. W. Dunn, M. M. Kamocka, and J. H. McDonald, "A Practical Guide to Evaluating Colocalization in Biological Microscopy," *American Journal of Physiology–Cell Physiology* 300 (2011): C723–C742, https://doi.org/10.1152/ajpcell.00462.2010.
31. J. Mensch, A. Melis, C. Mackie, G. Verreck, M. E. Brewster, and P. Augustijns, "Evaluation of Various PAMPA Models to Identify the Most Discriminating Method for the Prediction of BBB Permeability," *European Journal of Pharmaceutics and Biopharmaceutics* 74 (2010): 495–502, https://doi.org/10.1016/j.ejpb.2010.01.003.
32. A. Avdeef 2012, "Chapter 7-Premeability–PAMPA." in *Absorption and Drug Development: Solubility, Permeability, and Charge State*. Second Ed., John Wiley & Sons, Inc. ISBN 978-1-118-05745-2.
33. L. Di, E. H. Kerns, K. Fan, O. J. McConnell, and G. T. Carter, "High Throughput Artificial

Membrane Permeability Assay for Blood-Brain Barrier," *European Journal of Medicinal Chemistry* 38 (2003): 223–232, https://doi.org/10.1016/s0223-5234(03)00012-6.

34. A. Avdeef, S. Bendels, L. Di, et al., "PAMPA–Critical Factors for Better Predictions of Absorption," *Journal of Pharmaceutical Sciences* 96 (2007): 2893–2909, https://doi.org/10.1002/jps.21068.
35. J. L. Jankowsky, D. J. Fadale, J. Anderson, et al., "Mutant Presenilins Specifically Elevate the Levels of the 42 Residue β-Amyloid Peptide In Vivo: Evidence for Augmentation of a 42-Specific γ Secretase," *Human Molecular Genetics* 13 (2004): 159–170, https://doi.org/10.1093/hmg/ddh019.
36. H. M. Jackson, K. D. Onos, K. W. Pepper, et al., "DBA/2J Genetic Background Exacerbates Spontaneous Lethal Seizures but Lessens Amyloid Deposition in a Mouse Model of Alzheimer's Disease," *PLoS ONE* 10 (2015): e0125897, https://doi.org/10.1371/journal.pone.0125897.
37. G. S. Finnie, R. Gunnarsson, J. Manavis, et al., "Characterization of an 'Amyloid Only' Transgenic (B6C3-Tg(APPswe,PSEN1dE9)85Dbo/Mmjax) Mouse Model of Alzheimer's Disease," *Journal of Comparative Pathology* 156 (2017): 389–399, https://doi.org/10.1016/j.jcpa.2017.03.001.
38. T.-S. Park, Y.-K. Ryu, H.-Y. Park, et al., "Humulus Japonicus Inhibits the Progression of Alzheimer's Disease in a APP/PS1 Transgenic Mouse Model," *International Journal of Molecular Medicine* 39 (2017): 21–30, https://doi.org/10.3892/ijmm.2016.2804.
39. J. Go, D.-H. Chang, Y.-K. Ryu, et al., "Human Gut Microbiota Agathobaculum Butyriciproducens Improves Cognitive Impairment in LPS-Induced and APP/PS1 Mouse Models of Alzheimer's Disease," *Nutrition Research* 86 (2021): 96–108, https://doi.org/10.1016/j.nutres.2020.12.010.

Author Contributions

S.P., S.J.L., and M.H.L. designed the research. S.P. performed spectroscopic measurements (absorbance, fluorescence, DLS, and CD), PAGE, biochemical assays, ICC studies, protein–protein docking studies, and AlphaFold2/3 modeling with data analysis. Y.H., S.P., H.K., H.G.K., and S.J.L. conducted the expression and purification of ZF2–4, ^{15}N-labeled ZF2–4, ^{HF555}ZF2–4, ^{HF647}ZF2–4, and ZF2–4–P9. S.P. and J.K. carried out LC–MS measurements and analyzed the data. J.K., E.N., and J.H. performed TEM measurements and live-cell studies. Y.L. and Y.-H.L. carried out 2D ^{1}H–^{15}N HMQC and HSQC NMR and ITC experiments and analyzed the data. H.-Y.P., J.-E.K., K.-S.K., and C.-H.L. conducted *in vivo* experiments, including ZF2–4–P9 administration, the sample preparation for biodistribution studies, immunohistochemistry, Western blotting, quantitative RT–PCR, and behavioral testing, and data analysis. J.P. carried out MD simulations with analysis. S.P., J.K., J.P., and M.H.L. wrote the manuscript with input from all authors.
