# Supplementary material for: Zinc Fingers as Programmable Metalloprotein Scaffolds for Altering Pathogenic Biomolecular Phase Transitions
Source: Adv Sci (Weinh). 2026 Sep 30:e77886. Online ahead of print. doi: 10.1002/advs.77886 (PMC13624870; doi:10.1002/advs.77886)
Supplement: Supplementary file 2 [file ADVS-9999-e77886-s001.docx]

**Video S1.** MD simulations of Model 11 focusing on the ZF2 segment of ZF2–4 observed over 500 ns. The trajectories of MD simulations showed the conformational change in the ZF2 region of ZF2–4 upon binding to Aβ_40_. The original β-hairpin-containing region is indicated with a dashed black box.
